# PLCβ1 by-passes early growth response -1 to induce the differentiation of neuronal cells

**DOI:** 10.1038/s41420-024-02009-z

**Published:** 2024-05-24

**Authors:** Imanol González-Burguera, Guanyu Lin, Maider López de Jesús, Miquel Saumell-Esnaola, Sergio Barrondo, Gontzal García del Caño, Joan Sallés, Suzanne Scarlata

**Affiliations:** 1 Department of Neurosciences, Faculty of Pharmacy, University of the Basque Country (UPV/EHU), 01006 Vitoria‐Gasteiz, Spain; 2 Bioaraba, Neurofarmacología Celular y Molecular, 01006 Vitoria-Gasteiz, Spain; 3 Department of Chemistry and Biochemistry, Worcester Polytechnic Institute, Worcester, Massachusetts 01609 USA; 4 Department of Pharmacology, Faculty of Pharmacy, University of the Basque Country (UPV/EHU), 01006 Vitoria‐Gasteiz, Spain; 5 Centro de Investigación Biomédica en Red de Salud Mental (CIBERSAM), 28029 Madrid, Spain

**Keywords:** Neurophysiology, Cell biology

## Abstract

The Gα_q_/phospholipase C-β (PLCβ) signaling system mediates calcium responses to a variety of hormones and neurotransmitters. Recent studies suggest that PLCβ1 expression plays a role in the differentiation of two types of cultured neuronal cells (PC12 and SK-N-SH) through a mechanism independent of Gα_q_. Here, we show that, similar to that observed in PC12 and SK-N-SH cells, PLCβ1 expression increases when human NT2 cells are induced to differentiate either through cytosine-β-D-arabinofuranoside or retinoic acid. Preventing this increase, abolishes differentiation, and down-regulating PLCβ1 in rat primary astrocytes causes cells to adapt an undifferentiated morphology. Surprisingly, transfecting PLCβ1 into undifferentiated PC12 or NT2 cells induces differentiation without the need for differentiating agents. Studies to uncover the underlying mechanism focused on the transcription factor early growth response 1 (Egr-1) which mediates PLCβ1 expression early in differentiation. Over-expressing PLCβ1 in HEK293 cells enhances Egr-1 expression and induces morphological changes. We show that increased levels of cytosolic PLCβ1 in undifferentiated PC12 cells disrupts the association between Egr-1 and its cytosolic binding partner (Tar RNA binding protein), promoting relocalization of Egr-1 to the nucleus, which promotes transcription of proteins needed for differentiation. These studies show a novel mechanism through which differentiation can be modulated.

## Introduction

The ability of stem cells to differentiate into a neuronal phenotype offers promise for therapies to replace damaged neural tissue, and thus, understanding the parameters that control differentiation would help develop more effective methods. Cultured cell models, such as PC12, have been invaluable in delineating differentiation pathways. Although not neuronal in origin, PC12 cells can be induced to differentiate into cells that are morphologically and functionally similar to neuroendocrine cells [1]. PC12 cells are an attractive model for neuronal cell development because their differentiation occurs over a short period of time (i.e., ~36 h) as compared to other cell lines such as NTERA2/D1 (NT2) cells that take from one to more than 4 weeks depending on the inducing agent used [2, 3]. Like PC12, NT2 are not neuronal in origin but have similar properties to human embryonic cells and been used as models for embryonic neurogenesis. NT2 cells can be differentiated by treatment with retinoic acid (RA) during relatively long periods (more than 4 weeks) to produce cells with neuronal characteristics [4] along with a smaller population of glial-like cells [5, 6]. It is noteworthy that terminally differentiated NT2-derived neurons (NT2N) maintain their neuronal phenotype after engraftment in the rodent brain and spinal cord [7, 8], and have been shown to ameliorate motor deficits in animal models of stroke [9] and Huntington’s disease [10]. Moreover, NT2N neurons have been proposed as a platform for ex vivo gene and cell replacement therapies of neurological disorders [7, 11–13], as clinical trials have demonstrated their integration in human brain tissue for prolonged periods without tumourigenicity [11, 12, 14, 15]. In addition, NT2 cells can also differentiate within a shorter period of approximately one week into post-mitotic NT2N neurons by exposure to cytosine-β-D-arabinofuranoside (AraC) [3] through a mechanism involving degradation of stem cell-specific proteins by caspases [6], thus providing a versatile cell model for time-scale studies compatible with transient over-expression or gene silencing.

Many differentiation agents work by activating the inducible transcription factor early growth response-1 protein (Egr-1). Egr-1 is a member of the Egr family of immediate early genes (IEGs), whose mRNA levels rapidly and transiently increase in response to a variety of environmental stimuli (for review see [16]). Egr-1 is a general transcription factor and the induction of *EGR1* transcription depends on the nature of the tissue and growth factor. Egr-1 is induced by many different mitogens including growth factors (e.g., NGF), serum and cytokines (see [17, 18]), and has been shown to be responsible for 3D matrix mechanosensitive neural stem cell fate [19].

Exposure of PC12 cells to nerve growth factor (NGF) and NT2 cells to RA to induce neuronal differentiation activate Egr-1, which in turn stimulates the production of proteins required for differentiated, non-proliferative cells such as phospholipase C-β1 (PLCβ1) and Gα_q_ [20, 21].

PLCβ enzymes are one of the six mammalian classes of the large family of inositol-specific PLCs (PtdIns-PLCs) that hydrolyze the signaling lipid phosphoinositol-4,5-bisphosphate (PIP_2_) to generate the second messengers, inositol 1,4,5 trisphosphate (Ins 1,4,5-P_3_ or IP_3_) and diacylglycerol (DAG), leading to an increase in intracellular calcium and activation of protein kinase C [22–24]. In the brain, the most prominent PtdIns-PLCs are PLCβs, which are activated by Gα_q_ in response to neurotransmitter such as serotonin and acetylcholine, and PLCγs, which are activated by receptor tyrosine kinases in response to growth factors such as brain-derived growth factor (BNDF) and nerve growth factor (NGF) [25].

Our labs study PLCβ enzymes, which are uniquely activated by the Gα_q_ family of G proteins [22–24, 26]. Gα_q_ is coupled to receptors that bind to neurotransmitters such as acetylcholine and serotonin [27]. There are four known PLCβs and PLCβ1 is the main PLCβ in neuronal cells [28, 29]. PLCβ1 exists as two variants, the more prevalent PLCβ1a form and the PLCβ1b form, in which the 75 C-terminal residues of PLCβ1a are replaced by 32 residues [30]. While both variants have identical Gα_q_ activation profiles, the unique amino acids in PLCβ1b can result in its membrane versus cytosolic localization and its specific activation by α_1_-adrengeric receptors in cardiomyocytes [31]. However, as shown here, both variants are hihgly expressed in the cytosol of neuronal cells lines.

The major pool of PLCβ1 localizes on the plasma membrane where it associates with Gα_q_ (e.g. [32]), and additionally, both variants can be found in the cytosol. This cytosolic population can bind to the component 3 promoter of RNA-induced silencing complex (C3PO) and inhibit its activity [33, 34]. Further, this inhibition of C3PO activity can reverse mRNA silencing induced by treatment with siRNAs, and shift the populations of microRNAs (miRs) associated with cell proliferation and differentiation [35, 36] through a mechanism independent of its catalytic activity. It is notable that both variants of PLCβ1 have nuclear localization signals [37] and catalytically active PLCβ1s have been reported in rat brain [38] as well several cell lines where they can modulate cell cycle and may be linked to differentiation and cancer [39–42].

Here, we have studied the impact of PLCβ1 on cell differentiation. Differentiation of PC12 cells is associated with rapid increase in PLCβ1 levels followed by increased Gα_q_ [35]. Differentiation of NT2 progenitors into NT2N mature neurons is associated with increased expression of Gα_q/11_, PLCβ1 and PLCβ4, whereas PLCβ3 and PLC-γ1 is markedly reduced [43]. This same increase is seen in PC12 cells induced to differentiate by activation of TrK receptors and subsequent activation of PLCγ [44]. However, as described below, PLCβ1 appears to play a unique role in differentiation.

PLCβ1 is strongly regulated during development of human cortex, being almost absent in fetal cerebral cortex and highly expressed in adults [45], while PLCβ1 knockout mice exhibit developmental alterations in the formation of the so-called “barrels” of the somatosensory cortex [46]. These data, together with compelling evidence that PLCβ1 promotes differentiation in different cell models [47], point to a key role for PLCβ1 in neuronal differentiation.

While the role of PLCβ1 in cell differentiation is thought to be due to its ability to hydrolyze PIP_2_ on the plasma or nuclear membranes, there is evidence that the cytosolic pool of PLCβ1 impacts differentiation through a mechanism that is independent of its lipase activity [35]. In PC12 cells induced to differentiate by NGF treatment, PLCβ1 expression rises sharply in the first 12–24 h. In contrast, the rise in Gα_q_ levels is delayed until 36–48 h. During the first 24 hours, fluorescence studies show a cytosolic population of PLCβ1 that interacts with C3PO, and this population decreases as Gα_q_ rise, recruiting PLCβ1 to the plasma membrane. The importance of PLCβ1/C3PO association in PC12 and SK-N-SH cell differentiation has been noted in studies where down-regulating PLCβ1 or C3PO prevents differentiation [36], and down-regulating PLCβ1 or C3PO in differentiated PC12 and SK-N-SH cells returns them to an undifferentiated state as indicated by loss of neuronal morphology and production of stem cell markers [45], as well as the return of proliferation [21]. Complementary RNAseq studies indicate that PLCβ1 down-regulation alters miRs associated with proliferation and development [36], suggesting that the ability of PLCβ1 to inhibit C3PO impacts differentiation.

Here, we show that PLCβ1 is a necessary and sufficient element to induce differentiation of NT2 and PC12 cells potentially through a mechanism that disrupts the association of Egr-1 with cytosolic binding partners promoting its movement to the nucleus and the expression of genes involved in differentiation. This novel signaling pathway involving PLCβ1 may create new approaches to modulate differentiation.

## Results

### Differentiation of NT2 cells results in a large increase in PLCβ1 expression

**Fig. 1:**
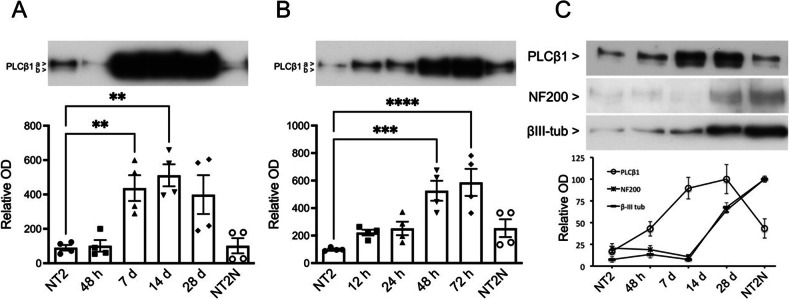
Changes in PLCβ1 levels in NT2 cells during differentiation by RA and AraC. **A** NT2 cells progenitors were treated with RA and immunoblotted with anti-PLCβ1a and PLCβ1b antibodies at various times (48 h, 7 d, 14 d, 28 d). Terminally differentiated NT2N cells were isolated from cultures at 28 d of RA exposue by mechanical dislogding (see Methods). Equal amounts of total protein (12 μg) were loaded (*n* = 4). **B** Similar studies as in (**A**) except NT2 progenitors were treated with AraC at various times (12, 24, 48 and 72 h and 6 days -NT2N cells-) where time points were chosen from previous studies [3]. Equal amounts of total protein (12 μg) were loaded. The optical density (OD) is expressed as the percentage of immunoreactive signal found in NT2 progenitor cells (100%). Repeated measures one-way ANOVA followed by Tukey’s post hoc test. Significant differences between the different samples with respect to NT2 progenitors are shown (*p < 0.05; **p < 0.01; ***p < 0.001). Data are mean ± SEM (*n* = 4). **C** Immunoblots of PLCβ1 on whole homogenates of NT2 progenitors, AraC-treated cells, and AraC/NT2N cells and NF200 and βIII-tubulin. Equal amounts of total protein (12 μg) were loaded.

Previous studies showed that levels of PLCβ1 are very low in undifferentiated PC12 cells and increase dramatically (i.e., ~5 fold) after treatment with NGF [35]. Here, we determined whether this increase in PLCβ1 also occurs during neuronal differentiation of NT2 cells. We first followed levels of PLCβ1 after treatment with retinoic acid (RA) which promotes differentiation through PI3K/Akt and ERK1/2 signaling cascades [48–50]. Following changes in PLCβ1 levels in NT2 cells after RA treatment, we observe a sharp increase in PLCβ1 expression after one week that remains elevated (Fig. 1A). Because NT2 cells differentiate into neuronal (NT2N) and non-neuronal (NT2A) phenotypes, we mechanically separated the two phenotypes (see Methods) and found that PLCβ1 levels returns to basal in terminally differentiated NT2N neurons but remains elevated in non-neuronal NT2A cells. Repeating this study using AraC, which induces differentiation over a much shorter duration than RA and through a distinct mechanism [3, 4, 48] we found a similar, progressive increase in PLCβ1in the early stages of differentiation that returns to basal levels in terminally differentiated NT2N neurons (Fig. 1B). It is interesting to note that the peak expression of PLCβ1 precedes the neuronal phenotype markers neurofilament 200 (NF200) and β-III tubulin, (Fig. 1C) suggesting a role of PLCβ1 early in the differentiation processes.

PLCβ1 exists as two splice variants PLCβ1a and PLCβ1b whose localization may vary in different cell lines [24, 51]. Thus, we analyzed the expression of these variants through the course of RA- and AraC-induced differentiation. As expected from previous studies (e.g., [30]), PLCβ1a is more highly expressed than PLCβ1b but the PLCβ1a/PLCβ1b ratio does not vary during RA- or AraC-induced differentiation (Fig. S1A, B), showing that the increase in PLCβ1 is not specific to one of the variants.

**Fig. 2:**
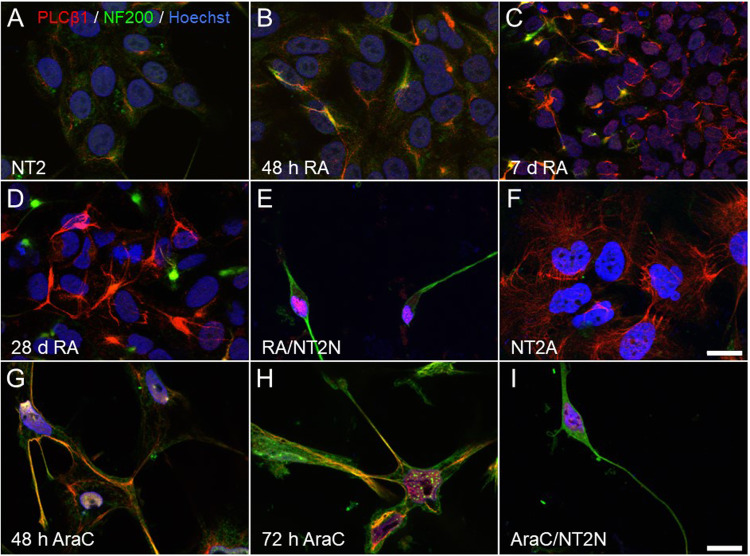
Visualization of PLCβ1 and NF200 during RA and AraC differentiation of NT2 cells. Immunofluorescence images using anti-PLCβ1 (red), NF200 (green) and Hoechst’s chromatin staining (blue) of NT2 progenitors (**A**) and after treatment with RA (**A**–**F**) or AraC (**G**–**I**). Cells treated with RA were fixed and immunostained at 48 h (**B**), 7 days (**C**), 28 days (**D**), and after isolation of terminally differentiated NT2N neurons by mechanical dislodging (**E**). Non-neuronal cells (NT2A) that remained attached to the culture flask after the mechanical isolation of NT2N neurons were also doubly immunostained for PLCβ1 and NF200 (**F**). Cells treated with AraC were fixed at 48 h (**G**), 72 h (**H**) and 6 days, when cells were differentiated into NT2N neurons (**I**). Images are maximum intensity projections of four consecutive optical sections separated by 0.24 µm and obtained using a structured illumination module. Scale bar in **F** = 20 µm (applies to **A**–**F**). Scale bar in **I** = 20 µm (applies to **G**–**I**).

Next, we followed the localization of PLCβ1 during differentiation of NT2 cells by immunofluorescence. In RA-treated cells (Fig. 2A–F), PLCβ1 immunofluorescence increases around 48 h when cells begin to express the neuronal marker NF200. After 7 days of RA exposure, PLCβ1 immunofluorescence increases in all cells, whereas NF200 increases in only a small proportion (Fig. 2C). On day 28 of treatment, PLCβ1 immunofluorescence remains elevated in non-neuronal, NF200-negative cells (Fig. 2D). At the end of RA treatment, neuronal NT2N neurons (Fig. 2E), purified from non-neuronal NT2A cells by mechanical separation (Fig. 2F), showed a reduction in PLCβ1 expression coinciding with the acquisition of a neuronal fate, which is a trend shared with PC12 cells [35]. Similarly, AraC treatment caused a sharp increase in PLCβ1-immunofluorescence in neurites after 48 and 72 h treatment (Fig. 2G, H) and was reduced in terminally differentiated NT2N neurons (Fig. 2I). Taken together, these studies show that differentiation of NT2 cells by RA or AraC is accompanied by an initial increase in PLCβ1 levels similar to that observed in NGF-induced differentiation of PC12 cells [35] and suggest that this phenomenon is general to neuronal differentiation and independent of progenitor type.

PLCβ4 isoform has been reported to be up-regulated during RA-induced neuronal differentiation of NT2 cells [43]. To rule out a possible cross-interaction with PLCβ1-mediated signaling, we analyzed the expression of PLCβ4 in the AraC-induction model, and could not detect significant changes during differentiation supporting a direct correlation between PLCβ1 levels and differentiation (Fig. S2).

### PLCβ1 is necessary to transition and maintain normal differentiation

**Fig. 3:**
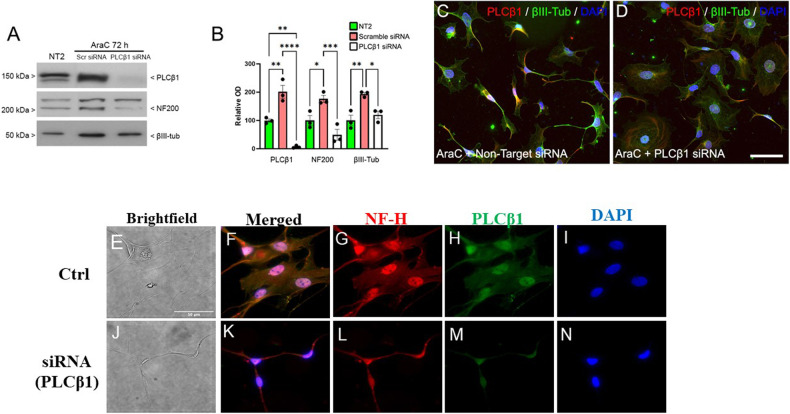
Down-regulation of PLCβ1 reduces AraC-induced NT2 cell differentiation. Sample Western blot (**A**) and densitometric analysis (**B**) of PLCβ1, NF200 and βIII-tubulin expression in NT2 cells transfected either with scrambled off-target siRNA or siRNA targeting the *PLCB1* transcript (PLCβ1 siRNA) and treated with AraC for 72 h. Equal amounts of total protein (30 μg) were loaded on the same gel and run in parallel. The *y*-axis of the graph shows the mean optical density (OD) as a percentage of that in NT2 cells (100%). One-way ANOVA followed by post hoc Tukey’s test (*p < 0.05; **p < 0.01; ***p < 0.001). Data are mean ± SEM (*n* = 4). **C**–**D** Immunofluorescence visualizing PLCβ1 (red) and βIII-tubulin (green) combined with Hoechst’s chromatin staining (blue) in NT2 cells after transfection with either scrambled siRNA (**C**) or PLCβ1 siRNA (**D**) and treated with AraC for 72 h. Scale bar = 100 µm (applies to **C**, **D**). **E–N** Immunofluorescence studies of primary rat astrocytes under control conditions or treated with si(RNA)PLCβ1a where NF-H refers to the heavy subunit of neurofilament. More images can be found in Fig. S4G. Scale bar = 50 μm.

We tested whether increased levels of PLCβ1 are essential for neuronal differentiation of NT2 cells, as previously observed in PC12 and SK-N-SH cells [45], and not an epiphenomenon of differentiation. These studies were conducted by testing the ability of AraC to induce neuronal differentiation of NT2 cells in which PLCβ1 was down-regulated by either double-stranded small interfering RNAs (siRNAs) or small hairpin RNAs (shRNAs), whose silencing efficiency was tested (Figs. S3A–C). NT2 cells were transfected with mixtures of 4 siRNAs or 4 shRNAs targeting the human *PLCB1* gene and treated with AraC to induce differentiation. Western blot studies showed that down-regulating PLCβ1 in AraC-treated NT2 cells prevents both the up-regulation of the neuronal markers NF200 and βIII-tubulin (Fig. 3A, B) and the acquisition of neuronal morphology (Figs. 3C, S4A–F). These results for NT2 progenitors, in addition to previous findings in PC12 and SK-N-SH cells [45], confirm that increased PLCβ1 expression is necessary for neuronal differentiation.

We have previously found that reducing the cytosolic level of PLCβ1a in differentiated PC12 or SK-N-SH cells returns cells to the undifferentiated state as seen morphologically and by the presence of stem cell markers [45]. We wondered whether the same behavior could be seen in primary astrocytes. For these studies, we treated cells with siRNA (PLCβ1) and characterized their morphology and changes in the levels of the neuronal marker NF200. Unexpectedly, we found that the treated cells transition to a smaller elongated neuronal-like morphology unlike the control cells (Fig. 3E–N; where an extended study is shown in Fig. S4G). This result correlates to the low levels of PLCβ1 found in NT2 neurons and shows how PLCβ1 levels may impart unique morphologies in these primary cells.

### PLCβ1 over-expression promotes NT2 and PC12 cell differentiation in the absence of differentiation inducers

**Fig. 4:**
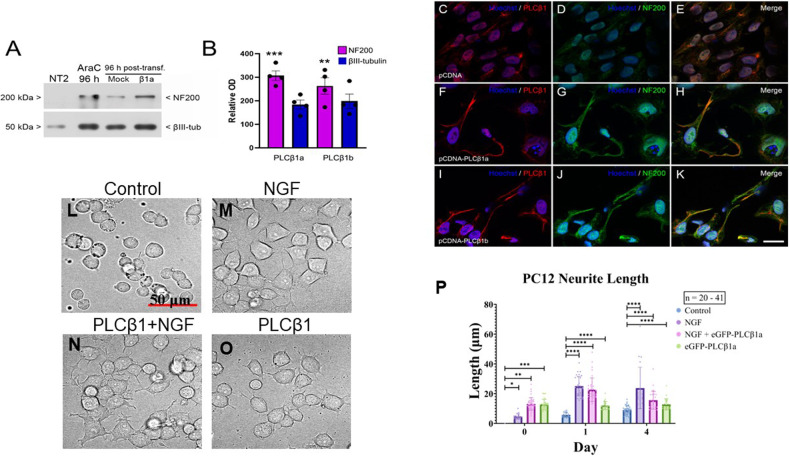
Transfection of PLCb1 into undifferentiated NT2 and PC12 cells promotes the differentiated state. **A**–**K** Studies of NT2 cells. **A**–**B** Representative immunoblots (**A**) and densitometric analysis (**B**) for NF200 and βIII-tubulin in homogenates from NT2 cells transfected with either empty pcDNA, pcDNA-PLCβ1a or pcDNA-PLCβ1b and treated with AraC for 96 h. Equal amounts of total protein (5 μg) were loaded. The *y*-axis in **B** shows the mean optical density (OD) as a percentage of that in NT2 cells (100%). Significance was assessed by one-way ANOVA followed by post hoc Tukey’s test (*p < 0.05; **p < 0.01; ***p < 0.001). Data are mean ± SEM (*n* = 4). **C**–**K** are immunofluorescence images of NT2 progenitor cells transfected with empty pCDNA (**C**–**E**), pcDNA-PLCβ1a (**F**–**H**) or pcDNA-PLCβ1b (**I**–**K**) fixed 96 h after transfection and immunostained with anti-PLCβ1 (red) and anti-NF200 (green) antibodies combined with Hoechst’s chromatin staining (blue). Micrographs are maximum intensity projections of four consecutive optical sections, separated by 0.24 µm, obtained using a structured illumination module. Scale bar = 25 µm (applies to **C**–**K**). **L**–**P** Results showing that β over-expression of PLCβ1 induces PC12 cell differentiated morphology, where **L** shows DIC images of mock transfected cells, **M** cells 24 h after NGF treatment, **N** cells transfected with PLCβ1a for 24 h and subsequently treated with NGF for 24 h, and **O** cells over-expressing PLCβ1. Scale bar = 50 μm (applies to **L**–**O**). **P** Box plots compiling changes in neurite length, where *n* = 20–41 from 4 independent experiments and significance was assessed by unpaired *t* test: *p < 0.05, **p < 0.01, ***p < 0.001, ****p < 0.0001.

The requirement of PLCβ1 for transitioning to and maintaining differentiation, and the observation that this elevation precedes the increase of neuronal lineage markers (Fig. 1C) prompted us to test the possibility that over-expression of PLCβ1 can promote differentiation in the absence of inducers. We tested this idea by transfecting cells with PLCβ1a and PLCβ1b. In NT2 cells, we found that increasing PLCβ1a or PLCβ1b led to the up-regulation of the neuronal marker proteins NF200 and βIII-tubulin (Fig. 4A, B) and caused the cells to adapt a neuronal morphology. (Fig. 4C–K) correlating with the idea that PLCβ1 induces differentiation.

We then tested the impact of PLCβ1 on cell proliferation of NT2 cells. These studies were done by immunostaining with Ki67, which is expressed during the cell cycle but not when cells exit and go into the Go phase. We found that over-expression of PLCβ1a or PLCβ1b led NT2 cells to exit the cell cycle. Specifically, we found that over-expression of either PLCβ1splice variant resulted in a drastic decrease in the ratio of Ki67-positive cells compared to control cells transfected with the empty vector (control, 0.77 ± 0.01 SEM; PLCβ1a, 0.14 ± 0.02; PLCβ1b, 0.19 ± 0.02) (Fig. S6). Thus, increasing PLCβ1a or PLCβ1b stops the proliferation of NT2 cells, as well as induces their neuronal differentiation (Fig. S6).

We have previously found that in undifferentiated PC12 cells, cytosolic PLCβ1 can bind and inhibit cyclin-dependent kinase 16, thereby inhibiting the progression of cells from the G1 to S phase [21]. Here, we complemented these studies showing that transfecting PC12 cells with PLCβ1a increases nuclear ERK1/2 levels, which consistent with a loss of proliferation (Fig. S5) [52]. Additionally, we found that over-expressing PLCβ1a in undifferentiated PC12 cells induces morphological differentiation characterized by neurite outgrowth (Fig. 4L–P). Taken together, our results show that increased PLCβ1 expression is necessary and sufficient to promote neuronal differentiation of NT2 and PC12 cells.

### PLCβ1 over-expression promotes Egr-1 localization into the nucleus

We explored the mechanism that underlies the ability of PLCβ1 to induce differentiation. *Egr-1* is one of the genes activated by NGF and its protein product contributes to the preferential expression of ERK signaling target genes in response to NGF [20, 53]. Egr-1 binds to the promoter of *PLCB1*, increasing its activity and thus the synthesis of the PLCβ1 transcript [20, 53]. Because Egr-1’s role in driving differentiation and increasing PLCβ1 expression [20, 53], we tested the possibility that transfection of cells with PLCβ1 in some way changes the ability of Egr-1 to promote differentiation.

**Fig. 5:**
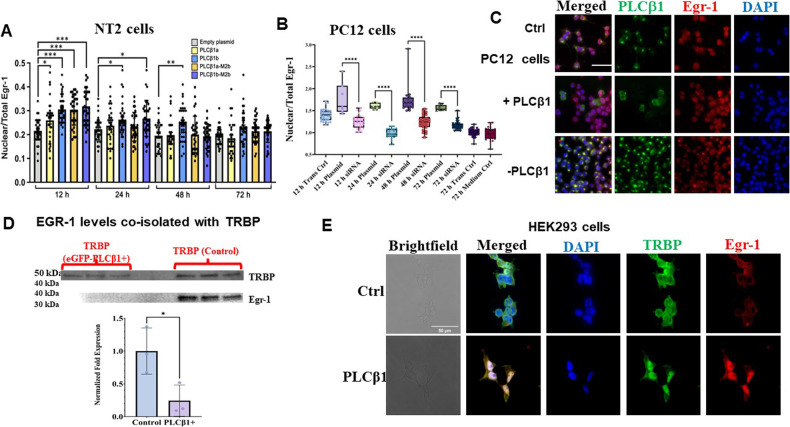
Over-expression of PLCb1 in undifferentiated NT2 and PC12 cells increases nuclear localization of Egr-1. **A** Changes in the nuclear versus total cell intensity of Egr-1 after transfection of NT2 cells with empty plasmid or different PLCβ1 constructs. Data are mean ± SEM (*n* = 33). Statistical significance was assessed by one-way ANOVA followed by post hoc Tukey’s test (*p < 0.05; **p < 0.01; ***p < 0.001). **B**–**C** Box plots and representative immunofluorescence images showing Egr-1 nuclear levels in PC12 cells exposed to different treatments, including transfection medium alone, complete medium alone and transfection medium combined with complete medium. Plots show min to max vaules as well as individual data points. Significance was assessed by unpaired *t* test: ns (*P* > 0.05), * (*P* ≤ 0.05), ** (*P* ≤ 0.01), *** (*P* ≤ 0.001), and **** (*P* ≤ 0.0001). Scale bar in **C** = 50 µm. **D** Data showing that upregulation of PLCβ1 in PC12 cells causes the dissociation between TRBP and Egr-1 where TRBP was immunoprecipitated from PC12 cell lysates and Egr-1 levels were quantified by Western blotting. Bar graph shows mean values ± SD as well as individual data points. Unpaired *t* test was used to assess statistical significance (*p < 0.05, **p < 0.01, ***p < 0.001, ****p < 0.0001). **E** Immunofluorescence images probing changes in TRBP and Egr-1 in control HEK293 cells and cells induced to over-express PLCβ1. Scale bar in **E** = 50 µm.

Egr-1 levels are reported to initially increase at the beginning of differentiation and decrease towards the end [53]. We followed Egr-1 levels in NT2 cells after AraC treatment and found a large increase (Fig. S7A) that precedes PLCβ1 up-regulation as seen in Fig. 1 [20]. Interestingly, a second smaller increase in Egr-1 which does not appear linked to PLCβ1 was observed (Fig. S7A). We then followed changes in Egr-1 levels when differentiation is induced by increasing PLCβ1. While Egr-1 decreased with PLCβ1 over-expression in both NT2 and PC12 cells (Fig. S7B, C), its cellular localization changed. Specifically, we found that a significant population of Egr-1 moved from the cytosol to the nucleus with increased PLCβ1 expression in NT2 (Fig. 5A) and PC12 cells (Fig. 5B, C). Note that a similar shift in localization was seen when undifferentiated PC12 cells were treated with NGF. From these results, we speculate that PLCβ1 may induce differentiation by relocalizing Egr-1 to the nucleus where it can induce the transcription of genes that encode proteins associated with differentiation.

### Cytosolic PLCβ1 regulates Egr-1/TRBP association

To understand the mechanism that may underlie changes in Egr-1 localization with cytosolic PLCβ1 levels, we considered several factors. First, PLCβ1 can localize in the nucleus of NT2 cells (Fig. 2) and it is possible that this nuclear population is linked to Egr-1 cellular localization. To investigate this idea, we transfected cells with PLCβ1a-M2b and PLCβ1b-M2b constructs that have a reduced nuclear localization as previously reported [37, 40, 54] and shown here (Figs. S8 and 9). However, we found that both mutants were equally competent to induce early transit of Egr-1 to the nucleus (Fig. 5A) suggesting that nuclear localization of PLCβ1 does not impact nuclear localization of Egr-1.

Egr-1 has been found to bind to a population of TRBP (transactivation response element RNA-binding protein) in the cytosol [55]. Previous pull-down and mass spectrometry studies indicate that neither Egr-1 or TRBP are direct or indirect binding partners of PLCβ1 [56]. However, TRBP is a component of Ago2 stress granules that form when cytosolic levels of PLCβ1 are lowered [57]. We tested the idea that cytosolic PLCβ1 regulates the availability of TRBP for Egr-1 binding which may stabilize the cytosolic localization of Egr-1. These studies were done by pulling-down Egr-1 with a TRBP monoclonal antibody in cytosolic extracts of wild type PC12 cells and PC12 cells over-expressing eGFP-PLCβ1. In Fig. 5D, we find that under basal conditions, TRBP complexes with Egr-1 but reduction of PLCβ1 disrupts these interactions.

In a second series of studies, we followed TRBP and Egr-1 colocalization with increased PLCβ1 levels using HEK293 cells that have been engineered to over-express PLCβ1 when treated with tetracycline (see [34]). Even though these cells are not expected to adapt a neuronal phenotype, we find that PLCβ1 over-expression promotes the cells to adapt a neuronal-like morphology. These changes are associated with increased Egr-1 levels and relocalization of both TRBP and Egr-1 into the nucleus (Figs. 5E and S10). These results suggest that cytosolic PLCβ1 regulates Egr-1 localization indirectly through regulation TRBP / Egr-1 interactions in the cytosol (see Discussion).

## Discussion

In this study, we have uncovered a novel pathway through which the signaling enzyme PLCβ1 modulates the differentiation of cultured neuronal cells that may aid in understanding these processes in organisms. This pathway is independent of its plasma membrane lipase activity since it occurs in the absence of agents that stimulate Gα_q_, and occurs under conditions where Gα_q_ expression is low (see [35]). This pathway relies on variations in the level of the cytosolic population of PLCβ1, which has been shown to bind and inhibit the component 3 promoter of RNA-induced silencing complex (i.e., C3PO) [34] and to modulate the aggregation of stress granule proteins [56]. These cytosolic functions of PLCβ1 are distinct from the plasma membrane and nuclear PLCβ1 populations which hydrolyze PIP_2_ in response to environmental stimulation [41].

Of the four known PLCβ isozymes, PLCβ1 is the prevalent form in neuronal tissue, and has been shown to be important in learning, memory and disease (e.g. [58–61]). Using cultured neuronal cells to gain insight into PLCβ1 function, we have found a surge in PLCβ levels during the initial phase of PC12 and SK-N-SH differentiation. In this phase, PLCβ1 localizes in the cytosol and a portion will subsequently move to the plasma membrane as Gα_q_ expression later increases [35]. In this study, we show that increased PLCβ1 expression also occurs when NT2 cells differentiate into neuronal cells either by the antimitotic AraC or the signaling molecule retinoic acid. In PC12 and NT2 cells that take on neuronal phenotypes, this initial increase is followed by a reduction in PLCβ1 levels, while in non-neuronal NT2 cells, PLCβ1 levels remain high. Because this initial PLCβ1 expression is required for differentiation in various cell lines, each with different morphologies and differentiation methods, we conclude that PLCβ1 affects an important shared aspect in the early stages of the differentiation process.

Here, we show that PLCβ1 expression is necessary and sufficient to induce NT2 and PC12 cell differentiation. Over-expression of PLCβ1 in HEK293 cells also induces a shift in morphology. The importance of PLCβ1 in differentiation has been shown in previous and current studies where down-regulating PLCβ1 in undifferentiated cells prevents differentiation, and studies where down-regulating PLCβ1 in differentiated cells returns them to a stem-like state [35, 36]. In primary astrocytes, knocking-down PLCβ1 does not reverse differentiation but results in profound changes in morphology that resembles NT2 neurons, where we find that levels of PLCβ1 are low. Importantly, differentiation appears to only depend on PLCβ1 and not Gα_q_, suggesting that PLCβ1 impacts differentiation, at least in part, by a mechanism independent of its lipase activity. Our observation that we can induce differentiation simply by over-expressing PLCβ1 is surprising and supports the idea that PLCβ1 works through a key factor in differentiation that can be by-passed when expressed at elevated levels.

In trying to understand how PLCβ1 impacts differentiation, we noted that a significant population of the newly synthesized PLCβ1 in PC12 cells and NT2 cells localizes in the cytoplasm [35] suggesting that PLCβ1 is acting through its cytosolic partners. One previously identified partner is C3PO, which promotes RNA-induced silencing [33]. PLCβ1 inhibits C3PO activity and can reverse silencing by siRNAs, and down-regulating PLCβ1 changes the populations of miRs associated with proliferation and differentiation [34, 36]. Thus, it is possible that PLCβ1 over-expression induces differentiation by changing the silencing of gene populations. However, it is unclear whether changes in miR populations could initiate the profound effects seen early in differentiation in these different cell lines and, therefore, we sought to identify the common key factor.

Egr-1 is a short-lived protein that initiates differentiation and its activity can be induced by a variety of different environmental factors [16]. Importantly, Egr-1 is responsible for the initial up-regulation of PLCβ1 [20] as well as its own rise in expression. Therefore, we tested whether PLCβ1 acts through Egr-1. To initiate differentiation, Egr-1 levels increase by both immediate non-coding and subsequent coding mechanisms, and we note that two miRs that regulate Egr-1 levels are connected to PLCβ1 expression (i.e., miR192 and miR146b) [36]. Thus, it is not surprising that we observe a large increase in Egr-1 in NT2 cells immediately after AraC treatment (Fig. S7), and that this increase precedes the increase in PLCβ1 (Fig. 1). It is notable that inducing differentiation by increasing PLCβ1 in both PC12 and NT2 cells reduces the level of Egr-1 consistent with the reduction of Egr-1 levels normally seen towards the end of differentiation [53].

Our studies show that over-expressing PLCβ1 causes a shift of Egr-1 from the cytosol to the nucleus, presumably to induce the transcription of proteins involved in differentiation. We note that we tried to follow these changes in mCherry-Egr-1 in real time, but the transfected cells showed different properties including cell death. Based on our studies, we propose that PLCβ1 induces differentiation, at least in part, by changing the localization rather than the level of Egr-1.

**Fig. 6:**
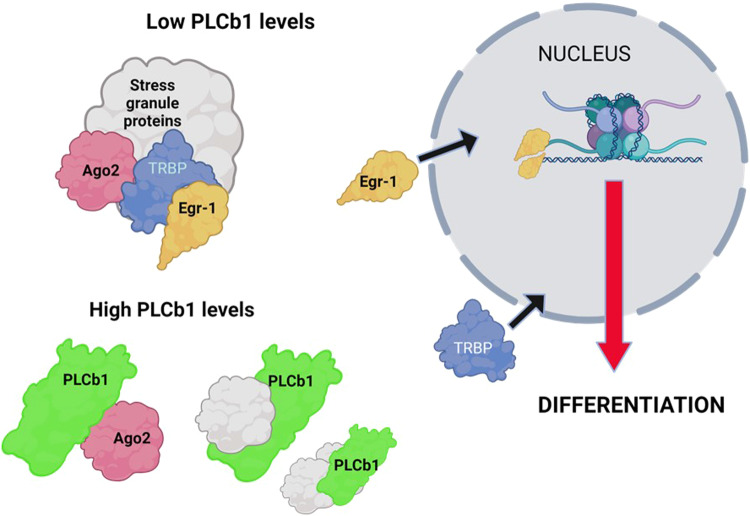
Working model of how PLCβ1 may drive differentiation*.* At low PLCβ1 levels, Ago2 forms complexes that include TRBP [56, 57] which binds Egr-1 in the cytosol [55, 66] stabilizing the cytosolic localization of Egr-1. Increasing PLCβ1 helps solubilize Ago2 complexes releasing TRBP [57] promoting nuclear localization of Egr-1, and potentially TRBP.

We propose a potential mechanism of how PLCβ1 may mediate Egr-1 localization and cell differentiation (Fig. 6). Egr-1 has been shown to bind TRBP in the cytosol [55]. We have found that TRBP is a protein component in Ago2 stress granules that form when cytosolic PLCβ1 is reduced by down-regulation or by activation of Gα_q_ to drive PLCβ1 to the membrane (see [56, 57]). Alternately, high levels of PLCβ1 bind stress granule proteins preventing their aggregation. We propose that when cytosolic PLCβ1 levels are low, such as in the undifferentiated state, TRBP sequesters in Ago2 complexes where it can associate with Egr-1. However, high levels of PLCβ1 help dissolve stress granules allowing Egr-1, and possibly TRBP, to transit to the nucleus and promote the synthesis of proteins required for differentiation including PLCβ1, which in turn further promotes Egr-1 localization to the nucleus. While we believe that Egr-1 re-localization by PLCβ1 is not the only factor driving differentiation, we speculate that it may be a key component.

Control of neuronal cell differentiation by agents based on PLCβ1 here may have impact for treatments for neuroblastomas and diseases associated with neuronal de-differentiation. For example, it has been proposed that the neuronal de-differentiation and subsequent synapse loss plays a role in neurodegenerative diseases such as Alzheimer’s (see [62]). Additionally, neuronal levels of PLCβ1 are negatively associated with the presence and aggressiveness of gliomas [63]. Studies are underway to better identify the role of PLCβ1 in Egr-1 function to develop tools to control differentiation.

## Materials and methods

### Cell culture and differentiation

Human teratocarcinoma NTERA-D1 cells (referred to as NT2 thereinafter) from the American Type Culture Collection (ATCC®, CRL-1973TM) were maintained in complete medium consisting of Dulbecco’s Modified Eagle Medium (DMEM®, ATCC® 30-2002™) supplemented with 10% heat-inactivated fetal bovine serum (FBS, Sigma-Aldrich, St Louis, MO, USA) and antibiotics (100 U/mL penicillin and 100 µg/mL streptomycin) (Gibco, Life Technologies S.A., Madrid Spain) at 37 °C under a humidified atmosphere containing 5% CO_2_. NT2 progenitors were treated as previously described for neuronal differentiation with all-trans retinoic acid (RA, Sigma-Aldrich) [3, 4] and cytosine-β-D-arabinofuranoside (AraC) [6] with slight modifications and improvements for use in our laboratory [3, 64]. Briefly, after 4 weeks of RA treatment, the cells were split at 1:6 ratio. After 2 days, the cells were mechanically dislodged, i.e., the culture flasks were struck 10 times on each side and the floating cells were washed with 5 mL of culture medium and cultured on Matrigel Matrix Basement Membrane (#354234, GIBCO) in medium containing 5% FBS and antimitotics (1 mM cytosine β-D-arabinofuranoside, 10 µM uridine, and 10 µM 5-Fluoro-2’-deoxyuridine) for an additional two weeks. For AraC-induced differentiation, NT2 cultures were treated for 6 days with 20 μM AraC-containing complete medium for 6 days, which was replaced with fresh complete medium supplemented with 20 μM AraC every 2 days. NT2 cells were routinely tested for mycoplasma contamination by PCR using the primers described by Uphoff and Drexler [65]. Positive and negative controls used for these tests were kindly donated by Dr. Uphoff (Leibniz-Institute DSMZ, Department of Human and Animal Cell Lines, Virus Diagnostics, Inhoffenstr. 7b, 38124 Braunschweig, Germany).

Rat PC12 cells (ATCC®, CRL-1721) of no more than 8 passages were grown in complete PC12 medium consisting of DMEM supplemented with 10% heat-inactivated horse serum (Thermo Fisher Scientific), 5% FBS and antibiotics at 37 °C under 5% CO_2_. NGF-induced neuronal was carried out on cells that had reached ~ 80% confluency. Briefly, complete PC12 medium was replaced with PC12 differentiation medium consisting of DMEM supplemented with 1% heat-inactivated horse serum, 0.1 μg/ml recombinant β-NGF (Bio-Techne, 256-GF-100/CF, Minneapolis USA) and antibiotics and incubated for 72 h. Cells treated identically but incubated in P12 differentiation medium without NGF were used as control.

Rat brain hippocampus astrocytes were purchased from Lonza Bioscience (Catalog #: R-HIAS-521) and were cultured at 37 °C, 5% CO_2_ incubator. Culture medium used is the AGM™ Astrocyte Growth Medium BulletKit™ (Lonza Bioscience, catalog #: CC-3186). Astrocytes were placed onto 6 35 mm glass bottom dishes (MatTek P35GC-1.5-14-C) for adherent cell culture and imaging. After culturing ~4 h, the medium was replaced, and the cells cultured for two days.

Human embryonic kidney HEK293-TAP-PLCβ1 cell is a generous gift from Loren Runnels (Rutgers University). This modified cell line was prepared by introducing PLCβ1 tagged with a 38-aa streptavidin-binding peptide on the N-terminus (Sigma, St. Louis, MO, USA) using the Flp-In system (Invitrogen, Carlsbad, CA, USA)^1^. Cells were cultured at 37 °C, 5% CO_2_. in the complete HEK-293 medium consisting of DMEM supplemented with 10% FBS and antibiotics. HEK293-TAP-PLCβ1 cells were evenly split into seven 35 mm glass bottom dishes (MatTek P35GC-1.5-14-C) for adherent cell culture and imaging.

### Molecular cloning

Five double-stranded DNA sequences coding for short-hairpin RNAs (shRNAs), including four encoding siRNAs targeting the human *PLCB1* transcript (shRNA-PLCβ1-87, 89, 97, 99) and one non-target scrambled-base DNA (shRNA-Scrmbl-01) (Table S3) were inserted into the BglII/HindIII cloning site of pSuperior.gfp/neo mammalian expression vector (Oligoengine, #VEC-IND-0007, Seattle, WA, USA) downstream the RNA polymerase III H1-RNA gene promoter.

Cloning of the DNA sequences encoding the human mRNA splice variants of PLCβ1a and PLCβ1b and their corresponding bipartite nuclear localization signal (NLS) mutants PLCβ1a-M2b and PLCβ1b-M2b into the mammalian expression vectors pCDNA3 and pIRES-EGFP-puro (#45567, Addgene; kindly deposited by Prof. Michael McVoy, Virginia Commonwealth University School of Medicine, Richmond, VA) was performed from the commercial plasmids pCMV6-XL6-PLCb1a (#SC126664, OriGene Technologies, Inc, Rockville, USA. USA) and pCMV6-XL6-PLCb1b (#SC309945, OriGene Technologies, Inc.) using molecular biology approaches including conventional PCR, overlap extension PCR and annealing of custom-designed complementary oligonucleotide pairs followed by restriction/ligation-based cloning. Before being subcloned into the multiple cloning site (MCS) of pCDNA3 plasmid, the original inserts containing the open reading frames (ORFs) for PLCβ1a and PLCβ1b in the pCMV6-XL6 vector were modified [i] to remove long fragments corresponding to the 5’ and 3’ UTR regions of the mature mRNAs, [ii] to replace the native translation initiation site with the Kozak consensus sequence (GCC ACC ATG G) and [iii] to add AgeI and NotI unique restriction sites and at the 5’ and 3’ ends, respectively, for subsequent subcloning. In addition, site-directed mutagenesis by PCR was used to reverse a C > T single nucleotide discrepancy (TCA to TTA) detected in commercial plasmids and leading to a S1156L substitution in the protein sequence of human PLCβ1a and PLCβ1b (NCB1 accession, NP_056007.1 and NP_877398.1, respectively). The pcDNA-PLCb1a and pcDNA-PLCb1b constructs thus obtained were used for overlapping PCR mutation of the bipartite nuclear localization signal spanning residues 1055-1071 of both splice variants, thus generating the constructs pcDNA-PLCb1a-M2b and pcDNA-PLCb1b-M2b encoding the PLCβ1a-M2b and PLCβ1b-M2b mutant variants in which three lysine residues of the NLS were replaced by isoleucine (K1056I, K1063I and K1070I), preventing their nuclear localization [37, 40, 54]. Final PLCβ1a, PLCβ1b, PLCβ1a-M2b and PLCβ1b-M2b constructs in pCDNA3 vector were subcloned by restriction/ligation into the MCS of pIRES-EGFP-puro downstream of the cytomegalovirus promoter. Details of molecular cloning are provided in the Supplementary Materials and Methods, Figs. S11–18, Tables S3 and 4 and Additional files 1–4.

### Transfection of plasmids and siRNAs

NT2 cells at ∼70% confluence were transfected with DNA inserts of PLCβ1 constructs into pDCNA3 or in the pIRES-EGFP-puro, which allows the synthesis of two proteins from a single bicistronic mRNA translated through the cap-independent internal ribosome entry site (IRES)-mediated mechanism for the simultaneous and separate expression. Transfection with PLCβ1 shRNAs cloned downstream of the H1 promoter in the pSuperior.gfp/neo plasmid (encoding eGFP under the control of a separate PGK promoter) was performed identically, except that a cocktail prepared by mixing equimolar concentrations four different custom-designed shRNAs targeting different regions of the human PLCβ1 transcript was used. Transfection was carried out with Lipofectamine® 2000 (Invitrogen™) in Opti-MEM™ I Reduced Serum Medium (Thermo Fisher Scientific) without antibiotics according to the manufacturer’s instructions, using a ratio of 2 μl Lipofectamine to 1 μg DNA, using 0.5, 1 and 2.5 µg for 24-, 12- and 6-well plates, respectively. 6 h after transfection, the medium was replaced with complete medium.

PC12 cells at ∼70% were transfected with mouse eGFP-PLCβ1a cloned in pCDNA 2.0 plasmid (a gift from Dr. Catherine Berlot) using Lipofectamine™ 3000 (Invitrogen™) as described for NT2 cells, and 6 h after transfection, the medium was replaced. In all cases, the corresponding empty plasmid (mock) was used as control.

For small interfering (siRNA)-mediated gene knockdown in human NT2 and rat PC12 cells, we used ON-TARGET plus SMART pool siRNAs, which consist of species-specific mixture of four siRNAs targeting different regions of the human (Dharmacon™, Cat. L-010280-00-0010) or rat (Dharmacon™, Cat. L-092936-02-0010) PLCβ1 mRNA. For control experiments, we used ON-TARGETplus Non-targeting Control siRNA Pool (Dharmacon™, Cat. D-001810-10-20), which consists of four small RNAs designed for minimal targeting of human, mouse or rat genes and modified to reduce potential off-targets. Transfection was done on cells at ∼70% using DharmaFECT 1 Transfection Reagent (Dharmacon™, Cat. T-2001-02) in antibiotic and serum-free medium at a ratio of 1 μL DharmaFECT 1 to 2 nmol siRNA, using DharmaFECT 1. The transfection mix was prepared according to the manufacturer’s protocol, using a ratio using a ratio of 2.5 μL of DharmaFECT 1 Reagent for 5 nmol siRNA. 6 h after transfection, the medium was replaced with complete medium and the cells were allowed to grow until harvested or fixed for immunofluorescence staining and microscope analysis.

### Western Blotting

NT2 cells were pelleted under different conditions as indicated in the text and lysed in homogeneization buffer (10 mM Tris-HCl buffer, pH 7.4, 2 mM MgCl_2_, and 0.32 M sucrose) containing protease inhibitors (0.5 mM iodoacetamide and 1 mM phenylmethylsulfonyl fluoride, PMSF). Depending on the antigen to be analyzed, different amounts of denatured lysates (5–30 µg) were loaded, resolved by SDS-polyacrylamide (SDS-PAGE) gels and transferred to polyvinylidene fluoride membranes (PVDF, Bio-Rad, Madrid, Spain). Specific protein bands were detected using conventional inmunoblot protocols. Primary and secondary antibodies are described in Table S1 and Table S2, respectively.

Western blotting to detect proteins in PC12 cells was carried out by lysing cells in ice-cold NP40 buffer (pH = 8.0, 30 ml 5 M NaCl, 100 ml 10% NP40, 50 ml 1 M Tris base, 820 ml diH_2_O) (pH = 8.0, 0.15 M NaCl, 1% NP40, 0.05 M Tris base) for ~10 min at room temperature. To prevent protein degradation, Pierce Protease Inhibitor Mini Tablets were added into NP40 lysis buffer (1 tablet per 10 ml solution, Thermo Fisher Scientific), and the mixture was transferred into 1.5 ml Eppendorf tubes and incubated at 4 °C for 30 min on a shaker. Tubes were centrifuged at 4 °C, 12,000 rpm (c.a. 17,000 RCM, VWR™ Galaxy 14D Digital Microcentrifuges) for 20 min. After centrifugation, supernatant/lysate was transferred into new 1.5 ml Eppendorf tubes. Bradford protein assays were use to estimate protein concentrations of each lysate. Denatured lysates were mixed with loading buffer at 70 °C for 10 min. After denaturation, lysates, SeeBlue™ Plus2 Pre-stained Protein Standard, and MagicMark™ XP Western Protein Standard (Thermo Fisher Scientific) were loaded into 4–15% Mini-PROTEAN® TGX Stain-Free™ Protein 10-well gels (Bio-Rad). Both gel electrophoresis and wet-transfer were performed by employing Bio-Rad’s Mini-PROTEAN Tetra Cell System. Electrophoresis was performed at room temperature, 100 V for around 2 h. After electrophoresis, gels were imaged (Azure Biosystem C600), at 302 nm UV override for 5 min to stimulate trihalo compounds reaction with tryptophan residues to produce fluorescence so that a total protein gel image can be captured for normalization of immunoreactive signal intensities to total protein. After imaging, slow wet-transfer (4 °C, 30 V for 999 min) was performed to transfer proteins from gels to nitrocellulose membranes for blotting. After transfer, membranes were blocked by buffer (TBST buffer with 5% (m/v) nonfat milk powder) for 1 h at 4 °C. For blotting, indirect ECL method was performed for PLCβ1 probing: the primary antibody used is Anti-PLCβ1 Antibody (D-8), and the secondary antibody is m-IgGκ BP-HRP (Santa Cruz Biotechnologies, sc-5291 and sc-516102). EGR1 was probed by employing direct ECL method: the antibody used is Anti-Egr-1 Antibody (B-6) HRP (Santa Cruz Biotechnologies sc-515830 HRP). Anti-PLCβ1 Antibody (D-8) were removed from membranes by incubating membranes with Restore™ PLUS Western Blot Stripping Buffer (Thermo Fisher Scientific 46430) for 10 min at 37 °C after PLCβ1 probing and before EGR1 probing. Data and images were analyzed and plotted on AzureSpot Analysis Software and GraphPad Prism 9 software.

### TRBP immunoprecipitation

PC12 cells were cultured in 20 Nunc™ EasYDish™ Dishes (145 cm^2^, Thermo Fisher Scientific) until around 70% confluence. 10 of the 20 dishes were transfected with eGFP-PLCβ1 plasmid (checked for correct sequence by Plasmidsaurus, Inc.) using Lipofectamine™ 3000 Transfection Reagent (Thermo Fisher Scientific). Cells were incubated with the transfection solution in an antibiotic-free culture medium for c.a. 60 h. Control dishes used the same antibiotic-free culture medium. After incubation, cells were lysed with NP40 lysis buffer (pH = 8.0, 30 ml 5 M NaCl, 100 ml 10% NP40, 50 ml 1 M Tris base, 820 ml diH_2_O), loaded one tablet of Pierce™ Protease Inhibitor Mini Tablets, EDTA-free (Thermo Fisher Scientific) per 10 mL liquid.

For immunoprecipitation, Pierce™ Gentle Ag/Ab Binding Buffer (pH = 8.0, Thermo Fisher Scientific) was used for the wash and binding steps. TRBP Monoclonal Antibody (OTI2C12, Invitrogen™) was combined with Dynabeads™ Protein G (Invitrogen™). Two cell lysates were separately incubated with antibody-conjugated beads on a 3D shaker at room temperature for 1 h to allow TRBP in cell lysates bind with beads. Subsequently, the antigen-antibody complexes were isolated using a magnetic stand and washed thoroughly. Beads were incubated with Pierce™ Gentle Ag/Ab Elution Buffer (pH = 6.6, Thermo Fisher Scientific) at room temperature for 15 min on a 3D shaker and the eluates collected. Spin filters were used to replace the elution buffer with TBS and concentrate the immunoprecipitate. Protein concentrations were determined by bicinchoninic acid assay (BCA) and subjected to gel electrophoresis, followed by transfer to PVDF membranes, immunoblotting with anti-TRBP (OTI2C12, Invitrogen™) antibody, stripping and reprobing with anti-Egr-1 antibody as described above.

Data were analyzed using Fiji-ImageJ (NIH, Bethesda, MA, USA). TRBP bands were selected by same rectangle ROI to determine the gross band mean gray value, and backgrounds of each lane. The net intensity of each TRBP band was the difference of gross band mean gray value and background mean gray value (Eq. 1). For EGR1, bands were similarly selected and analyzed. After obtaining the net intensities of TRBP and EGR1 bands, the relative expression level of EGR1 was calculated by dividing each EGR1 net intensity with their corresponding TRBP net intensity (Eq. 2) for the TRBP band from the same lane. Control samples were used to calculate the normalized fold expression level by dividing relative EGR1 expression level of each data point with the mean value of relative EGR1 expression level of all lanes from control sample (Eq. 3). Normalized fold expression levels of EGR1 in two different treatments (Control and PLCβ1+) were analyzed and plotted by using GraphPad Prism 10 software. Unpaired t test comparison was performed to show the significance of differences: ns (*P* > 0.05), * (*P* ≤ 0.05), ** (*P* ≤ 0.01), *** (*P* ≤ 0.001), and **** (*P* ≤ 0.0001).

1 $${\rm{Net\; Intensity}}={\rm{Gross\; Band\; Mean\; Gray\; Value}}-{\rm{Background\; Mean\; Gray\; Value}}$$

2 $${\rm{Relative\; EGR}}1{\rm{Expression\; Level}}=\,\frac{{\rm{EGR}}1{\rm{Net\; Intensity}}}{{\rm{TRBP\; Net\; Intensity}}}$$

3 $$\begin{array}{l}{\rm{Normalized}}\; {\rm{Fold}}\; {\rm{Expression}}\; {\rm{Level}}\\=\,\displaystyle\frac{{\rm{Relative}}\; {\rm{EGR}}1{\rm{Expression}}\; {\rm{Level}}}{{\rm{mean}}\; {\rm{value}}\; {\rm{of}}\; {\rm{relative}}\; {\rm{EGR}}1{\rm{expression}}\; {\rm{level}}\; {\rm{of}}\; {\rm{all}}\; {\rm{lanes}}\; {\rm{from}}\; {\rm{control}}\; {\rm{sample}}}\end{array}$$

### Immunofluorescence

NT2 cells were fixed in buffered 4% paraformaldehyde for 4 min, both at 20–25 °C. After blocking for 1 h with gelatin-histology buffer (0.1 M PBS, pH 7.4, containing 0.22% gelatin (Panreac, Barcelona, Spain), 0.05% saponin (Sigma-Aldrich), and 1% serum albumin bovine (BSA, Sigma-Aldrich), cells were incubated with primary antibodies (Table S1, for details) at 4 °C overnight. Then, the appropriate fluorescent dye-conjugated secondary antibodies (Table S2 for details) were applied and nuclei were counterstained with Hoechst 33,342 (Sigma-Aldrich, diluted to 0.1 µg/mL in gelatin histology buffer).

PC12 cells from different dishes were transfected with PLCβ1-eGFP plasmid, pcDNA-PLCβ1 plasmid, rat PLCβ1 siRNA, and/or treated with NGF. Controls included cells cultured in antibiotic-free medium, PC12 differentiation medium and complete PC12 medium. After specific times, cells were fixed by 3.7% formaldehyde/DPBS solution (Sigma Aldrich or Thermo Fisher Scientific) for 10 min at room temperature. Formaldehyde was discarded, and cell dishes were rinsed by DPBS several times. Fixed cells were rinsed by MSM pipes buffer (0.4% (v/v) Triton X-100 in DPBS, Sigma Aldrich or Thermo Fisher Scientific) 3 times, 10 min each, and then incubated with blocking buffer (5% (v/v) goat serum, 1% (m/v) BSA, and 50 mM glycine) for 30 min at room temperature to prevent unspecific binding. Cells were then stained by DAPI (Roche Life Science) as per manufacturer’s instructions. For PLCβ1-EGR1 co-localization analysis, cells transfected with rat PLCβ1 siRNA or induced by NGF, and control cells were immunostained with anti-PLCβ1 (1:50 dilution, Santa Cruz Biotechnologies, sc-5291) and then Alexa Fluor™ Plus 488-conjugated goat anti-mouse secondary antibody (1:200 dilution, Thermo Fisher Scientific, # A32723). After immunostaining, cell were rinsed with DPBS several times to completely remove unbound antibodies and prevent cross-staining. Then, cells were immunostained with Alexa Fluor® 647-conjugated anti-Egr-1 antibody (1:50 dilution, Santa Cruz Biotechnologies, sc-515830 AF647). Unbound antibodies were washed away by DPBS. For ERK 1/2 localization study, cells transfected by pcDNA PLCβ1 or rat PLCβ1 siRNA, and control cells were immunostained with anti-ERK 1/2 Antibody (1:200 dilution, Santa Cruz Biotechnologies, sc-292838) and then Alexa Fluor™ 488-conjugated chicken anti-rabbit secondary antibody (1:400 dilution, Thermo Fisher Scientific, # A-21441).

Cells were imaged on a Nikon Eclipse TI-FLBW-E using 60× objective and NIS-Elements AR software. Images were collected at DAPI, FITC (for eGFP and Alexa 488) and TRITC (for Alexa 647) channels for every captured cell. Cell images were analyzed on ImageJ. The freehand selection tool was used to circle cells and their nuclei. Each cell was circled on the merged images, and their nucleus were circled in blue channel images. The mean gray values in every cell and nucleus were measured in red channel images and green channel images. Nuclear Intensity/Cellular Intensity values were calculated by using Eq. (4) to quantify the nuclear aggregations of target proteins in cells which were treated with different conditions. Data from images were analyzed and plotted on GraphPad Prism 9 software.****

4 $${{\rm{Nuclear}}\; {\rm{Intensity}}/{\rm{cellular}}\; {\rm{Intensity}}=\frac{{\rm{Mean}}\; {\rm{Gray}}\; {\rm{Value}}\; {\rm{of}}\; {\rm{Target}}\; {\rm{Protein}}\; {\rm{in}}\; {\rm{Nuclei}}}{{{{\rm{Mean}}\; {\rm{Gray}}\; {\rm{Value}}\; {\rm{of}}\; {\rm{Target}}\; {\rm{Protein}}\; {\rm{in}}\; {\rm{Cell}}}}}}$$

### Statistical analysis

Results were statistically analyzed in GraphPad Prism (version 5.0, GraphPad Software Inc., San Diego, CA) and analyzed using the packages as indicated in the figure legends. Samples sizes were chosen based on the type of experiment and are given in the figure legends.

### Supplementary information

Supplemental material

## Data Availability

All primary data will be freely available upon request to sfscarlata@wpi.edu.

## References

[1] Guroff, G. PC12 Cells as a model of neuronal differentiation in cell culture in the neurosciences. Bottenstein JE, Sato G, editors. Springer US, Boston, MA; 1985. p. 245–72.

[2] Lee VM, Andrews PW (1986). Differentiation of NTERA-2 clonal human embryonal carcinoma cells into neurons involves the induction of all three neurofilament proteins. J Neurosci.

[3] González-Burguera I, Ricobaraza A, Aretxabala X, Barrondo S, García del Caño G, López de Jesús M (2016). Highly efficient generation of glutamatergic/cholinergic NT2-derived postmitotic human neurons by short-term treatment with the nucleoside analogue cytosine β-d-arabinofuranoside. Stem Cell Res.

[4] Pleasure SJ, Lee VM-Y (1993). NTera 2 Cells: A human cell line which displays characteristics expected of a human committed neuronal progenitor cell. J Neurosci Res.

[5] Marchal-Victorion S, Deleyrolle L, De Weille J, Saunier M, Dromard C, Sandillon F (2003). The human NTERA2 neural cell line generates neurons on growth under neural stem cell conditions and exhibits characteristics of radial glial cells. Mol Cell Neurosci.

[6] Musch T, Öz Y, Lyko F, Breiling A (2010). Nucleoside drugs induce cellular differentiation by caspase-dependent degradation of stem cell factors. PLoS ONE.

[7] Trojanowski JQ, Mantione JR, Lee JH, Seid DP, You T, Inge LJ (1993). Neurons derived from a human teratocarcinoma cell line establish molecular and structural polarity following transplantation into the rodent brain. Exp Neurol.

[8] Lee VMY, Hartley RS, Trojanowski JQ. Neurobiology of human neurons (NT2N) grafted into mouse spinal cord: Implications for improving therapy of spinal cord injury. In: Progress in Brain Research, Elsevier; 2000. p. 299–307. 10.1016/S0079-6123(00)28027-8 11105689

[9] Borlongan CV, Tajima Y, Trojanowski JQ, Lee VMY, Sanberg PR (1998). Transplantation of cryopreserved human embryonal carcinoma-derived neurons (NT2N Cells) promotes functional recovery in ischemic rats. Exp Neurol.

[10] Hurlbert MS, Gianani RI, Hutt C, Freed CR, Kaddis FG (1999). Neural transplantation of hNT neurons for Huntington’s disease. Cell Transpl.

[11] Pleasure SJ, Page C, Lee VM (1992). Pure, postmitotic, polarized human neurons derived from NTera 2 cells provide a system for expressing exogenous proteins in terminally differentiated neurons. J Neurosci.

[12] Nelson PT, Kondziolka D, Wechsler L, Goldstein S, Gebel J, DeCesare S (2002). Clonal human (hNT) neuron grafts for stroke therapy: neuropathology in a patient 27 months after implantation. Am J Pathol.

[13] Banerjee S, Williamson D, Habib N, Gordon M, Chataway J (2010). Human stem cell therapy in ischaemic stroke: a review. Age Ageing.

[14] Newman MB, Misiuta I, Willing AE, Zigova T, Karl RC, Borlongan CV (2005). Tumorigenicity issues of embryonic carcinoma-derived stem cells: relevance to surgical trials using NT2 and hNT neural cells. Stem Cells Dev.

[15] Hara K, Matsukawa N, Yasuhara T, Xu L, Yu G, Maki M (2007). Transplantation of post-mitotic human neuroteratocarcinoma-overexpressing Nurr1 cells provides therapeutic benefits in experimental stroke: in vitro evidence of expedited neuronal differentiation and GDNF secretion. J Neurosci Res.

[16] Duclot F, Kabbaj M (2017). The role of early growth response 1 (EGR1) in brain plasticity and neuropsychiatric disorders. Front Behav Neurosci.

[17] Gregg J, Fraizer G (2011). Transcriptional regulation of EGR1 by EGF and the ERK signaling pathway in prostate cancer cells. Genes Cancer.

[18] Liu C, Calogero A, Ragona G, Adamson E, Mercola D (1996). EGR-1, the reluctant suppression factor: EGR-1 is known to function in the regulation of growth, differentiation, and also has significant tumor suppressor activity and a mechanism involving the induction of TGF-beta1 is postulated to account for this suppressor activity. Crit Rev Oncog.

[19] Baek J, Lopez PA, Lee S, Kim TS, Kumar S, Schaffer DV (2022). Egr1 is a 3D matrix-specific mediator of mechanosensitive stem cell lineage commitment. Sci Adv.

[20] Klenke S, Rump K, Buschkamp K, Engler A, Peters J, Siffert W (2014). Characterization of the PLCB1 promoter and regulation by early growth response transcription factor EGR-1. Eur J Pharm.

[21] Garwain O, Valla K, Scarlata S (2018). Phospholipase Cβ1 regulates proliferation of neuronal cells. FASEB J.

[22] Rebecchi MJ, Pentyala SN (2000). Structure, function, and control of phosphoinositide-specific phospholipase C. Physiol Rev.

[23] Suh P, Park J, Manzoli L, Cocco L, Peak J, Katan M (2008). Multiple roles of phosphoinositide-specific phospholipase C isozymes. BMB Rep.

[24] Cocco L, Follo MY, Manzoli L, Suh PG (2015). Phosphoinositide-specific phospholipase C in health and disease. J Lipid Res.

[25] Rusciano I, Marvi MV, Owusu Obeng E, Mongiorgi S, Ramazzotti G, Follo MY (2021). Location-dependent role of phospholipase C signaling in the brain: physiology and pathology. Adv Biol Regul.

[26] Owusu Obeng E, Rusciano I, Marvi MV, Fazio A, Ratti S, Follo MY (2020). Phosphoinositide-dependent signaling in cancer: a focus on phospholipase C isozymes. Int J Mol Sci.

[27] Kamato D, Thach L, Bernard R, Chan V, Zheng W, Kaur H (2015). Structure, function, pharmacology, and therapeutic potential of the G protein, Gα/q,11. Front Cardiovasc Med.

[28] Watanabe M, Nakamura M, Sato K, Kano M, Simon MI, Inoue Y (1998). Patterns of expression for the mRNA corresponding to the four isoforms of phospholipase Cβ in mouse brain. Eur J Neurosci.

[29] Montaña M, García del Caño G, López de Jesús M, González-Burguera I, Echeazarra L, Barrondo S (2012). Cellular neurochemical characterization and subcellular localization of phospholipase C β1 in rat brain. Neuroscience.

[30] Bahk YY, Lee YH, Lee TG, Seo J, Ryu SH, Suh PG (1994). Two forms of phospholipase C-beta 1 generated by alternative splicing. J Biol Chem.

[31] Grubb DR, Vasilevski O, Huynh H, andWoodcock EA (2008). The extreme C-terminal region of phospholipase Cβ1 determines subcellular localization and function; the “b” splice variant mediates α1-adrenergic receptor responses in cardiomyocytes. FASEB J.

[32] Dowal L, Provitera P, andScarlata S (2006). Stable association between G alpha(q) and phospholipase C beta 1 in living cells. J Biol Chem.

[33] Aisiku OR, Runnels LW, Scarlata S (2010). Identification of a novel binding partner of phospholipase Cβ1: translin-associated factor X. PLoS ONE.

[34] Philip F, Guo Y, Aisiku O, Scarlata S (2012). Phospholipase Cβ1 is linked to RNA interference of specific genes through translin-associated factor X. FASEB J.

[35] Garwain O, Scarlata S (2016). Phospholipase Cβ-TRAX association is required for PC12 cell differentiation. J Biol Chem.

[36] Garwain O, Yerramilli VS, Romero K, Scarlata S (2020). The Gαq/phospholipase Cβ signaling system represses tau aggregation. Cell Signal.

[37] Faenza I, Matteucci A, Manzoli L, Billi AM, Aluigi M, Peruzzi D (2000). A role for nuclear phospholipase Cβ1 in cell cycle control *. J Biol Chem.

[38] García del Caño G, Montaña M, Aretxabala X, González-Burguera I, López de Jesús M, Barrondo S (2014). Nuclear phospholipase C-β1 and diacylglycerol LIPASE-α in brain cortical neurons. Adv Biol Regul.

[39] Cocco L, Faenza I, Follo MY, Ramazzotti G, Gaboardi GC, Billi AM (2008). Inositide signaling: Nuclear targets and involvement in myelodysplastic syndromes. Adv Enzym Regul.

[40] Faenza I, Bavelloni A, Fiume R, Lattanzi G, Maraldi NM, Gilmour RS (2003). Up-regulation of nuclear PLCβ1 in myogenic differentiation. J Cell Physiol.

[41] Ramazzotti G, Faenza I, Follo MY, Fiume R, Piazzi M, Giardino R (2011). Nuclear phospholipase C in biological control and cancer. Crit Rev Eukaryot Gene Expr.

[42] Ratti S, Follo MY, Ramazzotti G, Faenza I, Fiume R, Suh P-G (2019). Nuclear phospholipase C isoenzyme imbalance leads to pathologies in brain, hematologic, neuromuscular, and fertility disorders[S]. J Lipid Res.

[43] Novak JE, Agranoff BW, andFisher SK (2000). Increased Expression of Gαq/11 and of Phospholipase-Cβ1/4 in Differentiated Human NT2-N Neurons. J Neurochem.

[44] Zhou J, Valletta JS, Grimes ML, Mobley WC (1995). Multiple levels for regulation of TrkA in PC12 cells by nerve growth factor. J Neurochem.

[45] Garwain O, Pearce KM, Jackson L, Carley S, Rosati B, Scarlata S (2020). Stimulation of the Gαq/phospholipase Cβ1 signaling pathway returns differentiated cells to a stem-like state. FASEB J.

[46] Spires TL, Molnár Z, Kind PC, Cordery PM, Upton AL, Blakemore C (2005). Activity-dependent regulation of synapse and dendritic spine morphology in developing barrel cortex requires phospholipase C-beta1 signalling. Cereb Cortex.

[47] Faenza I, Bregoli L, Ramazzotti G, Gaboardi G, Follo MY, Mongiorgi S (2008). Nuclear phospholipase C beta1 and cellular differentiation. Front Biosci.

[48] Janesick A, Wu SC, Blumberg B (2015). Retinoic acid signaling and neuronal differentiation. Cell Mol Life Sci.

[49] Masiá S, Alvarez S, de Lera AR, Barettino D (2007). Rapid, nongenomic actions of retinoic acid on phosphatidylinositol-3-kinase signaling pathway mediated by the retinoic acid receptor. Mol Endocrinol.

[50] Qiao J, Paul P, Lee S, Qiao L, Josifi E, Tiao JR (2012). PI3K/AKT and ERK regulate retinoic acid-induced neuroblastoma cellular differentiation. Biochem Biophys Res Commun.

[51] Grubb DR, Vasilevski O, Huynh H, andWoodcock EA (2008). The extreme C-terminal region of phospholipase Cbeta1 determines subcellular localization and function; the “b” splice variant mediates alpha1-adrenergic receptor responses in cardiomyocytes. FASEB J.

[52] Maik-Rachline G, Hacohen-Lev-Ran A, Seger R (2019). Nuclear ERK: mechanism of translocation, substrates, and role in cancer. Int J Mol Sci.

[53] Adams KW, Kletsov S, Lamm RJ, Elman JS, Mullenbrock S, Cooper GM (2017). Role for Egr1 in the transcriptional program associated with neuronal differentiation of PC12 Cells. PLoS ONE.

[54] Kim CG, Park D, Rhee SG (1996). The role of carboxyl-terminal basic amino acids in Gqα-dependent activation, particulate association, and nuclear localization of phospholipase C-β1. J Biol Chem.

[55] Wei J, Ouyang Y, Li X, Zhu B, Yang J, Cui Y (2015). Early growth response gene 1, a TRBP binding protein, is involved in miRNA activity of miR-125a-3p in human cells. Cell Signal.

[56] Qifti A, Jackson L, Singla A, Garwain O, Scarlata S (2021). Stimulation of phospholipase Cbeta1 by Galphaq promotes the assembly of stress granule proteins. Sci Signal.

[57] Jackson L, Rennie M, Poussaint A, Scarlata S (2022). Activation of Gαq sequesters specific transcripts into Ago2 particles. Sci Rep.

[58] Ruiz de Azúa I, del Olmo E, Pazos A, Sallés J (2006). Transmembrane signaling through phospholipase C-beta in the developing human prefrontal cortex. J Neurosci Res.

[59] McOmish CE, Burrows E, Howard M, Scarr E, Kim D, Shin HS (2008). Phospholipase C-beta1 knockout mice exhibit endophenotypes modeling schizophrenia which are rescued by environmental enrichment and clozapine administration. Mol Psychiatry.

[60] Kim HJ, Koh HY (2016). Impaired reality testing in mice lacking phospholipase Cβ1: observed by persistent representation-mediated taste aversion. PLoS ONE.

[61] Udawela M, Scarr E, Boer S, Um JY, Hannan AJ, McOmish C (2017). Isoform specific differences in phospholipase C beta 1 expression in the prefrontal cortex in schizophrenia and suicide. npj Schizophrenia.

[62] Arendt T (2000). Alzheimer’s disease as a loss of differentiation control in a subset of neurons that retain immature features in the adult brain. Neurobiol Aging.

[63] Bayeva N, Coll E, Piskareva O (2021). Differentiating neuroblastoma: a systematic review of the retinoic acid, its derivatives, and synergistic interactions. J Pers Med.

[64] González-Burguera I, Ricobaraza A, Aretxabala X, Barrondo S, García del Caño G, López de Jesús M (2016). Data for the morphometric characterization of NT2-derived postmitotic neurons. Data Brief.

[65] Uphoff CC, Drexler HG (2014). Detection of mycoplasma contamination in cell cultures. Curr Protoc Mol Biol.

[66] Silverman ES, Du J, Williams AJ, Wadgaonkar R, Drazen JM, Collins T (1998). cAMP-response-element-binding-protein-binding protein (CBP) and p300 are transcriptional co-activators of early growth response factor-1 (Egr-1). Biochem J.

