# Supplementary material for: PLCβ1 by-passes early growth response -1 to induce the differentiation of neuronal cells
Source: Cell Death Discov. 2024 May 24;10:250. doi: 10.1038/s41420-024-02009-z (PMC11126630; doi:10.1038/s41420-024-02009-z)

Supplemental Methods

Molecular cloning

For cloning of short-hairpin RNAs (shRNAs) into the pSuperior.gfp/neo expression vector (Oligoengine Corporate, #VEC-IND-0007, Seattle, WA, USA) five oligonucleotide pairs were designed with Oligoengine software version 2.0 (Oligoengine, Seattle, WA, USA) to generate 5 inserts, all containing a 19-nucleotide (19-nt) reverted repeat separated by a 9-nt spacer sequence. Four of the five inserts (shRNA-PLCb1-87, 89, 97, 99) were designed with a repeat antisense sequence targeting the human *PLCB1* transcript and an additional one with a non-targeting (scrambled) nucleotide sequence (shRNA-Scrmb-01) (Table S3). To generate double-stranded shRNA inserts, chemically synthesized oligonucleotides were annealed using a Bio-Rad thermocycler (Bio-Rad, MyCycler™ thermocycler system). Complementary oligonucleotide pairs, both at a final 2 μM concentration, were hybridized in 50 μL annealing buffer (100 mM NaCl, 50 mM HEPES, pH 7.4). The mixture of oligonucleotide pairs was heated at 90 °C for 4 min, then cooled to 70 °C for 10 min, and subsequently cooled in a stepwise manner to 37 °C for 20 min, followed by cooling to 10 °C before being used immediately or stored at -20 °C until use.

The double-stranded DNA thus generated with the forward and reverse oligonucleotides possessed 5' BamHI and 3' HindIII overhangs, thus allowing cloning into the BglII (BamHI-compatible) and HindIII unique sites of the multiple cloning site (MCS) of pSuperior.gfp/neo using T4 DNA Ligase (#2011A, Takara Bio Inc., Shiga, Japan), which resulted in the destruction of the BamHI site to facilitate the screening of positive clones.

Commercial plasmids pCMV6-XL6-PLCb1a (#SC126664, OriGene Technologies, Inc, Rockville, USA. USA) and pCMV6-XL6-PLCb1a (#SC309945, OriGene Technologies, Inc.) containing the open reading frames (ORFs) for human PLCβ1a and PLCβ1b splice variants were used for sublconing into the pCDNA3 and bicistronic pIRES-EGFP-puro (Addgene, #45567, Cambridge, MA, USA) mammalian expression plasmids. First, using pCMV6-XL6-PLCb1a as template for PCR amplification using PLCb1_AgeI_Fw and PLCb1a_NotI_Rv primers (Table S4). Briefly, 2 μL template (1 μg/μL) 10-, 100- or 1000-fold diluted were brought to a final volume of 50 μL reaction mixture containing 2 mM Mg^{2+}, 0.3 mM dNTPs, 0.3 μM primer mix and 1.5 units KAPA HiFi Taq polymerase (#KK2101; KAPA Biosystems, Inc., Woburn, MA, USA). PCR reactions were performed using a Bio-Rad thermocycler (Bio-Rad, MyCycler™ thermocycler

system). Following a hot start for 5 min at 95 °C, amplification was performed by 27 PCR cycles of melting at 94 °C for 1 min, annealing at 59 °C for 30 sec and extending at 68 °C for 1 min. After a final extension step at 72 °C for 5 min, samples were cooled to 4 °C and the PCR products checked for purity and length in minigels, followed by migration in preparative agarose gels and purification using Macherey-Nagel Nucleospin® Gel and PCR Clean-Up columns (#740609; Clontech, Madrid, Spain) according to the manufacturer's instructions. A single amplicon with a size consistent with the expected one of 3675 bp was obtained at the 1:100 matrix dilution as determined by agarose gel electrophoresis (Fig. S11A). The PCR product was then purified by preparative agarose gel electrophoresis and purified using Macherey-Nagel Nucleospin® Gel and PCR Clean-Up columns (#740609; Clontech, Madrid, Spain) according to the manufacturer's instructions. The purified fragment was then inserted into the pCR-Blunt II-TOPO™ cloning plasmid and one Shot® TOP10 chemically competent bacteria (#C404010; Thermo Fisher Scientific) were heat shock transformed with the PCR product-containing plasmids and cultured in on LB agar (Lennox) (#1083; Condalab, Madrid, Spain) supplemented with 50 µg/mL kanamycin (#SR0092; Thermo Fisher Scientific). The plasmid DNA was then purified with Macherey-Nagel Nucleospin® Plasmid columns (#740588; Clontech) and digested for screening with EcoRV (#R0195S; New England Biolabs, Barcelona, Spain) and BglII (#R0144S; New England Biolabs). Positive clones were selected for sequencing at the STABVIDA sequencing facility (Lisbon, Portugal). For cloning into the mammalian pCDNA3, insert-containing TOPO plasmids and pCDNA3 vector were digested with AgeI (#R0552S; New England Biolabs) and NotI (#R0189L; New England Biolabs) restriction enzymes. Inserts were then ligated into the linearized pCDNA3 plasmid with T4 DNA ligase (#2011A; Takara Bio Inc., Shiga, Japan) for 18 hours at 16 °C with a vector to insert molar ratios of 1:10. TOP10 chemically competent bacteria were transformed with the ligation products and cultured in LB-agar supplemented with 100 µg/mL of ampicillin (#A9518; Sigma- Aldrich). For colony screening we proceeded as described for the pCR™-Blunt II-TOPO™ plasmid, except that LB Broth medium contained ampicillin (100 µg/mL) instead of kanamycin. The positive clones were sent for sequencing to verify the position of the inserts in the correct reading frame and the absence of mutations that alter the expected amino acid coding (Fig. S11B). As a result, we obtained a N-terminal pCDNA-PLCb1a cloning vector (pCDNA-PLCb1a-N) lacking the endogenous Kozak and possessing a unique restriction AgeI site close to the 5' end of the ORF to allow the insertion of DNA duplexes containing a Kozak

consensus sequence (GCC ACC ATG G) and, eventually, any desired N-terminal tag in frame with the ATG initiation codon.

To reconstruct a functional pCDNA3 expression vector coding for human PLCβ1a (pCDNA-PLCb1a), the pCDNA-PLCb1a-N vector was digested with KpnI (#R0142S; New England Biolabs) and AgeI enzymes and the restriction fragment was replaced by T4 DNA ligation with a duplex DNA with cohesive KpnI and cohesive/destructive AgeI overhangs and containing a Kozak/start consensus sequence (GCC ACC ATG G) together with unique EcoRV add AgeI sites for further subcloning (Fig. S12A). After screening by restriction with EcoRI (#R0101S; New England Biolabs, Barcelona, Spain) and MluI (#R0198S; New England Biolabs), positive clones were selected for sequencing (Fig. S12B). The resulting mammalian expression plasmid pCNDA-PLCb1a, encoding the human PLCβ1a protein, lacked the long fragments corresponding to the 5' and 3' UTR region present in the commercial pCMV6-XL6-PLCb1a plasmid, had a Kozak/start consensus sequence (GCC ACC ATG G) for more efficient translation (Fig. S12A) and unique AgeI and NotI restriction sites at the 5' and 3' ends, respectively, for further subcloning.. The plasmids pCDNA-PLCb1a and the commercial pCMV6-XL6-PLCb1b were used to generate a pCDNA-PLCb1b vector with similar characteristics to pCDNA-PLCb1a by restriction/ligation. To this end, the pCDNA-PLCb1a and pCMV6-XL6-PLCb1b vectors were digested with the restriction enzymes PmlI (#R0532S; New England Biolabs) and NotI. The resulting large 7225-bp fragment of the pCDNA-PLCb1a plasmid, containing the open reading frame common to the PLCb1a and PLCb1b splicing variants, was ligated with a smaller 1726-bp fragment of pCMV6-XL6-PLCB1b, containing the 3' end specific to PLCb1b. TOP10 chemically competent bacteria were transformed with the ligation products as above, cultured in LB-agar with of ampicillin, followed by colony screening with EcoRV and BamHI enzyme (#R0136S, New England Biolabs) (Fig. S13).

To reverse a C>T single nucleotide discrepancy (TCA to TTA) detected in commercial plasmids and leading to a S1156L substitution in the protein sequence of human PLCβ1a and PLCβ1b (NCB1 accession, NP_056007.1 and NP_877398.1, respectively) we designed a site-directed mutagenesis strategy based on overlap extension PCR. To this end a first couple of PCRs was performed. One PCR on pCDNA-PLCb1a plasmid with a forward primer located about 1,500 bp upstream of the point mutation and containing the unique PmlI restriction site within the ORFs of both pCDNA-PLCb1a and pCDNA-PLCb1b (PLCb1-PmlI-Fw) and a reverse primer

encompassing the point mutation (T/C-Rv). The other PCR was done on both pCDNA-PLCb1a and pCDNA-PLCb1b plasmids with a forward primer (T/C-Fw) consisting of the reverse complement of T/C-Rv and a reverse primer (pCDNA-XmaI-Rv) located within the pCDNA plasmid about 1,500 bp downstream of the point mutation and 25 bp downstream the unique XmaI restriction site (Table S4 for primer sequences). After a hot start for 5 min at 94 °C, amplification was performed by 27 PCR cycles of melting at 94 °C for 1 min, annealing at 62 °C for 30 sec and extending at 70 °C for 1 min. Following a final extension step at 72 °C for 5 min, three PCR products were obtained, one resulting from the PCR on pCDNA-PLCb1a using primer pairs PLCb1-PmlI-Fw and T/C-Rv (PLCb1-T/C-Fw amplicon), and two resulting from the PCR on pCDNA-PLCb1a and pCDNA-PLCb1b using primer pairs T/C-Rv and pCDNA-XmaI-Rv (PLCb1a-T/C-Rv and PLCb1b-T/C-Rv amplicons, respectively) (Fig. S14A). Purified PLCb1-PmlI-Fw was then combined with either PLCb1a-T/C-Rv or PLCb1b-T/C-Rv (2 µL each) and hot-start PCR was run by 5 cycles of melting at 96 °C for 1 min, annealing at 60 °C for 30 sec and extending at 70 °C for 1 min, followed by 5 min extension at 72 °C. Finally, PLCb1-PmlI-Fw and pCDNA-XmaI-Rv primers were added (0.3 µM both) and 25 PCR cycles were run with melting at 96 °C for 1 min, annealing at 63 °C for 30 sec, extension at 70 °C for 2 min and final extension at 72 °C for 5 min, thus obtaining PLCb1a-T/C and PLCb1b-T/C fragments (Fig. S14B). After purification, insertion in pCR™-Blunt II-TOPO™ plasmid for sequencing, PLCb1a-T/C and PLCb1b-T/C fragments were used to replace the corresponding fragments containing the C>T single nucleotide discrepancy by restriction ligation using PmlI and XmaI (#R0180L; New England Biolabs) and T4 ligase enzymes. The sequences of the final pCDNA-PLCb1a and pDCNA-PLCb1b constructs are provided in Additional text files 1 and 2.

PLCβ1a-M2b and PLCβ1b-M2b mutants of the bipartite nuclear localization signal (NLS) was performed by PCR overlap extension to replace the native NLS (KKLKEICEKEKKELKKKM) spanning residues 1055-1071 of both PLCβ1a and PLCβ1b with the mutant M2b sequence KILKEICEIEKKELKIK (K1056I, K1063I and K1070I). For this purpose, we designed partially overlapping M2b-Rv and M2b-Fw (Table S4) oligonucleotides carrying a total of 4 nucleotide mutations modifying the coding codons of lysines 1056 (AAG to ATA), 1063 (AAA to ATA) and 1070 (AAA to ATA) to isoleucine (Fig. S15). The procedure used was similar to that described above to reverse the C>T single nucleotide discrepancy, with some modifications to the PCR amplification conditions. Briefly, three PCR reactions were carried out

with PLCb1-PmlI-Fw and M2b-Rv primers on pCDNA-PLCb1a and with M2b-Fw and PLCb1-XmaI-Rv (hot start 94 °C, 5 min followed by 30 PCR cycles with melting 96 °C for 1 min, annealing 63 °C for 30 sec , extension 72 °C for 2 min, final extension 72 °C for 5 min), thus obtaining PLCb1-M2b-Fw, PLCb1a-M2b-Rv and PLCb1b-M2b-Rv (Fig. S16A) for PCR assembly by a two-step PCR. First step: hot-start, followed by 5 PCR cycles of melting at 96 °C for 1 min, annealing at 56-64 °C gradient for 30 sec and extending at 72°C for 1 min, followed by 5 min extension at 72 °C. In the second step, PLCb1-PmlI-Fw and pCDNA-XmaI-Rv primers were added (0.3 µM both) and 30 PCR cycles were run with melting at 96 °C for 1 min, annealing at 63 °C for 30 sec, extension at 70 °C for 2 min and final extension at 72 °C for 5 min. The resulting PLCb1a-M2b and PLCb1b-M2b fragments (Fig. S16B) were used to generate pCDNA-PLCb1a-M2b and pCDNA-PLCb1b-M2b plasmids by restriction ligation using PmlI and XmaI and T4 ligase enzymes as described above (sequences in additional files 3 and 4).

Subcloning of PLCb1a, PLCb1b, PLCb1a-M2b and PLCb1b-M2b in the multiple cloning site (MCS) of pIRES-EGFP-puro (#45567, Addgene; kindly deposited by Prof. Michael McVoy, Virginia Commonwealth University School of Medicine, Richmond, VA), downstream of the cytomegalovirus promoter, was performed by restriction/ligation. First, to obtain cohesive fragments containing the complete ORF corresponding for the human PLCβ1b, and the corresponding NLS mutant PLCβ1b-M2b, pCDNA-PLCb1b and pCDNA-PLCb1b-M2b plasmids were blunt digested a few base pairs upstream from the Kozak/start site with EcoRV and 38 bp downstream the stop codon with XhoI (#R0146S; New England Biolabs). pIRES-EGFP-puro was linearized by combining blunt digestion of AfeI (#R0652S; New England Biolabs) and overhang digestion of XhoI unique sites within the MCS of the bicistronic pIRES-EGFP-puro vector. The obtained insert fragments containing the coding sequences for PLCb1b and PLCb1b-M2b were then inserted into the linearized pIRES-EGFP-puro plasmid with T4 DNA ligase to obtain pIRES-PLCb1b-EGFP (Fig. S17A) and pIRES-PLCb1b-M2b-EGFP constructs (Fig. S17B). The pIRES-PLCb1b-EGFP plasmid was then used to generate pIRES-PLCb1a-EGFP and pIRES-PLCb1a-M2b-EGFP constructs by restriction/ligation with AgeI and NotI restriction enzymes. For this purpose, the complete PLCb1b ORF in pIRES-PLCb1b-EGFP was replaced with the ORFs corresponding to PLCb1a and PLCb1a-M2b pCDNA-PLCb1a (Fig. S18A) and pCDNA-PLCb1a-M2b (Fig. S18B) using procedures described in detail above.

Supplemental tables

Table S1. Primary antibodies

Target protein	Dilution IF	Dilution WB	Species & clonality	Isotype	Immunizing antigen	Source, Cat. No.
CyPA	-	1:1000	Mouse monoclonal	IgG_{2a}	Recombinant CYPA protein of human origin	Sta Cruz Biotech. Inc., #sc-134310
Egr-1	1:250	1:1000	Mouse monoclonal	IgG_1	Epitope mapping between amino acids 524-543 at the C-terminus of Egr-1 of human origin	Sta Cruz Biotech. Inc., #sc-515830
GFP	1:4000	1:2500	Rabbit polyclonal	IgG	GFP isolated from *Aequorea victoria*	Invitrogen, #A11122
Ki67	1:2000	-	Rabbit polyclonal	IgG	Synthetic peptide within human Ki67 aa 2300-2400. The exact sequence is proprietary	Abcam, #16667
NF200	1:400	1:10000	Rabbit polyclonal	IgG	NF200 protein purified from bovine spinal cord	Sigma-Aldrich, #N4142
PLCβ1	1:125	-	Mouse monoclonal	IgG_{2b}	Synthetic peptide mapping within amino acids 831-1063 of rat PLCβ1	S$^{\underline{ta}}$ Cruz Biotech. Inc., #sc-5291
PLCβ1	-	1:750	Mouse monoclonal	IgG_1	Synthetic peptide corresponding to amino acids 4–159 of the rat PLCβ1	BD Transduction Labs., #610924
PLCβ1a	1:250	1:500	Rabbit polyclonal	IgG	Synthetic peptide corresponding to a unique C-terminal region of PLCβ1a splice variant	S$^{\underline{ta}}$ Cruz Biotech. Inc., #sc-205
PLCβ4	-	1:500	Rabbit polyclonal	IgG	Synthetic peptide mapping at the C-terminus of rat PLCβ4	S$^{\underline{ta}}$ Cruz Biotech. Inc., #sc-404
β-Actin	-	1:40000	Mouse monoclonal	IgG1	An epitope mapping at the N-terminal end of β-actin	Sigma-Aldrich, #A5441
βIII Tubulin	1:1000	1:1000	Chicken polyclonal	Chicken serum	Synthetic peptides corresponding to different regions shared by human and rat β-tubulin III	Abcam, #ab41489

Table S2. Secondary antibodies for immunofluorescence and Western blot

Antibody	Dilution	Use	Source, Cat. No.
Alexa Fluor 488 Goat anti-Rabbit IgG	1:400	Immunofluorescence	Invitrogen, A-11034
Alexa Fluor 568 Goat anti-Mouse IgG	1:400	Immunofluorescence	Invitrogen, A-11031
DyLight 649 Goat anti-Chicken IgY;	1:400	Immunofluorescence	Jackson Immuno Research, 103-495-155
Alexa Fluor 488 Goat anti-Rabbit IgG	1:400	Immunofluorescence	Invitrogen, A-11034.
Alexa Fluor 568 Goat anti-Mouse IgG	1:400	Immunofluorescence	Invitrogen, A-11031.
HRP-conjugated Donkey anti-Rabbit IgG	1:10,000	Immunoblot	Amersham, NA934
HRP-conjugated Sheep anti-Mouse IgG	1:10,000	Immunoblot	Amersham, NXA931
HRP-conjugated Rabbit anti-Chicken IgY	1:10,000	Immunoblot	Sigma-Aldrich, A9046

Table S3. Oligonucleotide pairs (unshaded cells) used to generate double-stranded DNA (shaded cells) encoding shRNAs.

Oligoenglne References and names of DNA inserts	Oligonucleotide Sequences and duplexes
129501 Scramble_HIND<br>129502 Scramble_HIND_As	5' GATCCCCGCGCGCTTTGTAGGATTCGTTCAAGAGACGAATCCTACAAAGCGCGCTTTTTA 3'<br>5' AGCTTAAAAAGCGCGCTTTGTAGGATTCGTCTCTTGAACGAATCCTACAAAGCGCGCGGG 3'
**shRNA-Scrmb-01**	5' GATCCCCGCGCGCTTTGTAGGATTCGTTCAAGAGACGAATCCTACAAAGCGCGCTTTTTA 3'<br>3' GGGCGCGCGAAACATCCTAAGCAAGTTCTCTGCTTAGGATGTTTCGCGCGAAAAATTCGA 5'
129487 NM_015192-1526.HIND<br>129488 NM_015192-1526.HIND_As	5' GATCCCCGGAAAGTGACGACGACGATTTCAAGAGAATCGTCGTCGTCACTTTCCTTTTTA 3'<br>5' AGCTTAAAAAGGAAAGTGACGACGACGATTCTCTTGAAATCGTCGTCGTCACTTTCCGGG 3'
**shRNA-PLCb1-87**	5' GATCCCCGGAAAGTGACGACGACGATTTCAAGAGAATCGTCGTCGTCACTTTCCTTTTTA 3'<br>3' GGGCCTTTCACTGCTGCTGCTAAAGTTCTCTTAGCAGCAGCAGTGAAAGGAAAAATTCGA 5'
129489 NM_015192-3346.HIND<br>129490 NM_015192-3346.HIND_As	5' GATCCCCCGCAAAGTAAACGGCAAGATTCAAGAGATCTTGCCGTTTACTTTGCGTTTTTA 3'<br>5' AGCTTAAAAACGCAAAGTAAACGGCAAGATCTCTTGAATCTTGCCGTTTACTTTGCGGGG 3'
**shRNA-PLCb1-89**	5' GATCCCCCGCAAAGTAAACGGCAAGATTCAAGAGATCTTGCCGTTTACTTTGCGTTTTTA 3'<br>3' GGGGCGTTTCATTTGCCGTTCTAAGTTCTCTAGAACGGCAAATGAAACGCAAAAATTCGA 5'
129497 PLCB1-247.HIND<br>129498 PLCB1-247.HIND_As	5' GATCCCCGGACCCCAAATTACGTGAATTCAAGAGATTCACGTAATTTGGGGTCCTTTTTA 3'<br>5' AGCTTAAAAAGGACCCCAAATTACGTGAATCTCTTGAATTCACGTAATTTGGGGTCCGGG 3'
**shRNA-PLCb1_97**	5' GATCCCCGGACCCCAAATTACGTGAATTCAAGAGATTCACGTAATTTGGGGTCCTTTTTA 3'<br>3' GGGCCTGGGGTTTAATGCACTTAAGTTCTCTAAGTGCATTAAACCCCAGGAAAAATTCGA 5'
129499 PLCB1-751.HIND<br>129500 PLCB1-751.HIND	5' GATCCCCGATTTCACTCCAGAAGTGTTTCAAGAGAACACTTCTGGAGTGAAATCTTTTTA 3'<br>5' AGCTTAAAAAGATTTCACTCCAGAAGTGTTCTCTTGAAACACTTCTGGAGTGAAATCGGG 3'
**shRNA-PLCb1-99**	5' GATCCCCGATTTCACTCCAGAAGTGTTTCAAGAGAACACTTCTGGAGTGAAATCTTTTTA 3'<br>3' GGGCTAAAGTGAGGTCTTCACAAAGTTCTCTTGTGAAGACCTCACTTTAGAAAAATTCGA 5'

Yellow shading, BamHI overhang. **Green shading**, HindIII overhang. **Dark green characters**, 19-nt sense sequence of the DNA repeat. **Red characters**, 19-nt antisense sequence of the DNA repeat. **Blue characters**, 9-nt spacer to allow hairpin formation. **Underlined nucleotide**, transcription start signal 26-nt downstream the TATA box sequence of the H1 promoter (TATAA). **Cyan shading**, poly-deoxythymidine transcription stop signal for RNA polymerase III.

Table S4. List of primers and oligonucleotides

Primer names	Nucleotide sequences (5' > 3')	Use
PLCb1-AgeI-Fw	ACCGGTGTGCACGCCTTGCAAC	1
PLCb1a-NotI-Rv	GCGGCCGCCATGCAATTTCTG	1
KpnI-Kozak-PLCb1-Fw	CGAGCTTCGATCCACCAGATATCCAGTACCGGTGCCACCATGGCCGGGGCTCAAC	1
KpnI-Kozak-PLCb1-Rv	CCGGGTTGAGCCCCGGCCATGGTGGCACCGGTACTGGATATCTGGTGGATCGAAGCTCGGTAC	1
PLCb1-PmlI-Fw	GAACACGTGTGGATTCATCC	2
pCDNA-XmaI-Rv	CCTCATCCTGTCTCTTGATC	2
T/C-Rv	CCTGGATATATGACCGGATC	2, 3
T/C-Fw	GATCCGGTCATATATCCAGG	2, 3
M2b_Rv	TTCTTTCTTTTCTATCTCACAGATTTCTTTGAGTATCTTTAACTGATTGTTCTGAC	3
M2b_Fw	AATCTGTGAGATAGAAAAGAAAGAATTAAAGAAGATAATGGATAAAAAGAGGCAGGAG	3

List of primers used for subcloning the open reading frames (ORFs) for human PLCβ1a and PLCβ1b splice variants into pCDNA3 using commercial pCMV6-XL6-PLCb1a and pCMV6-XL6-PLCb1b (#SC309945, OriGene Technologies, Inc.) vectors as matrices (**1**) reversing a C>T single nucleotide discrepancy (TCA to TTA) detected in commercial plasmids and leading to a S1156L substitution (**2**) and generate mutants of the bipartite nuclear localization signal pCDNA-PLCb1a-M2b and pCDNA-PLCb1b-M2b (**3**).

Additional file 1

```
tggcttatcgaaattaatacgactcactatagggagacccaagcttggtaccgagcttcgatccaccagatatccagtaccggtgccacc      -1

ATGGCCGGGGCTCAACCCGGTGTGCACGCCTTGCAACTCAAGCCCGTGTGCGTGTCCGACAGCCTCAAGAAGGGCACCAAATTCGTCAAG      90
MetAlaGlyAlaGlnProGlyValHisAlaLeuGlnLeuLysProValCysValSerAspSerLeuLysLysGlyThrLysPheValLys      30

TGGGATGATGATTCAACTATTGTTACTCCAATTATTTTGAGGACTGACCCTCAGGGATTTTTCTTTTACTGGACAGATCAAAACAAGGAG     180
TrpAspAspAspSerThrIleValThrProIleIleLeuArgThrAspProGlnGlyPhePhePheTyrTrpThrAspGlnAsnLysGlu      60

ACAGAGCTACTGGATCTCAGCCTTGTCAAAGATGCCAGATGTGGGAGACACGCCAAAGCTCCCAAGGACCCCAAATTACGTGAACTTTTG     270
ThrGluLeuLeuAspLeuSerLeuValLysAspAlaArgCysGlyArgHisAlaLysAlaProLysAspProLysLeuArgGluLeuLeu      90

GATGTGGGGAACATCGGGCGCCTGGAGCAGCGCATGATCACAGTGGTGTATGGGCCTGACCTCGTGAACATCTCCCATTTGAATCTCGTG     360
AspValGlyAsnIleGlyArgLeuGluGlnArgMetIleThrValValTyrGlyProAspLeuValAsnIleSerHisLeuAsnLeuVal     120

GCTTTCCAAGAAGAAGTGGCCAAGGAATGGACAAATGAGGTTTTCAGTTTGGCAACAAACCTGCTGGCCCAAAACATGTCCAGGGATGCA     450
AlaPheGlnGluGluValAlaLysGluTrpThrAsnGluValPheSerLeuAlaThrAsnLeuLeuAlaGlnAsnMetSerArgAspAla     150

TTTCTGGAAAAAGCCTATACTAAACTTAAGCTGCAAGTCACTCCAGAAGGGCGTATTCCTCTCAAAAACATATATCGCTTGTTTTCAGCA     540
PheLeuGluLysAlaTyrThrLysLeuLysLeuGlnValThrProGluGlyArgIleProLeuLysAsnIleTyrArgLeuPheSerAla     180

GATCGGAAGCGAGTTGAAACTGCTTTAGAGGCTTGTAGTCTTCCATCTTCAAGGAATGATTCAATACCTCAAGAAGATTTCACTCCAGAA     630
AspArgLysArgValGluThrAlaLeuGluAlaCysSerLeuProSerSerArgAsnAspSerIleProGlnGluAspPheThrProGlu     210

GTGTACAGAGTTTTCCTCAACAACCTTTGCCCTCGACCTGAAATTGATAACATCTTTTCAGAATTTGGTGCAAAAAGCAAACCATATCTT     720
ValTyrArgValPheLeuAsnAsnLeuCysProArgProGluIleAspAsnIlePheSerGluPheGlyAlaLysSerLysProTyrLeu     240

ACCGTTGATCAGATGATGGATTTTATCAACCTTAAGCAGCGAGATCCTCGGCTTAATGAAATACTTTATCCACCTCTAAAACAAGAGCAA     810
ThrValAspGlnMetMetAspPheIleAsnLeuLysGlnArgAspProArgLeuAsnGluIleLeuTyrProProLeuLysGlnGluGln     270

GTCCAAGTATTGATTGAGAAGTATGAACCCAACAACAGCCTCGCCAGAAAAGGACAAATATCAGTGGATGGGTTCATGCGCTATCTGAGT     900
ValGlnValLeuIleGluLysTyrGluProAsnAsnSerLeuAlaArgLysGlyGlnIleSerValAspGlyPheMetArgTyrLeuSer     300

GGAGAAGAAAACGGAGTCGTTTCACCTGAGAAACTGGATTTGAATGAAGACATGTCTCAGCCCCTTTCTCACTATTTCATTAATTCCTCG     990
GlyGluGluAsnGlyValValSerProGluLysLeuAspLeuAsnGluAspMetSerGlnProLeuSerHisTyrPheIleAsnSerSer     330

CACAACACCTACCTCACAGCTGGCCAACTGGCTGGAAACTCCTCTGTTGAGATGTATCGCCAAGTGCTCCTGTCTGGTTGTCGCTGTGTG    1080
HisAsnThrTyrLeuThrAlaGlyGlnLeuAlaGlyAsnSerSerValGluMetTyrArgGlnValLeuLeuSerGlyCysArgCysVal     360
```

Additional file 1

```
GAGCTGGACTGCTGGAAGGGACGGACTGCAGAAGAGGAACCTGTCATCACCCATGGCTTCACCATGACAACTGAAATATCTTTCAAGGAA    1170
GluLeuAspCysTrpLysGlyArgThrAlaGluGluGluProValIleThrHisGlyPheThrMetThrThrGluIleSerPheLysGlu     390

GTGATAGAAGCAATTGCGGAGTGTGCATTTAAGACTTCACCTTTTCCAATTCTCCTTTCGTTTGAGAACCATGTGGATTCCCCAAAGCAG    1260
ValIleGluAlaIleAlaGluCysAlaPheLysThrSerProPheProIleLeuLeuSerPheGluAsnHisValAspSerProLysGln     420

CAAGCCAAGATGGCGGAGTACTGCCGACTGATCTTTGGGGATGCCCTTCTCATGGAGCCCCTGGAAAAATATCCACTGGAATCTGGAGTT    1350
GlnAlaLysMetAlaGluTyrCysArgLeuIlePheGlyAspAlaLeuLeuMetGluProLeuGluLysTyrProLeuGluSerGlyVal     450

CCTCTTCCAAGCCCTATGGATTTAATGTATAAAATTTTGGTGAAAAATAAGAAGAAATCACACAAGTCATCAGAAGGAAGCGGCAAAAAG    1440
ProLeuProSerProMetAspLeuMetTyrLysIleLeuValLysAsnLysLysLysSerHisLysSerSerGluGlySerGlyLysLys     480

AAGCTCTCAGAACAAGCCTCCAACACCTACAGTGACTCCTCCAGCATGTTCGAGCCCTCATCCCCAGGAGCCGGAGAAGCTGATACGGAA    1530
LysLeuSerGluGlnAlaSerAsnThrTyrSerAspSerSerSerMetPheGluProSerSerProGlyAlaGlyGluAlaAspThrGlu     510

AGTGACGACGACGATGATGATGATGACTGTAAAAAATCTTCAATGGATGAGGGGACTGCTGGAAGTGAGGCTATGGCCACAGAAGAAATG    1620
SerAspAspAspAspAspAspAspAspCysLysLysSerSerMetAspGluGlyThrAlaGlySerGluAlaMetAlaThrGluGluMet     540

TCTAATCTGGTGAACTATATTCAGCCAGTCAAGTTTGAGTCATTTGAAATTTCAAAAAAAAGAAATAAAAGTTTTGAAATGTCTTCCTTC    1710
SerAsnLeuValAsnTyrIleGlnProValLysPheGluSerPheGluIleSerLysLysArgAsnLysSerPheGluMetSerSerPhe     570

GTGGAAACCAAAGGACTTGAACAACTCACCAAGTCTCCAGTGGAATTTGTAGAATATAACAAAATGCAGCTTAGCAGGATATATCCAAAA    1800
ValGluThrLysGlyLeuGluGlnLeuThrLysSerProValGluPheValGluTyrAsnLysMetGlnLeuSerArgIleTyrProLys     600

GGAACACGTGTGGATTCATCCAACTATATGCCTCAGCTCTTCTGGAATGCAGGTTGTCAGATGGTGGCACTTAATTTCCAGACAATGGAC    1890
GlyThrArgValAspSerSerAsnTyrMetProGlnLeuPheTrpAsnAlaGlyCysGlnMetValAlaLeuAsnPheGlnThrMetAsp     630

CTGGCTATGCAAATAAATATGGGGATGTATGAATACAACGGGAAGAGTGGCTACAGATTGAAGCCAGAGTTCATGAGGAGGCCTGACAAG    1980
LeuAlaMetGlnIleAsnMetGlyMetTyrGluTyrAsnGlyLysSerGlyTyrArgLeuLysProGluPheMetArgArgProAspLys     660

CATTTTGATCCATTTACTGAAGGCATCGTAGATGGGATAGTGGCAAACACTTTGTCTGTTAAGATTATTTCAGGTCAGTTTCTTTCTGAT    2070
HisPheAspProPheThrGluGlyIleValAspGlyIleValAlaAsnThrLeuSerValLysIleIleSerGlyGlnPheLeuSerAsp     690

AAGAAAGTTGGGACTTACGTGGAAGTAGATATGTTTGGTTTGCCTGTGGATACAAGGAGGAAGGCATTTAAGACCAAAACATCCCAAGGA    2160
LysLysValGlyThrTyrValGluValAspMetPheGlyLeuProValAspThrArgArgLysAlaPheLysThrLysThrSerGlnGly     720
```

Additional file 1

```
AATGCTGTGAATCCTGTCTGGGAAGAAGAACCTATTGTGTTCAAAAAGGTGGTTCTTCCTACTCTGGCCTGTTTGAGAATAGCAGTTTAT   2250
AsnAlaValAsnProValTrpGluGluGluProIleValPheLysLysValValLeuProThrLeuAlaCysLeuArgIleAlaValTyr    750

GAAGAAGGAGGTAAATTCATTGGCCACCGTATCTTGCCAGTGCAAGCCATTCGGCCAGGCTATCACTATATCTGTCTAAGGAATGAAAGG   2340
GluGluGlyGlyLysPheIleGlyHisArgIleLeuProValGlnAlaIleArgProGlyTyrHisTyrIleCysLeuArgAsnGluArg    780

AACCAGCCTCTGACGCTGCCTGCTGTCTTTGTCTACATAGAAGTGAAAGACTATGTGCCAGACACATATGCAGATGTCATCGAAGCTTTA   2430
AsnGlnProLeuThrLeuProAlaValPheValTyrIleGluValLysAspTyrValProAspThrTyrAlaAspValIleGluAlaLeu    810

TCAAACCCAATCCGATATGTGAACCTGATGGAACAGAGAGCTAAGCAATTGGCTGCTTTGACACTGGAAGATGAAGAAGAAGTAAAGAAA   2520
SerAsnProIleArgTyrValAsnLeuMetGluGlnArgAlaLysGlnLeuAlaAlaLeuThrLeuGluAspGluGluGluValLysLys    840

GAGGCTGATCCTGGAGAAACACCATCAGAGGCTCCAAGTGAAGCGAGAACGACTCCAGCAGAAAATGGGGTGAATCACACTACAACCCTG   2610
GluAlaAspProGlyGluThrProSerGluAlaProSerGluAlaArgThrThrProAlaGluAsnGlyValAsnHisThrThrThrLeu    870

ACACCCAAGCCACCCTCCCAGGCTCTCCACAGCCAGCCAGCTCCAGGTTCTGTAAAGGCACCTGCCAAAACAGAAGATCTTATTCAGAGT   2700
ThrProLysProProSerGlnAlaLeuHisSerGlnProAlaProGlySerValLysAlaProAlaLysThrGluAspLeuIleGlnSer    900

GTCTTAACAGAAGTGGAAGCACAGACCATCGAAGAACTAAAGCAACAGAAATCGTTTGTGAAACTTCAAAAGAAACACTACAAAGAAATG   2790
ValLeuThrGluValGluAlaGlnThrIleGluGluLeuLysGlnGlnLysSerPheValLysLeuGlnLysLysHisTyrLysGluMet    930

AAAGACCTGGTTAAGAGACACCACAAGAAAACCACTGACCTTATCAAAGAACACACTACCAAGTATAATGAAATTCAGAATGACTACTTG   2880
LysAspLeuValLysArgHisHisLysLysThrThrAspLeuIleLysGluHisThrThrLysTyrAsnGluIleGlnAsnAspTyrLeu    960

AGAAGGAGAGCCGCTTTGGAAAAGTCCGCCAAAAAGGACAGTAAGAAAAAATCGGAACCCAGCAGCCCTGATCATGGTTCATCAACGATT   2970
ArgArgArgAlaAlaLeuGluLysSerAlaLysLysAspSerLysLysLysSerGluProSerSerProAspHisGlySerSerThrIle    990

GAGCAAGACCTCGCTGCCCTGGATGCTGAAATGACCCAAAAGTTAATAGACTTGAAGGACAAACAACAGCAGCAGCTGCTTAATCTTCGG   3060
GluGlnAspLeuAlaAlaLeuAspAlaGluMetThrGlnLysLeuIleAspLeuLysAspLysGlnGlnGlnGlnLeuLeuAsnLeuArg   1020

CAAGAACAGTATTATAGTGAAAAATACCAGAAGCGAGAACATATTAAACTGCTTATTCAAAAGTTGACGGATGTCGCAGAAGAGTGTCAG   3150
GlnGluGlnTyrTyrSerGluLysTyrGlnLysArgGluHisIleLysLeuLeuIleGlnLysLeuThrAspValAlaGluGluCysGln   1050

AACAATCAGTTAAAGAAGCTCAAAGAAATCTGTGAGAAAGAAAAGAAAGAATTAAAGAAGAAAATGGATAAAAAGAGGCAGGAGAAGATA   3240
AsnAsnGlnLeuLysLysLeuLysGluIleCysGluLysGluLysLysGluLeuLysLysLysMetAspLysLysArgGlnGluLysIle   1080
```

Additional file 1

```
ACAGAAGCTAAATCCAAAGACAAAAGTCAGATGGAAGAGGAGAAGACAGAGATGATCCGGTCATATATCCAGGAAGTGGTGCAGTATATC   3330
ThrGluAlaLysSerLysAspLysSerGlnMetGluGluGluLysThrGluMetIleArgSerTyrIleGlnGluValValGlnTyrIle   1110

AAGAGGCTAGAAGAAGCGCAAAGTAAACGGCAAGAAAAACTCGTAGAGAAACACAAGGAAATACGTCAGCAGATCCTGGATGAAAAGCCC   3420
LysArgLeuGluGluAlaGlnSerLysArgGlnGluLysLeuValGluLysHisLysGluIleArgGlnGlnIleLeuAspGluLysPro   1140

AAGCTGCAGGTGGAGCTGGAGCAAGAATACCAAGACAAATTCAAAAGACTGCCCCTCGAGATTTTGGAATTCGTGCAGGAAGCCATGAAA   3510
LysLeuGlnValGluLeuGluGlnGluTyrGlnAspLysPheLysArgLeuProLeuGluIleLeuGluPheValGlnGluAlaMetLys   1170

GGAAAGATCAGTGAAGACAGCAATCACGGTTCTGCCCCTCTCTCCCTGTCCTCAGACCCTGGAAAAGTGAACCACAAGACTCCCTCCAGT   3600
GlyLysIleSerGluAspSerAsnHisGlySerAlaProLeuSerLeuSerSerAspProGlyLysValAsnHisLysThrProSerSer   1200

GAGGAGCTGGGAGGAGACATCCCAGGAAAAGAATTTGATACTCCTCTGTGAatgctcctgccaggccttcagaaattgcatggcggccgc   3690
GluGluLeuGlyGlyAspIleProGlyLysGluPheAspThrProLeu *                                           1216
```

Additional file 2

```
tggcttatcgaaattaatacgactcactatagggagacccaagcttggtaccgagcttcgatccaccagatatccagtaccggtgccacc     -1

ATGGCCGGGGCTCAACCCGGTGTGCACGCCTTGCAACTCAAGCCCGTGTGCGTGTCCGACAGCCTCAAGAAGGGCACCAAATTCGTCAAG     90
MetAlaGlyAlaGlnProGlyValHisAlaLeuGlnLeuLysProValCysValSerAspSerLeuLysLysGlyThrLysPheValLys     30

TGGGATGATGATTCAACTATTGTTACTCCAATTATTTTGAGGACTGACCCTCAGGGATTTTTCTTTTACTGGACAGATCAAAACAAGGAG    180
TrpAspAspAspSerThrIleValThrProIleIleLeuArgThrAspProGlnGlyPhePhePheTyrTrpThrAspGlnAsnLysGlu     60

ACAGAGCTACTGGATCTCAGCCTTGTCAAAGATGCCAGATGTGGGAGACACGCCAAAGCTCCCAAGGACCCCAAATTACGTGAACTTTTG    270
ThrGluLeuLeuAspLeuSerLeuValLysAspAlaArgCysGlyArgHisAlaLysAlaProLysAspProLysLeuArgGluLeuLeu     90

GATGTGGGGAACATCGGGCGCCTGGAGCAGCGCATGATCACAGTGGTGTATGGGCCTGACCTCGTGAACATCTCCCATTTGAATCTCGTG    360
AspValGlyAsnIleGlyArgLeuGluGlnArgMetIleThrValValTyrGlyProAspLeuValAsnIleSerHisLeuAsnLeuVal    120

GCTTTCCAAGAAGAAGTGGCCAAGGAATGGACAAATGAGGTTTTCAGTTTGGCAACAAACCTGCTGGCCCAAAACATGTCCAGGGATGCA    450
AlaPheGlnGluGluValAlaLysGluTrpThrAsnGluValPheSerLeuAlaThrAsnLeuLeuAlaGlnAsnMetSerArgAspAla    150

TTTCTGGAAAAAGCCTATACTAAACTTAAGCTGCAAGTCACTCCAGAAGGGCGTATTCCTCTCAAAAACATATATCGCTTGTTTTCAGCA    540
PheLeuGluLysAlaTyrThrLysLeuLysLeuGlnValThrProGluGlyArgIleProLeuLysAsnIleTyrArgLeuPheSerAla    180

GATCGGAAGCGAGTTGAAACTGCTTTAGAGGCTTGTAGTCTTCCATCTTCAAGGAATGATTCAATACCTCAAGAAGATTTCACTCCAGAA    630
AspArgLysArgValGluThrAlaLeuGluAlaCysSerLeuProSerSerArgAsnAspSerIleProGlnGluAspPheThrProGlu    210

GTGTACAGAGTTTTCCTCAACAACCTTTGCCCTCGACCTGAAATTGATAACATCTTTTCAGAATTTGGTGCAAAAAGCAAACCATATCTT    720
ValTyrArgValPheLeuAsnAsnLeuCysProArgProGluIleAspAsnIlePheSerGluPheGlyAlaLysSerLysProTyrLeu    240

ACCGTTGATCAGATGATGGATTTTATCAACCTTAAGCAGCGAGATCCTCGGCTTAATGAAATACTTTATCCACCTCTAAAACAAGAGCAA    810
ThrValAspGlnMetMetAspPheIleAsnLeuLysGlnArgAspProArgLeuAsnGluIleLeuTyrProProLeuLysGlnGluGln    270

GTCCAAGTATTGATTGAGAAGTATGAACCCAACAACAGCCTCGCCAGAAAAGGACAAATATCAGTGGATGGGTTCATGCGCTATCTGAGT    900
ValGlnValLeuIleGluLysTyrGluProAsnAsnSerLeuAlaArgLysGlyGlnIleSerValAspGlyPheMetArgTyrLeuSer    300

GGAGAAGAAAACGGAGTCGTTTCACCTGAGAAACTGGATTTGAATGAAGACATGTCTCAGCCCCTTTCTCACTATTTCATTAATTCCTCG    990
GlyGluGluAsnGlyValValSerProGluLysLeuAspLeuAsnGluAspMetSerGlnProLeuSerHisTyrPheIleAsnSerSer    330

CACAACACCTACCTCACAGCTGGCCAACTGGCTGGAAACTCCTCTGTTGAGATGTATCGCCAAGTGCTCCTGTCTGGTTGTCGCTGTGTG   1080
HisAsnThrTyrLeuThrAlaGlyGlnLeuAlaGlyAsnSerSerValGluMetTyrArgGlnValLeuLeuSerGlyCysArgCysVal    360
```

Additional file 2

```
GAGCTGGACTGCTGGAAGGGACGGACTGCAGAAGAGGAACCTGTCATCACCCATGGCTTCACCATGACAACTGAAATATCTTTCAAGGAA    1170
GluLeuAspCysTrpLysGlyArgThrAlaGluGluGluProValIleThrHisGlyPheThrMetThrThrGluIleSerPheLysGlu     390

GTGATAGAAGCAATTGCGGAGTGTGCATTTAAGACTTCACCTTTTCCAATTCTCCTTTCGTTTGAGAACCATGTGGATTCCCCAAAGCAG    1260
ValIleGluAlaIleAlaGluCysAlaPheLysThrSerProPheProIleLeuLeuSerPheGluAsnHisValAspSerProLysGln     420

CAAGCCAAGATGGCGGAGTACTGCCGACTGATCTTTGGGGATGCCCTTCTCATGGAGCCCCTGGAAAAATATCCACTGGAATCTGGAGTT    1350
GlnAlaLysMetAlaGluTyrCysArgLeuIlePheGlyAspAlaLeuLeuMetGluProLeuGluLysTyrProLeuGluSerGlyVal     450

CCTCTTCCAAGCCCTATGGATTTAATGTATAAAATTTTGGTGAAAAATAAGAAGAAATCACACAAGTCATCAGAAGGAAGCGGCAAAAAG    1440
ProLeuProSerProMetAspLeuMetTyrLysIleLeuValLysAsnLysLysLysSerHisLysSerSerGluGlySerGlyLysLys     480

AAGCTCTCAGAACAAGCCTCCAACACCTACAGTGACTCCTCCAGCATGTTCGAGCCCTCATCCCCAGGAGCCGGAGAAGCTGATACGGAA    1530
LysLeuSerGluGlnAlaSerAsnThrTyrSerAspSerSerSerMetPheGluProSerSerProGlyAlaGlyGluAlaAspThrGlu     510

AGTGACGACGACGATGATGATGATGACTGTAAAAAATCTTCAATGGATGAGGGGACTGCTGGAAGTGAGGCTATGGCCACAGAAGAAATG    1620
SerAspAspAspAspAspAspAspCysLysLysSerSerMetAspGluGlyThrAlaGlySerGluAlaMetAlaThrGluGluMet     540

TCTAATCTGGTGAACTATATTCAGCCAGTCAAGTTTGAGTCATTTGAAATTTCAAAAAAAAGAAATAAAAGTTTTGAAATGTCTTCCTTC    1710
SerAsnLeuValAsnTyrIleGlnProValLysPheGluSerPheGluIleSerLysLysArgAsnLysSerPheGluMetSerSerPhe     570

GTGGAAACCAAAGGACTTGAACAACTCACCAAGTCTCCAGTGGAATTTGTAGAATATAACAAAATGCAGCTTAGCAGGATATATCCAAAA    1800
ValGluThrLysGlyLeuGluGlnLeuThrLysSerProValGluPheValGluTyrAsnLysMetGlnLeuSerArgIleTyrProLys     600

GGAACACGTGTGGATTCATCCAACTATATGCCTCAGCTCTTCTGGAATGCAGGTTGTCAGATGGTGGCACTTAATTTCCAGACAATGGAC    1890
GlyThrArgValAspSerSerAsnTyrMetProGlnLeuPheTrpAsnAlaGlyCysGlnMetValAlaLeuAsnPheGlnThrMetAsp     630

CTGGCTATGCAAATAAATATGGGGATGTATGAATACAACGGGAAGAGTGGCTACAGATTGAAGCCAGAGTTCATGAGGAGGCCTGACAAG    1980
LeuAlaMetGlnIleAsnMetGlyMetTyrGluTyrAsnGlyLysSerGlyTyrArgLeuLysProGluPheMetArgArgProAspLys     660

CATTTTGATCCATTTACTGAAGGCATCGTAGATGGGATAGTGGCAAACACTTTGTCTGTTAAGATTATTTCAGGTCAGTTTCTTTCTGAT    2070
HisPheAspProPheThrGluGlyIleValAspGlyIleValAlaAsnThrLeuSerValLysIleIleSerGlyGlnPheLeuSerAsp     690

AAGAAAGTTGGGACTTACGTGGAAGTAGATATGTTTGGTTTGCCTGTGGATACAAGGAGGAAGGCATTTAAGACCAAAACATCCCAAGGA    2160
LysLysValGlyThrTyrValGluValAspMetPheGlyLeuProValAspThrArgArgLysAlaPheLysThrLysThrSerGlnGly     720
```

Additional file 2

```
AATGCTGTGAATCCTGTCTGGGAAGAAGAACCTATTGTGTTCAAAAAGGTGGTTCTTCCTACTCTGGCCTGTTTGAGAATAGCAGTTTAT    2250
AsnAlaValAsnProValTrpGluGluGluProIleValPheLysLysValValLeuProThrLeuAlaCysLeuArgIleAlaValTyr     750

GAAGAAGGAGGTAAATTCATTGGCCACCGTATCTTGCCAGTGCAAGCCATTCGGCCAGGCTATCACTATATCTGTCTAAGGAATGAAAGG    2340
GluGluGlyGlyLysPheIleGlyHisArgIleLeuProValGlnAlaIleArgProGlyTyrHisTyrIleCysLeuArgAsnGluArg     780

AACCAGCCTCTGACGCTGCCTGCTGTCTTTGTCTACATAGAAGTGAAAGACTATGTGCCAGACACATATGCAGATGTCATCGAAGCTTTA    2430
AsnGlnProLeuThrLeuProAlaValPheValTyrIleGluValLysAspTyrValProAspThrTyrAlaAspValIleGluAlaLeu     810

TCAAACCCAATCCGATATGTGAACCTGATGGAACAGAGAGCTAAGCAATTGGCTGCTTTGACACTGGAAGATGAAGAAGAAGTAAAGAAA    2520
SerAsnProIleArgTyrValAsnLeuMetGluGlnArgAlaLysGlnLeuAlaAlaLeuThrLeuGluAspGluGluGluValLysLys     840

GAGGCTGATCCTGGAGAAACACCATCAGAGGCTCCAAGTGAAGCGAGAACGACTCCAGCAGAAAATGGGGTGAATCACACTACAACCCTG    2610
GluAlaAspProGlyGluThrProSerGluAlaProSerGluAlaArgThrThrProAlaGluAsnGlyValAsnHisThrThrThrLeu     870

ACACCCAAGCCACCCTCCCAGGCTCTCCACAGCCAGCCAGCTCCAGGTTCTGTAAAGGCACCTGCCAAAACAGAAGATCTTATTCAGAGT    2700
ThrProLysProProSerGlnAlaLeuHisSerGlnProAlaProGlySerValLysAlaProAlaLysThrGluAspLeuIleGlnSer     900

GTCTTAACAGAAGTGGAAGCACAGACCATCGAAGAACTAAAGCAACAGAAATCGTTTGTGAAACTTCAAAAGAAACACTACAAAGAAATG    2790
ValLeuThrGluValGluAlaGlnThrIleGluGluLeuLysGlnGlnLysSerPheValLysLeuGlnLysLysHisTyrLysGluMet     930

AAAGACCTGGTTAAGAGACACCACAAGAAAACCACTGACCTTATCAAAGAACACACTACCAAGTATAATGAAATTCAGAATGACTACTTG    2880
LysAspLeuValLysArgHisHisLysLysThrThrAspLeuIleLysGluHisThrThrLysTyrAsnGluIleGlnAsnAspTyrLeu     960

AGAAGGAGAGCCGCTTTGGAAAAGTCCGCCAAAAAGGACAGTAAGAAAAAATCGGAACCCAGCAGCCCTGATCATGGTTCATCAACGATT    2970
ArgArgArgAlaAlaLeuGluLysSerAlaLysLysAspSerLysLysLysSerGluProSerSerProAspHisGlySerSerThrIle     990

GAGCAAGACCTCGCTGCCCTGGATGCTGAAATGACCCAAAAGTTAATAGACTTGAAGGACAAACAACAGCAGCAGCTGCTTAATCTTCGG    3060
GluGlnAspLeuAlaAlaLeuAspAlaGluMetThrGlnLysLeuIleAspLeuLysAspLysGlnGlnGlnGlnLeuLeuAsnLeuArg    1020

CAAGAACAGTATTATAGTGAAAAATACCAGAAGCGAGAACATATTAAACTGCTTATTCAAAAGTTGACGGATGTCGCAGAAGAGTGTCAG    3150
GlnGluGlnTyrTyrSerGluLysTyrGlnLysArgGluHisIleLysLeuLeuIleGlnLysLeuThrAspValAlaGluGluCysGln    1050

AACAATCAGTTAAAGAAGCTCAAAGAAATCTGTGAGAAAGAAAAGAAAGAATTAAAGAAGAAAATGGATAAAAAGAGGCAGGAGAAGATA    3240
AsnAsnGlnLeuLysLysLeuLysGluIleCysGluLysGluLysLysGluLeuLysLysLysMetAspLysLysArgGlnGluLysIle    1080
```

Additional file 2

```
ACAGAAGCTAAATCCAAAGACAAAAGTCAGATGGAAGAGGAGAAGACAGAGATGATCCGGTCATATATCCAGGAAGTGGTGCAGTATATC      3330
ThrGluAlaLysSerLysAspLysSerGlnMetGluGluGluLysThrGluMetIleArgSerTyrIleGlnGluValValGlnTyrIle      1110

AAGAGGCTAGAAGAAGCGCAAAGTAAACGGCAAGAAAAACTCGTAGAGAAACACAAGGAAATACGTCAGCAGATCCTGGATGAAAAGCCC      3420
LysArgLeuGluGluAlaGlnSerLysArgGlnGluLysLeuValGluLysHisLysGluIleArgGlnGlnIleLeuAspGluLysPro      1140

AAGGGGGAAGGTTCCTCCTCATTCTTGTCGGAAACTTGCCATGAGGATCCCTCTGTTTCCCCCAACTTTACTCCCCCCAACCCTCAAGCT      3510
LysGlyGluGlySerSerSerPheLeuSerGluThrCysHisGluAspProSerValSerProAsnPheThrProProAsnProGlnAla      1170

CTCAAGTGGTGAtctagattgcggccgctcgagcatgcatctagagggccctattctatagtgtcacctaaatgctagagctcgctgatc      1270
LeuLysTrp *                                                                                     1173
```

Additional file 3

tggcttatcgaaattaatacgactcactatagggagacccaagcttggtaccgagcttcgatccaccagatatccagtaccggtgccacc -1

ATGGCCGGGGCTCAACCCGGTGTGCACGCCTTGCAACTCAAGCCCGTGTGCGTGTCCGACAGCCTCAAGAAGGGCACCAAATTCGTCAAG 90
MetAlaGlyAlaGlnProGlyValHisAlaLeuGlnLeuLysProValCysValSerAspSerLeuLysLysGlyThrLysPheValLys 30

TGGGATGATGATTCAACTATTGTTACTCCAATTATTTTGAGGACTGACCCTCAGGGATTTTTCTTTTACTGGACAGATCAAAACAAGGAG 180
TrpAspAspAspSerThrIleValThrProIleIleLeuArgThrAspProGlnGlyPhePhePheTyrTrpThrAspGlnAsnLysGlu 60

ACAGAGCTACTGGATCTCAGCCTTGTCAAAGATGCCAGATGTGGGAGACACGCCAAAGCTCCCAAGGACCCCAAATTACGTGAACTTTTG 270
ThrGluLeuLeuAspLeuSerLeuValLysAspAlaArgCysGlyArgHisAlaLysAlaProLysAspProLysLeuArgGluLeuLeu 90

GATGTGGGGAACATCGGGCGCCTGGAGCAGCGCATGATCACAGTGGTGTATGGGCCTGACCTCGTGAACATCTCCCATTTGAATCTCGTG 360
AspValGlyAsnIleGlyArgLeuGluGlnArgMetIleThrValValTyrGlyProAspLeuValAsnIleSerHisLeuAsnLeuVal 120

GCTTTCCAAGAAGAAGTGGCCAAGGAATGGACAAATGAGGTTTTCAGTTTGGCAACAAACCTGCTGGCCCAAAACATGTCCAGGGATGCA 450
AlaPheGlnGluGluValAlaLysGluTrpThrAsnGluValPheSerLeuAlaThrAsnLeuLeuAlaGlnAsnMetSerArgAspAla 150

TTTCTGGAAAAAGCCTATACTAAACTTAAGCTGCAAGTCACTCCAGAAGGGCGTATTCCTCTCAAAAACATATATCGCTTGTTTTCAGCA 540
PheLeuGluLysAlaTyrThrLysLeuLysLeuGlnValThrProGluGlyArgIleProLeuLysAsnIleTyrArgLeuPheSerAla 180

GATCGGAAGCGAGTTGAAACTGCTTTAGAGGCTTGTAGTCTTCCATCTTCAAGGAATGATTCAATACCTCAAGAAGATTTCACTCCAGAA 630
AspArgLysArgValGluThrAlaLeuGluAlaCysSerLeuProSerSerArgAsnAspSerIleProGlnGluAspPheThrProGlu 210

GTGTACAGAGTTTTCCTCAACAACCTTTGCCCTCGACCTGAAATTGATAACATCTTTTCAGAATTTGGTGCAAAAAGCAAACCATATCTT 720
ValTyrArgValPheLeuAsnAsnLeuCysProArgProGluIleAspAsnIlePheSerGluPheGlyAlaLysSerLysProTyrLeu 240

ACCGTTGATCAGATGATGGATTTTATCAACCTTAAGCAGCGAGATCCTCGGCTTAATGAAATACTTTATCCACCTCTAAAACAAGAGCAA 810
ThrValAspGlnMetMetAspPheIleAsnLeuLysGlnArgAspProArgLeuAsnGluIleLeuTyrProProLeuLysGlnGluGln 270

GTCCAAGTATTGATTGAGAAGTATGAACCCAACAACAGCCTCGCCAGAAAAGGACAAATATCAGTGGATGGGTTCATGCGCTATCTGAGT 900
ValGlnValLeuIleGluLysTyrGluProAsnAsnSerLeuAlaArgLysGlyGlnIleSerValAspGlyPheMetArgTyrLeuSer 300

GGAGAAGAAAACGGAGTCGTTTCACCTGAGAAACTGGATTTGAATGAAGACATGTCTCAGCCCCTTTCTCACTATTTCATTAATTCCTCG 990
GlyGluGluAsnGlyValValSerProGluLysLeuAspLeuAsnGluAspMetSerGlnProLeuSerHisTyrPheIleAsnSerSer 330

CACAACACCTACCTCACAGCTGGCCAACTGGCTGGAAACTCCTCTGTTGAGATGTATCGCCAAGTGCTCCTGTCTGGTTGTCGCTGTGTG 1080
HisAsnThrTyrLeuThrAlaGlyGlnLeuAlaGlyAsnSerSerValGluMetTyrArgGlnValLeuLeuSerGlyCysArgCysVal 360

Additional file 3

```
GAGCTGGACTGCTGGAAGGGACGGACTGCAGAAGAGGAACCTGTCATCACCCATGGCTTCACCATGACAACTGAAATATCTTTCAAGGAA   1170
GluLeuAspCysTrpLysGlyArgThrAlaGluGluGluProValIleThrHisGlyPheThrMetThrThrGluIleSerPheLysGlu    390

GTGATAGAAGCAATTGCGGAGTGTGCATTTAAGACTTCACCTTTTCCAATTCTCCTTTCGTTTGAGAACCATGTGGATTCCCCAAAGCAG   1260
ValIleGluAlaIleAlaGluCysAlaPheLysThrSerProPheProIleLeuLeuSerPheGluAsnHisValAspSerProLysGln    420

CAAGCCAAGATGGCGGAGTACTGCCGACTGATCTTTGGGGATGCCCTTCTCATGGAGCCCCTGGAAAAATATCCACTGGAATCTGGAGTT   1350
GlnAlaLysMetAlaGluTyrCysArgLeuIlePheGlyAspAlaLeuLeuMetGluProLeuGluLysTyrProLeuGluSerGlyVal    450

CCTCTTCCAAGCCCTATGGATTTAATGTATAAAATTTTGGTGAAAAATAAGAAGAAATCACACAAGTCATCAGAAGGAAGCGGCAAAAAG   1440
ProLeuProSerProMetAspLeuMetTyrLysIleLeuValLysAsnLysLysLysSerHisLysSerSerGluGlySerGlyLysLys    480

AAGCTCTCAGAACAAGCCTCCAACACCTACAGTGACTCCTCCAGCATGTTCGAGCCCTCATCCCCAGGAGCCGGAGAAGCTGATACGGAA   1530
LysLeuSerGluGlnAlaSerAsnThrTyrSerAspSerSerSerMetPheGluProSerSerProGlyAlaGlyGluAlaAspThrGlu    510

AGTGACGACGACGATGATGATGATGACTGTAAAAAATCTTCAATGGATGAGGGGACTGCTGGAAGTGAGGCTATGGCCACAGAAGAAATG   1620
SerAspAspAspAspAspAspAspAspCysLysLysSerSerMetAspGluGlyThrAlaGlySerGluAlaMetAlaThrGluGluMet    540

TCTAATCTGGTGAACTATATTCAGCCAGTCAAGTTTGAGTCATTTGAAATTTCAAAAAAAAGAAATAAAAGTTTTGAAATGTCTTCCTTC   1710
SerAsnLeuValAsnTyrIleGlnProValLysPheGluSerPheGluIleSerLysLysArgAsnLysSerPheGluMetSerSerPhe    570

GTGGAAACCAAAGGACTTGAACAACTCACCAAGTCTCCAGTGGAATTTGTAGAATATAACAAAATGCAGCTTAGCAGGATATATCCAAAA   1800
ValGluThrLysGlyLeuGluGlnLeuThrLysSerProValGluPheValGluTyrAsnLysMetGlnLeuSerArgIleTyrProLys    600

GGAACACGTGTGGATTCATCCAACTATATGCCTCAGCTCTTCTGGAATGCAGGTTGTCAGATGGTGGCACTTAATTTCCAGACAATGGAC   1890
GlyThrArgValAspSerSerAsnTyrMetProGlnLeuPheTrpAsnAlaGlyCysGlnMetValAlaLeuAsnPheGlnThrMetAsp    630

CTGGCTATGCAAATAAATATGGGGATGTATGAATACAACGGGAAGAGTGGCTACAGATTGAAGCCAGAGTTCATGAGGAGGCCTGACAAG   1980
LeuAlaMetGlnIleAsnMetGlyMetTyrGluTyrAsnGlyLysSerGlyTyrArgLeuLysProGluPheMetArgArgProAspLys    660

CATTTTGATCCATTTACTGAAGGCATCGTAGATGGGATAGTGGCAAACACTTTGTCTGTTAAGATTATTTCAGGTCAGTTTCTTTCTGAT   2070
HisPheAspProPheThrGluGlyIleValAspGlyIleValAlaAsnThrLeuSerValLysIleIleSerGlyGlnPheLeuSerAsp    690

AAGAAAGTTGGGACTTACGTGGAAGTAGATATGTTTGGTTTGCCTGTGGATACAAGGAGGAAGGCATTTAAGACCAAAACATCCCAAGGA   2160
LysLysValGlyThrTyrValGluValAspMetPheGlyLeuProValAspThrArgArgLysAlaPheLysThrLysThrSerGlnGly    720
```

Additional file 3

AATGCTGTGAATCCTGTCTGGGAAGAAGAACCTATTGTGTTCAAAAAGGTGGTTCTTCCTACTCTGGCCTGTTTGAGAATAGCAGTTTAT 2250
AsnAlaValAsnProValTrpGluGluGluProIleValPheLysLysValValLeuProThrLeuAlaCysLeuArgIleAlaValTyr 750

GAAGAAGGAGGTAAATTCATTGGCCACCGTATCTTGCCAGTGCAAGCCATTCGGCCAGGCTATCACTATATCTGTCTAAGGAATGAAAGG 2340
GluGluGlyGlyLysPheIleGlyHisArgIleLeuProValGlnAlaIleArgProGlyTyrHisTyrIleCysLeuArgAsnGluArg 780

AACCAGCCTCTGACGCTGCCTGCTGTCTTTGTCTACATAGAAGTGAAAGACTATGTGCCAGACACATATGCAGATGTCATCGAAGCTTTA 2430
AsnGlnProLeuThrLeuProAlaValPheValTyrIleGluValLysAspTyrValProAspThrTyrAlaAspValIleGluAlaLeu 810

TCAAACCCAATCCGATATGTGAACCTGATGGAACAGAGAGCTAAGCAATTGGCTGCTTTGACACTGGAAGATGAAGAAGAAGTAAAGAAA 2520
SerAsnProIleArgTyrValAsnLeuMetGluGlnArgAlaLysGlnLeuAlaAlaLeuThrLeuGluAspGluGluGluValLysLys 840

GAGGCTGATCCTGGAGAAACACCATCAGAGGCTCCAAGTGAAGCGAGAACGACTCCAGCAGAAAATGGGGTGAATCACACTACAACCCTG 2610
GluAlaAspProGlyGluThrProSerGluAlaProSerGluAlaArgThrThrProAlaGluAsnGlyValAsnHisThrThrThrLeu 870

ACACCCAAGCCACCCTCCCAGGCTCTCCACAGCCAGCCAGCTCCAGGTTCTGTAAAGGCACCTGCCAAAACAGAAGATCTTATTCAGAGT 2700
ThrProLysProProSerGlnAlaLeuHisSerGlnProAlaProGlySerValLysAlaProAlaLysThrGluAspLeuIleGlnSer 900

GTCTTAACAGAAGTGGAAGCACAGACCATCGAAGAACTAAAGCAACAGAAATCGTTTGTGAAACTTCAAAAGAAACACTACAAAGAAATG 2790
ValLeuThrGluValGluAlaGlnThrIleGluGluLeuLysGlnGlnLysSerPheValLysLeuGlnLysLysHisTyrLysGluMet 930

AAAGACCTGGTTAAGAGACACCACAAGAAAACCACTGACCTTATCAAAGAACACACTACCAAGTATAATGAAATTCAGAATGACTACTTG 2880
LysAspLeuValLysArgHisHisLysLysThrThrAspLeuIleLysGluHisThrThrLysTyrAsnGluIleGlnAsnAspTyrLeu 960

AGAAGGAGAGCCGCTTTGGAAAAGTCCGCCAAAAAGGACAGTAAGAAAAAATCGGAACCCAGCAGCCCTGATCATGGTTCATCAACGATT 2970
ArgArgArgAlaAlaLeuGluLysSerAlaLysLysAspSerLysLysLysSerGluProSerSerProAspHisGlySerSerThrIle 990

GAGCAAGACCTCGCTGCCCTGGATGCTGAAATGACCCAAAAGTTAATAGACTTGAAGGACAAACAACAGCAGCAGCTGCTTAATCTTCGG 3060
GluGlnAspLeuAlaAlaLeuAspAlaGluMetThrGlnLysLeuIleAspLeuLysAspLysGlnGlnGlnGlnLeuLeuAsnLeuArg 1020

CAAGAACAGTATTATAGTGAAAAATACCAGAAGCGAGAACATATTAAACTGCTTATTCAAAAGTTGACGGATGTCGCAGAAGAGTGTCAG 3150
GlnGluGlnTyrTyrSerGluLysTyrGlnLysArgGluHisIleLysLeuLeuIleGlnLysLeuThrAspValAlaGluGluCysGln 1050

AACAATCAGTTAAAG**ATA**CTCAAAGAAATCTGTGAG**ATA**GAAAAGAAAGAATTAAAGAAG**ATA**ATGGATAAAAAGAGGCAGGAGAAGATA 3240
AsnAsnGlnLeuLys**Ile**LeuLysGluIleCysGlu**Ile**GluLysLysGluLeuLysLys**Ile**MetAspLysLysArgGlnGluLysIle 1080

Additional file 3

```
ACAGAAGCTAAATCCAAAGACAAAAGTCAGATGGAAGAGGAGAAGACAGAGATGATCCGGTCATATATCCAGGAAGTGGTGCAGTATATC     3330
ThrGluAlaLysSerLysAspLysSerGlnMetGluGluGluLysThrGluMetIleArgSerTyrIleGlnGluValValGlnTyrIle     1110

AAGAGGCTAGAAGAAGCGCAAAGTAAACGGCAAGAAAAACTCGTAGAGAAACACAAGGAAATACGTCAGCAGATCCTGGATGAAAAGCCC     3420
LysArgLeuGluGluAlaGlnSerLysArgGlnGluLysLeuValGluLysHisLysGluIleArgGlnGlnIleLeuAspGluLysPro     1140

AAGCTGCAGGTGGAGCTGGAGCAAGAATACCAAGACAAATTCAAAAGACTGCCCCTCGAGATTTTGGAATTCGTGCAGGAAGCCATGAAA     3510
LysLeuGlnValGluLeuGluGlnGluTyrGlnAspLysPheLysArgLeuProLeuGluIleLeuGluPheValGlnGluAlaMetLys     1170

GGAAAGATCAGTGAAGACAGCAATCACGGTTCTGCCCCTCTCTCCCTGTCCTCAGACCCTGGAAAAGTGAACCACAAGACTCCCTCCAGT     3600
GlyLysIleSerGluAspSerAsnHisGlySerAlaProLeuSerLeuSerSerAspProGlyLysValAsnHisLysThrProSerSer     1200

GAGGAGCTGGGAGGAGACATCCCAGGAAAAGAATTTGATACTCCTCTGTGAatgctcctgccaggccttcagaaattgcatggcggccgc     3690
GluGluLeuGlyGlyAspIleProGlyLysGluPheAspThrProLeu *                                             1216
```

Additional file 4

```
tggcttatcgaaattaatacgactcactatagggagacccaagcttggtaccgagcttcgatccaccagatatccagtaccggtgccacc     -1

ATGGCCGGGGCTCAACCCGGTGTGCACGCCTTGCAACTCAAGCCCGTGTGCGTGTCCGACAGCCTCAAGAAGGGCACCAAATTCGTCAAG     90
MetAlaGlyAlaGlnProGlyValHisAlaLeuGlnLeuLysProValCysValSerAspSerLeuLysLysGlyThrLysPheValLys     30

TGGGATGATGATTCAACTATTGTTACTCCAATTATTTTGAGGACTGACCCTCAGGGATTTTTCTTTTACTGGACAGATCAAAACAAGGAG    180
TrpAspAspAspSerThrIleValThrProIleIleLeuArgThrAspProGlnGlyPhePhePheTyrTrpThrAspGlnAsnLysGlu     60

ACAGAGCTACTGGATCTCAGCCTTGTCAAAGATGCCAGATGTGGGAGACACGCCAAAGCTCCCAAGGACCCCAAATTACGTGAACTTTTG    270
ThrGluLeuLeuAspLeuSerLeuValLysAspAlaArgCysGlyArgHisAlaLysAlaProLysAspProLysLeuArgGluLeuLeu     90

GATGTGGGGAACATCGGGCGCCTGGAGCAGCGCATGATCACAGTGGTGTATGGGCCTGACCTCGTGAACATCTCCCATTTGAATCTCGTG    360
AspValGlyAsnIleGlyArgLeuGluGlnArgMetIleThrValValTyrGlyProAspLeuValAsnIleSerHisLeuAsnLeuVal    120

GCTTTCCAAGAAGAAGTGGCCAAGGAATGGACAAATGAGGTTTTCAGTTTGGCAACAAACCTGCTGGCCCAAAACATGTCCAGGGATGCA    450
AlaPheGlnGluGluValAlaLysGluTrpThrAsnGluValPheSerLeuAlaThrAsnLeuLeuAlaGlnAsnMetSerArgAspAla    150

TTTCTGGAAAAAGCCTATACTAAACTTAAGCTGCAAGTCACTCCAGAAGGGCGTATTCCTCTCAAAAACATATATCGCTTGTTTTCAGCA    540
PheLeuGluLysAlaTyrThrLysLeuLysLeuGlnValThrProGluGlyArgIleProLeuLysAsnIleTyrArgLeuPheSerAla    180

GATCGGAAGCGAGTTGAAACTGCTTTAGAGGCTTGTAGTCTTCCATCTTCAAGGAATGATTCAATACCTCAAGAAGATTTCACTCCAGAA    630
AspArgLysArgValGluThrAlaLeuGluAlaCysSerLeuProSerSerArgAsnAspSerIleProGlnGluAspPheThrProGlu    210

GTGTACAGAGTTTTCCTCAACAACCTTTGCCCTCGACCTGAAATTGATAACATCTTTTCAGAATTTGGTGCAAAAAGCAAACCATATCTT    720
ValTyrArgValPheLeuAsnAsnLeuCysProArgProGluIleAspAsnIlePheSerGluPheGlyAlaLysSerLysProTyrLeu    240

ACCGTTGATCAGATGATGGATTTTATCAACCTTAAGCAGCGAGATCCTCGGCTTAATGAAATACTTTATCCACCTCTAAAACAAGAGCAA    810
ThrValAspGlnMetMetAspPheIleAsnLeuLysGlnArgAspProArgLeuAsnGluIleLeuTyrProProLeuLysGlnGluGln    270

GTCCAAGTATTGATTGAGAAGTATGAACCCAACAACAGCCTCGCCAGAAAAGGACAAATATCAGTGGATGGGTTCATGCGCTATCTGAGT    900
ValGlnValLeuIleGluLysTyrGluProAsnAsnSerLeuAlaArgLysGlyGlnIleSerValAspGlyPheMetArgTyrLeuSer    300

GGAGAAGAAAACGGAGTCGTTTCACCTGAGAAACTGGATTTGAATGAAGACATGTCTCAGCCCCTTTCTCACTATTTCATTAATTCCTCG    990
GlyGluGluAsnGlyValValSerProGluLysLeuAspLeuAsnGluAspMetSerGlnProLeuSerHisTyrPheIleAsnSerSer    330

CACAACACCTACCTCACAGCTGGCCAACTGGCTGGAAACTCCTCTGTTGAGATGTATCGCCAAGTGCTCCTGTCTGGTTGTCGCTGTGTG   1080
HisAsnThrTyrLeuThrAlaGlyGlnLeuAlaGlyAsnSerSerValGluMetTyrArgGlnValLeuLeuSerGlyCysArgCysVal    360
```

Additional file 4

```
GAGCTGGACTGCTGGAAGGGACGGACTGCAGAAGAGGAACCTGTCATCACCCATGGCTTCACCATGACAACTGAAATATCTTTCAAGGAA  1170
GluLeuAspCysTrpLysGlyArgThrAlaGluGluGluProValIleThrHisGlyPheThrMetThrThrGluIleSerPheLysGlu   390

GTGATAGAAGCAATTGCGGAGTGTGCATTTAAGACTTCACCTTTTCCAATTCTCCTTTCGTTTGAGAACCATGTGGATTCCCCAAAGCAG  1260
ValIleGluAlaIleAlaGluCysAlaPheLysThrSerProPheProIleLeuLeuSerPheGluAsnHisValAspSerProLysGln   420

CAAGCCAAGATGGCGGAGTACTGCCGACTGATCTTTGGGGATGCCCTTCTCATGGAGCCCCTGGAAAAATATCCACTGGAATCTGGAGTT  1350
GlnAlaLysMetAlaGluTyrCysArgLeuIlePheGlyAspAlaLeuLeuMetGluProLeuGluLysTyrProLeuGluSerGlyVal   450

CCTCTTCCAAGCCCTATGGATTTAATGTATAAAATTTTGGTGAAAAATAAGAAGAAATCACACAAGTCATCAGAAGGAAGCGGCAAAAAG  1440
ProLeuProSerProMetAspLeuMetTyrLysIleLeuValLysAsnLysLysLysSerHisLysSerSerGluGlySerGlyLysLys   480

AAGCTCTCAGAACAAGCCTCCAACACCTACAGTGACTCCTCCAGCATGTTCGAGCCCTCATCCCCAGGAGCCGGAGAAGCTGATACGGAA  1530
LysLeuSerGluGlnAlaSerAsnThrTyrSerAspSerSerSerMetPheGluProSerSerProGlyAlaGlyGluAlaAspThrGlu   510

AGTGACGACGACGATGATGATGATGACTGTAAAAAATCTTCAATGGATGAGGGGACTGCTGGAAGTGAGGCTATGGCCACAGAAGAAATG  1620
SerAspAspAspAspAspAspAspAspCysLysLysSerSerMetAspGluGlyThrAlaGlySerGluAlaMetAlaThrGluGluMet   540

TCTAATCTGGTGAACTATATTCAGCCAGTCAAGTTTGAGTCATTTGAAATTTCAAAAAAAAGAAATAAAAGTTTTGAAATGTCTTCCTTC  1710
SerAsnLeuValAsnTyrIleGlnProValLysPheGluSerPheGluIleSerLysLysArgAsnLysSerPheGluMetSerSerPhe   570

GTGGAAACCAAAGGACTTGAACAACTCACCAAGTCTCCAGTGGAATTTGTAGAATATAACAAAATGCAGCTTAGCAGGATATATCCAAAA  1800
ValGluThrLysGlyLeuGluGlnLeuThrLysSerProValGluPheValGluTyrAsnLysMetGlnLeuSerArgIleTyrProLys   600

GGAACACGTGTGGATTCATCCAACTATATGCCTCAGCTCTTCTGGAATGCAGGTTGTCAGATGGTGGCACTTAATTTCCAGACAATGGAC  1890
GlyThrArgValAspSerSerAsnTyrMetProGlnLeuPheTrpAsnAlaGlyCysGlnMetValAlaLeuAsnPheGlnThrMetAsp   630

CTGGCTATGCAAATAAATATGGGGATGTATGAATACAACGGGAAGAGTGGCTACAGATTGAAGCCAGAGTTCATGAGGAGGCCTGACAAG  1980
LeuAlaMetGlnIleAsnMetGlyMetTyrGluTyrAsnGlyLysSerGlyTyrArgLeuLysProGluPheMetArgArgProAspLys   660

CATTTTGATCCATTTACTGAAGGCATCGTAGATGGGATAGTGGCAAACACTTTGTCTGTTAAGATTATTTCAGGTCAGTTTCTTTCTGAT  2070
HisPheAspProPheThrGluGlyIleValAspGlyIleValAlaAsnThrLeuSerValLysIleIleSerGlyGlnPheLeuSerAsp   690

AAGAAAGTTGGGACTTACGTGGAAGTAGATATGTTTGGTTTGCCTGTGGATACAAGGAGGAAGGCATTTAAGACCAAAACATCCCAAGGA  2160
LysLysValGlyThrTyrValGluValAspMetPheGlyLeuProValAspThrArgArgLysAlaPheLysThrLysThrSerGlnGly   720
```

Additional file 4

AATGCTGTGAATCCTGTCTGGGAAGAAGAACCTATTGTGTTCAAAAAGGTGGTTCTTCCTACTCTGGCCTGTTTGAGAATAGCAGTTTAT 2250
AsnAlaValAsnProValTrpGluGluGluProIleValPheLysLysValValLeuProThrLeuAlaCysLeuArgIleAlaValTyr 750

GAAGAAGGAGGTAAATTCATTGGCCACCGTATCTTGCCAGTGCAAGCCATTCGGCCAGGCTATCACTATATCTGTCTAAGGAATGAAAGG 2340
GluGluGlyGlyLysPheIleGlyHisArgIleLeuProValGlnAlaIleArgProGlyTyrHisTyrIleCysLeuArgAsnGluArg 780

AACCAGCCTCTGACGCTGCCTGCTGTCTTTGTCTACATAGAAGTGAAAGACTATGTGCCAGACACATATGCAGATGTCATCGAAGCTTTA 2430
AsnGlnProLeuThrLeuProAlaValPheValTyrIleGluValLysAspTyrValProAspThrTyrAlaAspValIleGluAlaLeu 810

TCAAACCCAATCCGATATGTGAACCTGATGGAACAGAGAGCTAAGCAATTGGCTGCTTTGACACTGGAAGATGAAGAAGAAGTAAAGAAA 2520
SerAsnProIleArgTyrValAsnLeuMetGluGlnArgAlaLysGlnLeuAlaAlaLeuThrLeuGluAspGluGluGluValLysLys 840

GAGGCTGATCCTGGAGAAACACCATCAGAGGCTCCAAGTGAAGCGAGAACGACTCCAGCAGAAAATGGGGTGAATCACACTACAACCCTG 2610
GluAlaAspProGlyGluThrProSerGluAlaProSerGluAlaArgThrThrProAlaGluAsnGlyValAsnHisThrThrThrLeu 870

ACACCCAAGCCACCCTCCCAGGCTCTCCACAGCCAGCCAGCTCCAGGTTCTGTAAAGGCACCTGCCAAAACAGAAGATCTTATTCAGAGT 2700
ThrProLysProProSerGlnAlaLeuHisSerGlnProAlaProGlySerValLysAlaProAlaLysThrGluAspLeuIleGlnSer 900

GTCTTAACAGAAGTGGAAGCACAGACCATCGAAGAACTAAAGCAACAGAAATCGTTTGTGAAACTTCAAAAGAAACACTACAAAGAAATG 2790
ValLeuThrGluValGluAlaGlnThrIleGluGluLeuLysGlnGlnLysSerPheValLysLeuGlnLysLysHisTyrLysGluMet 930

AAAGACCTGGTTAAGAGACACCACAAGAAAACCACTGACCTTATCAAAGAACACACTACCAAGTATAATGAAATTCAGAATGACTACTTG 2880
LysAspLeuValLysArgHisHisLysLysThrThrAspLeuIleLysGluHisThrThrLysTyrAsnGluIleGlnAsnAspTyrLeu 960

AGAAGGAGAGCCGCTTTGGAAAAGTCCGCCAAAAAGGACAGTAAGAAAAAATCGGAACCCAGCAGCCCTGATCATGGTTCATCAACGATT 2970
ArgArgArgAlaAlaLeuGluLysSerAlaLysLysAspSerLysLysLysSerGluProSerSerProAspHisGlySerSerThrIle 990

GAGCAAGACCTCGCTGCCCTGGATGCTGAAATGACCCAAAAGTTAATAGACTTGAAGGACAAACAACAGCAGCAGCTGCTTAATCTTCGG 3060
GluGlnAspLeuAlaAlaLeuAspAlaGluMetThrGlnLysLeuIleAspLeuLysAspLysGlnGlnGlnGlnLeuLeuAsnLeuArg 1020

CAAGAACAGTATTATAGTGAAAAATACCAGAAGCGAGAACATATTAAACTGCTTATTCAAAAGTTGACGGATGTCGCAGAAGAGTGTCAG 3150
GlnGluGlnTyrTyrSerGluLysTyrGlnLysArgGluHisIleLysLeuLeuIleGlnLysLeuThrAspValAlaGluGluCysGln 1050

AACAATCAGTTAAAG**ATA**CTCAAAGAAATCTGTGAG**ATA**GAAAAGAAAGAATTAAAGAAG**ATA**ATGGATAAAAAGAGGCAGGAGAAGATA 3240
AsnAsnGlnLeuLys**Ile**LeuLysGluIleCysGlu**Ile**GluLysLysGluLeuLysLys**Ile**MetAspLysLysArgGlnGluLysIle 1080

Additional file 4

```
ACAGAAGCTAAATCCAAAGACAAAAGTCAGATGGAAGAGGAGAAGACAGAGATGATCCGGTCATATATCCAGGAAGTGGTGCAGTATATC        3330
ThrGluAlaLysSerLysAspLysSerGlnMetGluGluGluLysThrGluMetIleArgSerTyrIleGlnGluValValGlnTyrIle        1110

AAGAGGCTAGAAGAAGCGCAAAGTAAACGGCAAGAAAAACTCGTAGAGAAACACAAGGAAATACGTCAGCAGATCCTGGATGAAAAGCCC        3420
LysArgLeuGluGluAlaGlnSerLysArgGlnGluLysLeuValGluLysHisLysGluIleArgGlnGlnIleLeuAspGluLysPro        1140

AAGGGGGAAGGTTCCTCCTCATTCTTGTCGGAAACTTGCCATGAGGATCCCTCTGTTTCCCCCAACTTTACTCCCCCCAACCCTCAAGCT        3510
LysGlyGluGlySerSerSerPheLeuSerGluThrCysHisGluAspProSerValSerProAsnPheThrProProAsnProGlnAla        1170

CTCAAGTGGTGAtctagattgcggccgctcgagcatgcatctagagggccctattctatagtgtcacctaaatgctagagctcgctgatc        1270
LeuLysTrp *                                                                                       1173
```

Supplemental figures

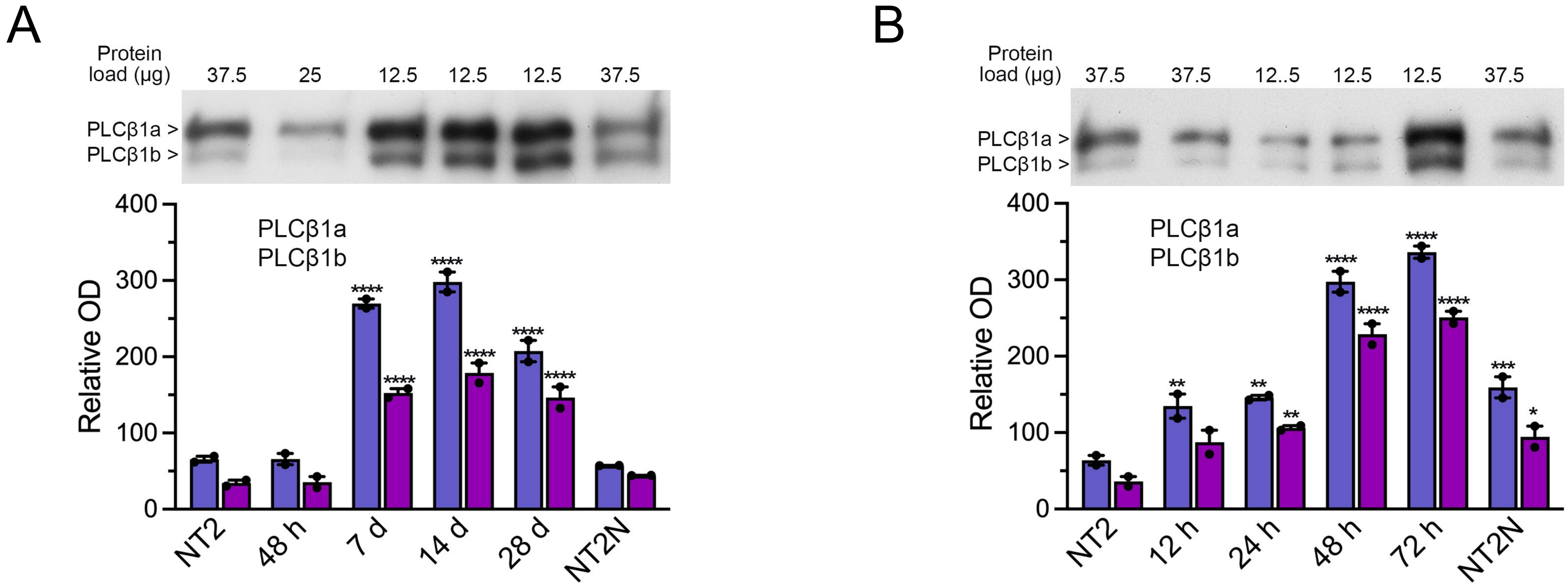

Figure S1. *Analysis of PLCβ1 levels during neuronal differentiation of NT2 progenitors*. **A-** Western blot analysis of the time course of PLCβ1 expression during RA-induced neuronal differentiation of NT2 cells. **B,** Western blot analysis of the time course of PLCβ1 expression during AraC-induced neuronal differentiation of NT2 cells. Immunoblots were performed in whole homogenates adjusting total protein amounts to allow correct separation and visualization of immunoreactive bands corresponding to PLCβ1a and PLCβ1b splice variants. Bar graphs depict relative optical density (OD) expressed as percent of PLCβ1a and PLCβ1b signals normalized to the sum of PLCβ1a and PLCβ1b in NT2 progenitors. Two-way ANOVA with post-treatment time and splice variant (PLCβ1a and PLCβ1b) as main factors followed by Bonferroni's *post-hoc* test was performed. *$p < 0.05$, **$p < 0.01$, ***$p < 0.001$, ****$p < 0.0001$

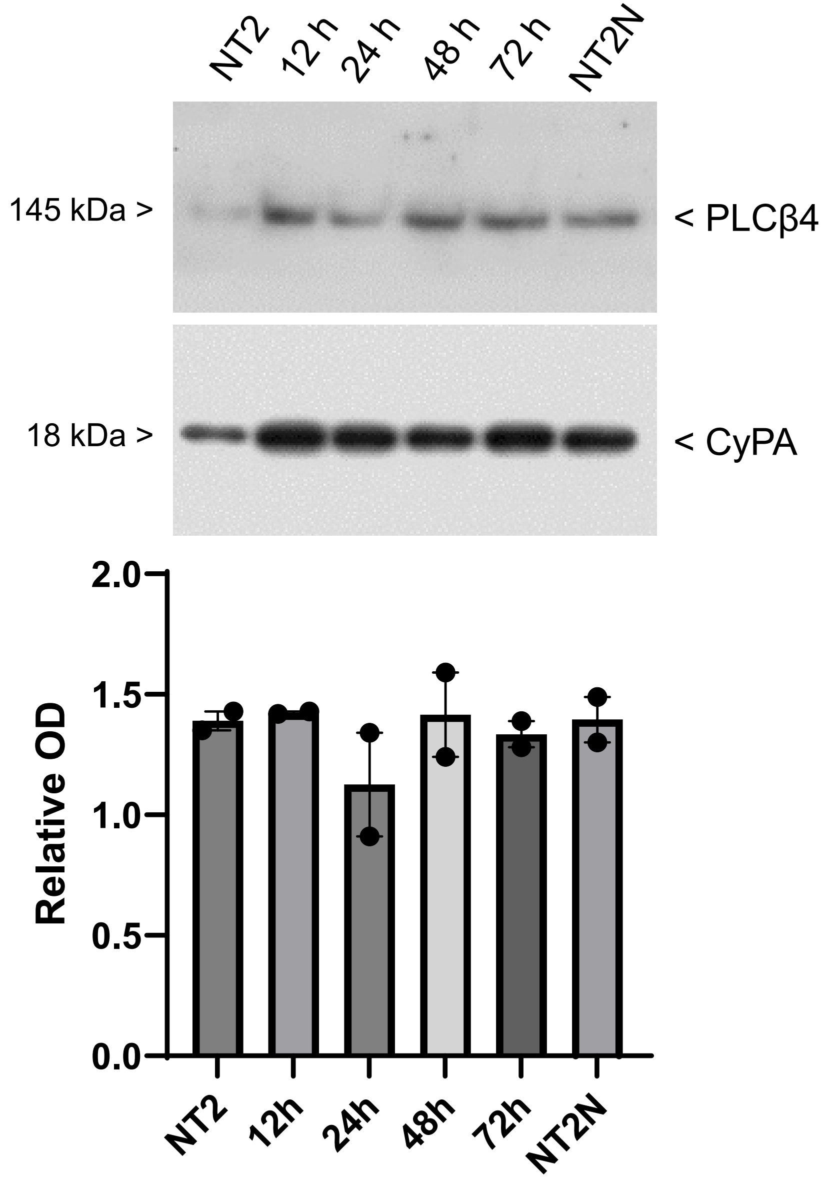

Figure S2. Immunoblots of PLCβ4 on whole homogenates of NT2 progenitors. AraC-treated NT2 cells were analyzed at 12 h, 24 h, 48 h, 72 h and 6 days (NT2N) after the onset of AraC-induction. Equal amounts of total protein (12 µg) were loaded. The ordinate axis in the graph shows relative optical density (OD) of immunoreactive bands expressed as percent of that found in undifferentiated NT2 progenitors. Data were statistically analyzed repeated measures one-way ANOVA , followed by Tukey's *post-hoc* test. No significant differences between the different samples with respect to NT2 progenitors were observed. Data are mean ± SEM (n=2).

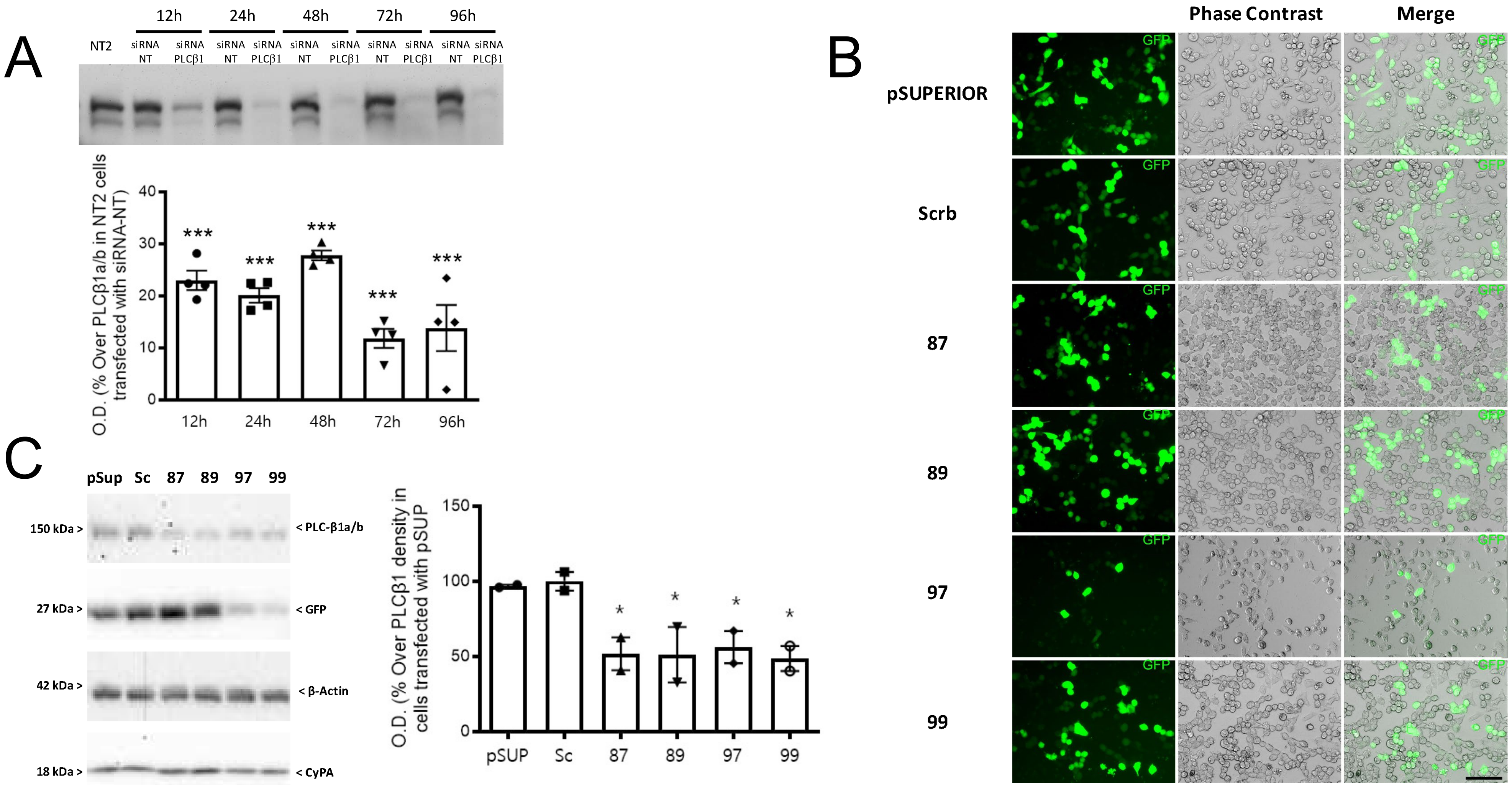

Figure S3. Validation of the silencing efficiency of siRNAs and shRNAs. **A.** Immunoreactivity for PLCβ1 expression in PLCβ1 siRNA-treated NT2 cells relative to that in non-targeting scrambled siRNA-treated ones at different post-transfection times (n=4). **B**. Efficiency of transfection was detected by GFP expression using fluorescence microscopy. NT2 cells were transfected with pSUPERIOR-GFP plasmid containing coding sequences for non-targeting scramble (Scr shRNA) or different shRNAs (1-4) targeting PLCβ1 transcripts. **C**. Western blot analysis of PLCβ1, GFP, β-Actin and cyclophyllin (CyPA) in NT2 cell homogenates (30 µg protein/lane) after 48h of post transfection with Scr shRNA or shRNAs 1-4. Bar graph depicts relative expression of PLCβ1 under the different conditions.

Figure S4A-F. Triple-immunofluorescence against PLCβ1, βIII-Tub and GFP combined with Hoechst's chromatin staining in NT2 cells transfected with either scrambled shRNA (**A, D**) or PLCβ1 shRNA mix (**B, E**) and treated with AraC for 72 h. PLCβ1 and βIII-Tub are pseudo-colored red for better visualization, but secondary antibodies conjugated with non-overlapping fluorochromes were used. Similarly to non-transfected NT2 progenitors (asterisks in **A,D**), Cells transfected with scramble shRNA (GFP-positive; arrowheads in A,D) acquired neuronal morphology and expressed both PLCβ1 and βIII-Tub. In contrast, NT2 cells transfected with the PLCβ1 shRNA mix failed to differentiate and did not express PLCβ1 or βIII-Tub (arrowheads in **B,E**), whereas non-transfected ones were unaffected (asterisks in B,E). Scale bar in E = 40 µm (applies to all micrographs). Bar Graphs showing the PLCβ1/GFP (**C**) and βIII-Tub/GFP (**F**) immunofluorescence signal intensity ratios in NT2 cells transfected with either the empty plasmid (mock), a scrambled shRNA or a PLCβ1 shRNA mix. Ratio values in each condition were normalized to the average ratio in mock-transfected condition. Repeated measures one-way ANOVA followed by Tukey's post-hoc test (**p* < 0.05; ***p* < 0.005). Data are mean ± SEM (n=4).

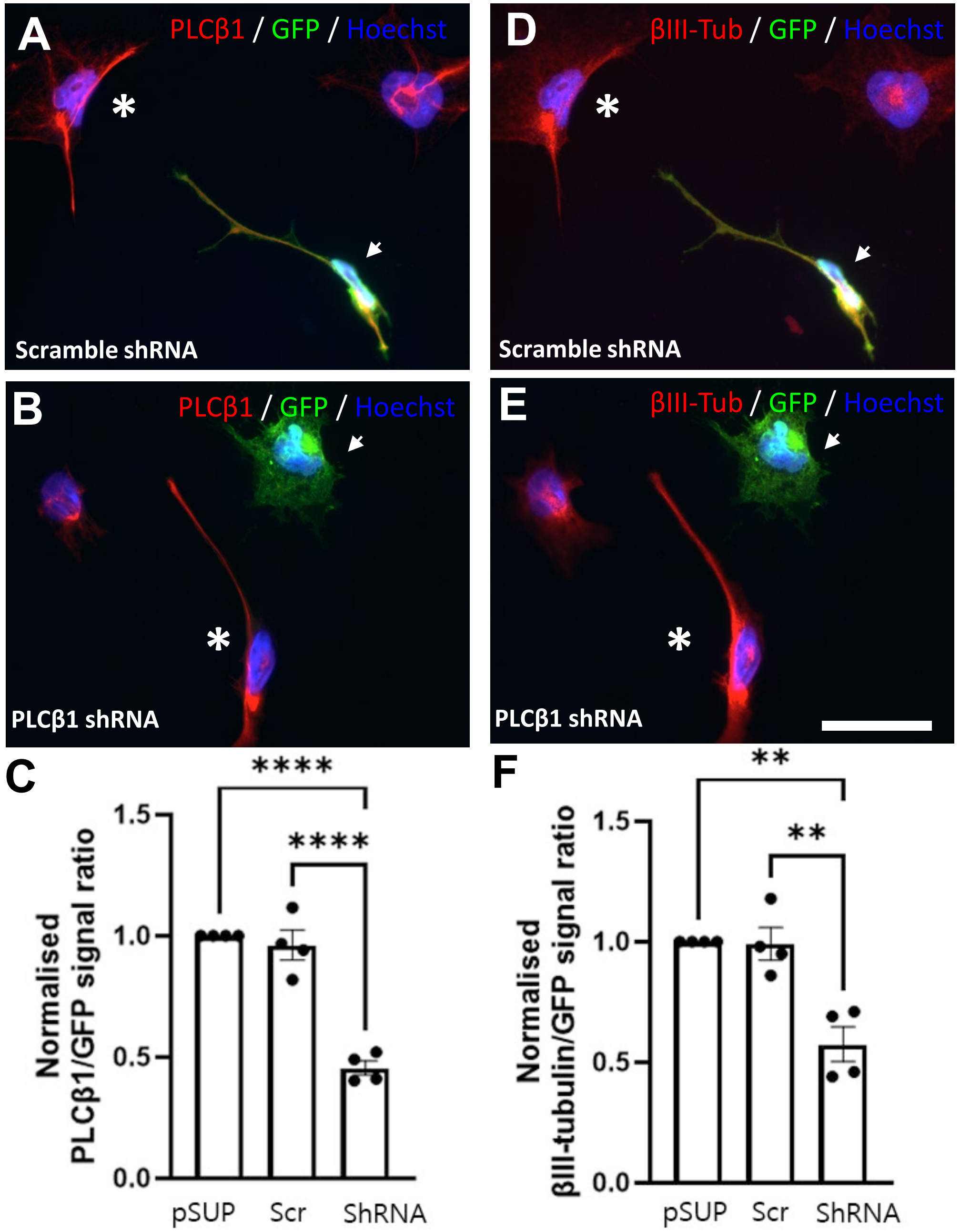

Figure S4G. Rat hippocampal astrocyte immunofluorescence study. Cell groups were either treated with rat PLCβ1 siRNA for downregulation (PLCβ1-) or treated with astrocyte growth medium only (Ctrl). Astrocytes were fixed after 24 h, 48 h, and 72 h by incubating with 3.7% paraformaldehyde/DPBS solution. All groups of cell were immunostained by antibodies against NF-H (CruzFluor™ 647, equivalent to Alexa Fluor™ 647), PLCβ1 (Alexa Fluor™ 488), and DAPI (nucleus). Cell deaths and low confluence were observed in all PLCβ1-downregulated groups, and morphology of these groups of cell are skinnier than their correspionding cells in control groups. NF-H intensity increases as time goes in control groups, especially in nucleus. LUT Intensity: Brightfield (4000 - 6500), NF-H (150 – 750), PLCβ1 (150 - 750), DAPI (750 - 6000). Scalebar: 50 µm (0.27 µm/pixel).

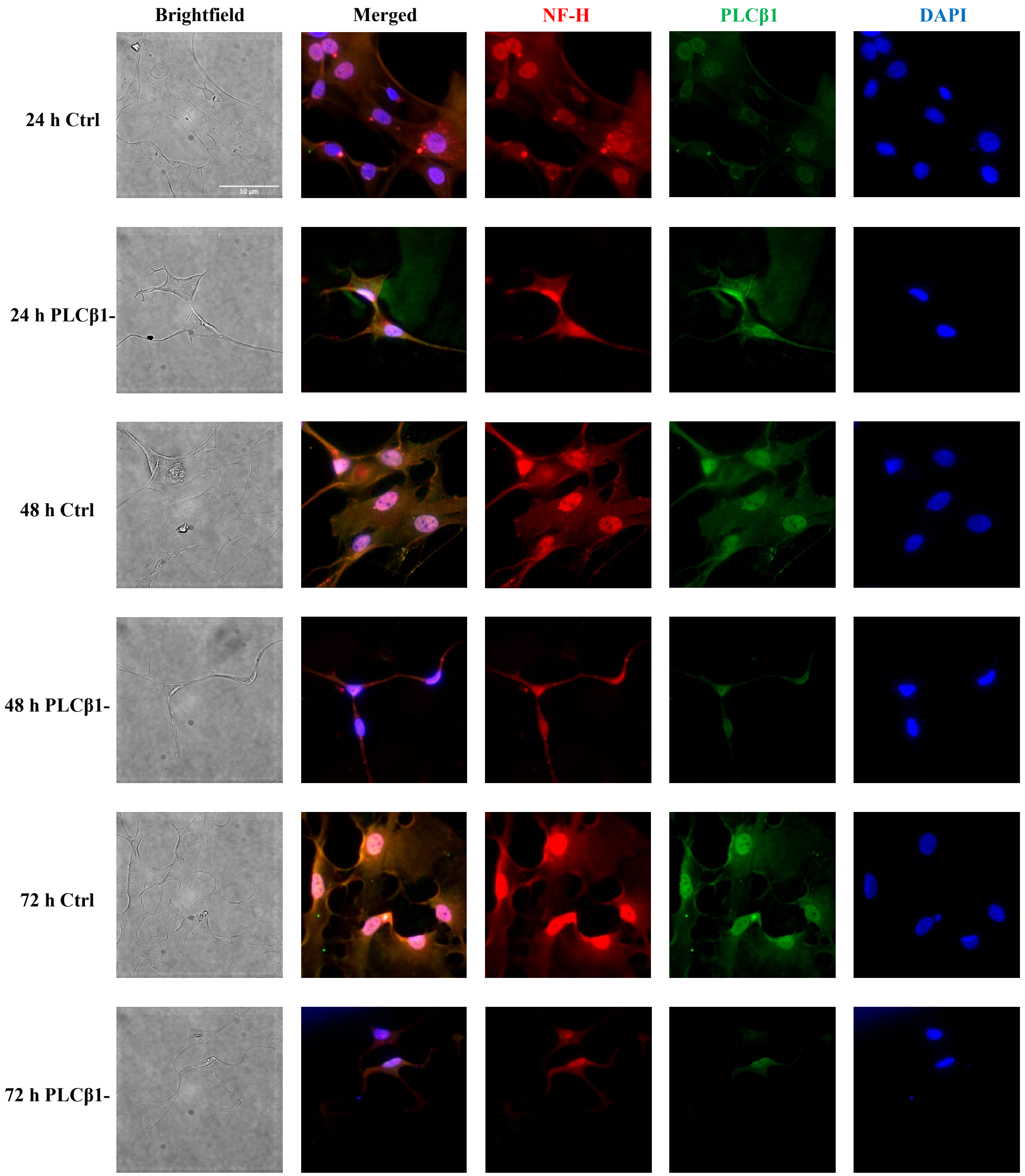

Figure S5. 72 h ERK 1/2 levels in PC12 cells which have different treatments. For ERK 1/2 localization study, cells transfected by pcDNA PLCβ1 or rat PLCβ1 siRNA, and control cells were immunostained by primary antibody ERK 1/2 Antibody (H-72) (1:200 dilution, Santa Cruz Biotechnologies, sc-292838), and then secondary antibody Chicken anti-Rabbit IgG (H+L) Cross-Adsorbed Secondary Antibody, Alexa Fluor™ 488 (#A-21441, Thermo Fisher Scientific, 1:400 dilution). Unbound antibodies were washed away by DPBS. Box plot shows all data points, min to max. Unpaired t test was calculated and shown on the graph: $*p < 0.05$, $***p < 0.001$, $****p < 0.0001$.

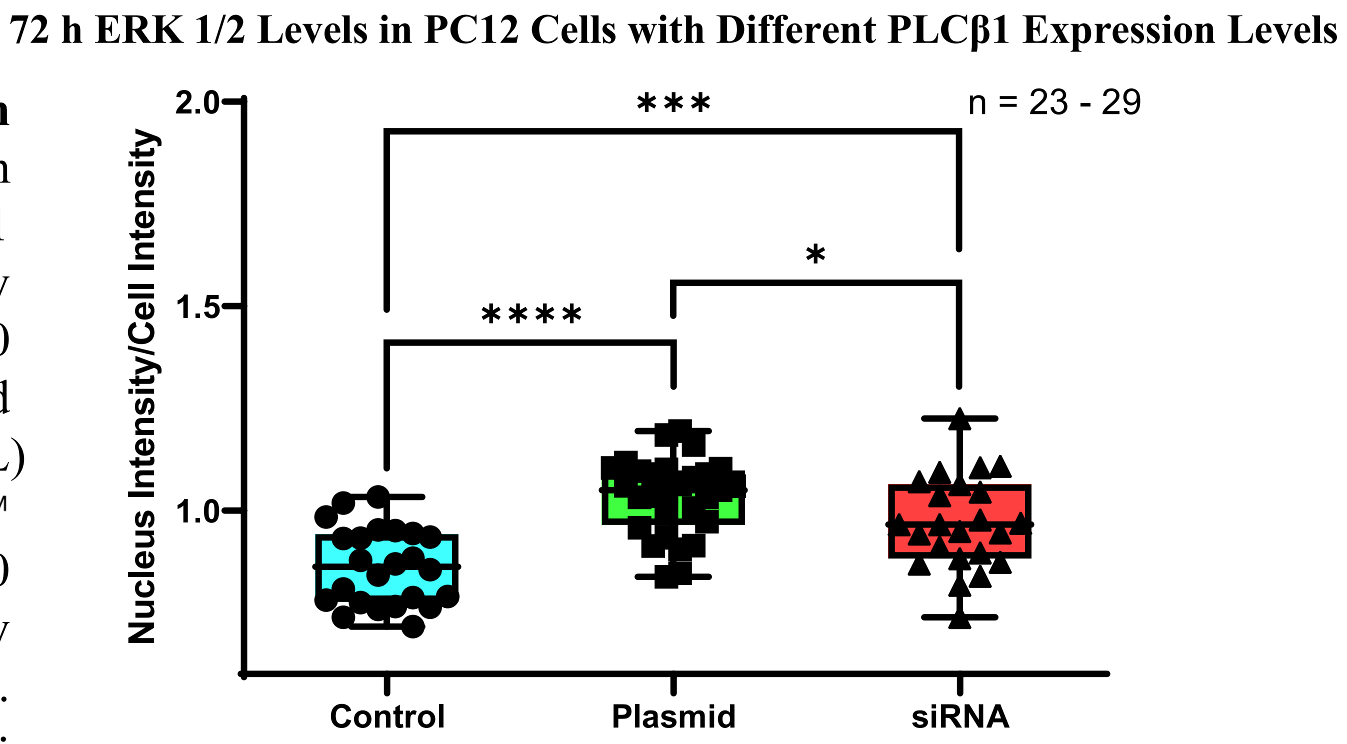

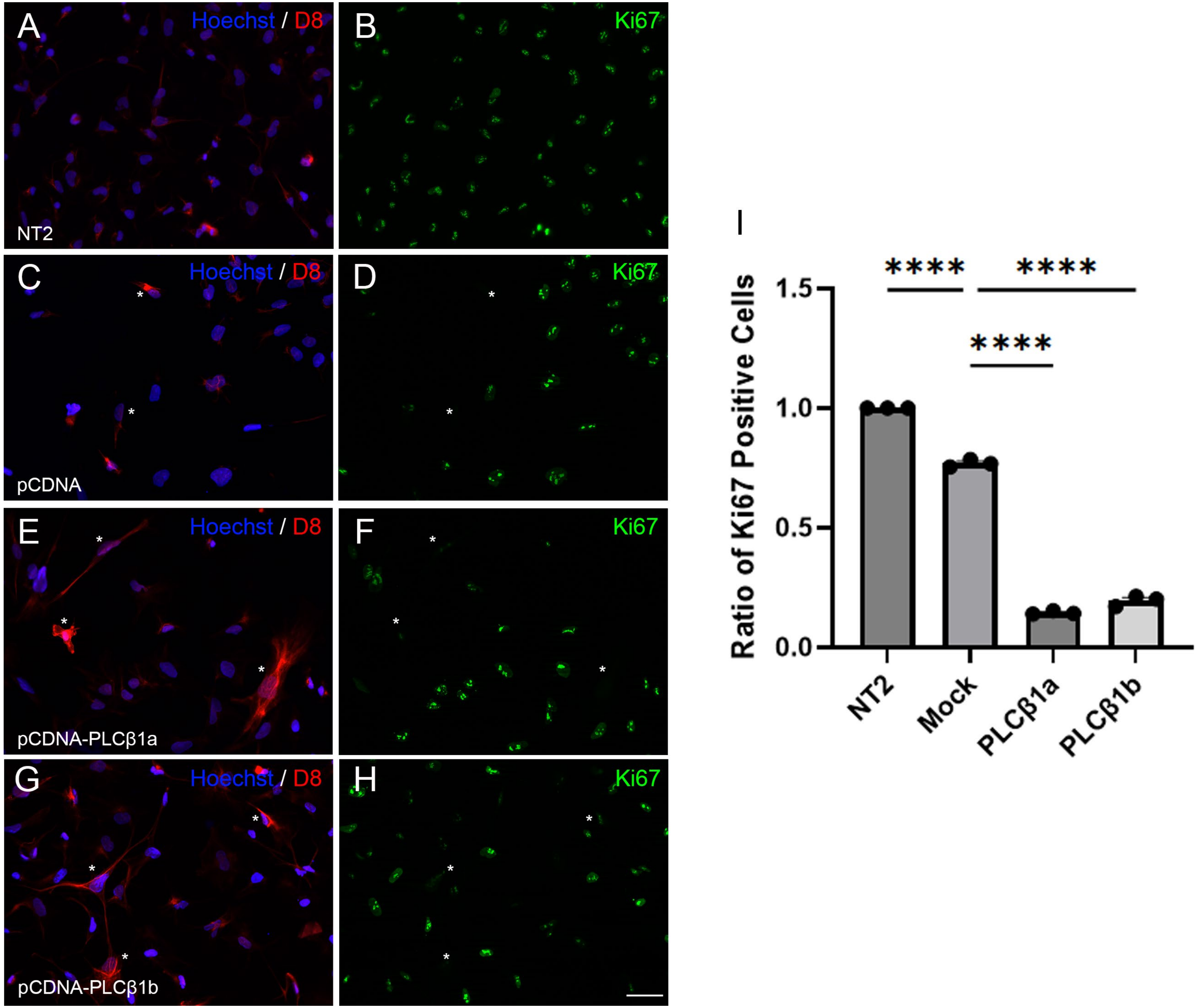

Figure S6. Effect of overexpression of PLCβ1a and PLCβ1b on Ki67 expression. **A-H,** NT2 progenitor cells, NT2 progenitor cells transfected with empty pCDNA, pcDNA-PLCb1a or pcDNA-PLCb1b, fixed 72 h after transfection and processed for double immunofluorescence against PLCβ1 (D-8, red) and ki67 (green), combined with Hoechst's chromatin staining (blue). Scale bar = 50 µm (applies to all micrographs). **I,** Graph showing the percentage of NT2 positive ki67 cells in cultures of NT2 progenitors either non-transfected or transfected with empty pcDNA, pcDNA-PLCb1a, pcDNA-PLCb1b, pcDNA-PLCb1a-M2b or pcDNA-PLCb1b-M2b. All data are mean ± SEM (n=3). All data were analyzed by one-way ANOVA followed by *post-hoc* Tukey's multi-comparison test (***$p < 0.001$)

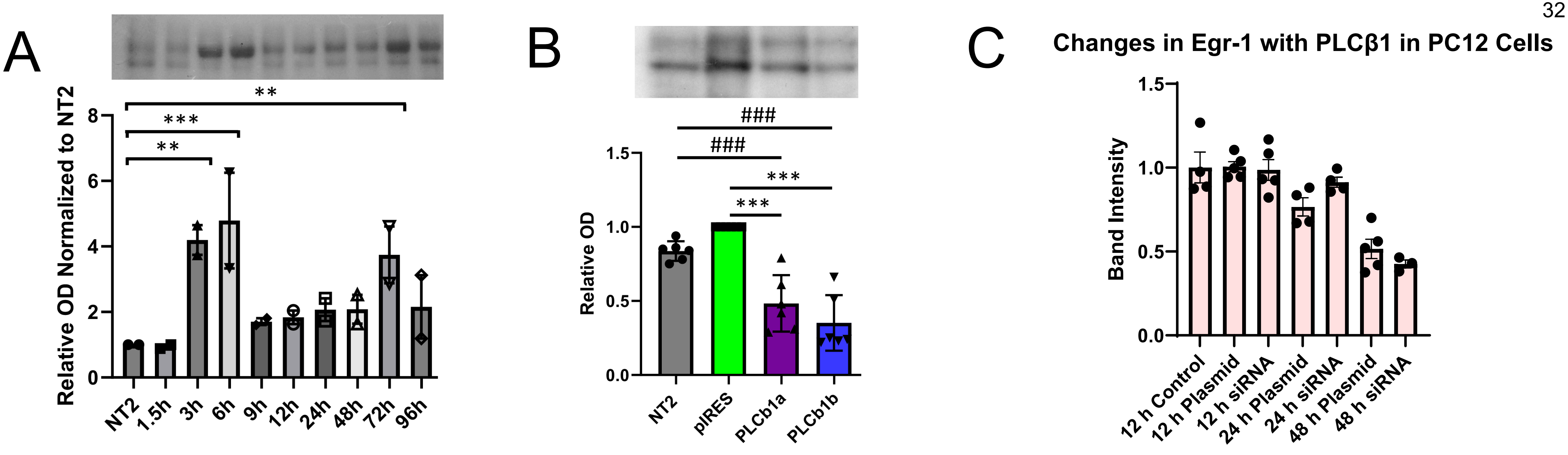

Figure S7. *Western blot analysis of Egr-1 expression during AraC-induced NT2 cell differentiation and impact of PLCβ1 over-expression*. **A,** Western blot analysis of Egr-1 expression during AraC-induced differentiation. Immunoblots were performed in whole homogenates loading 40 µg of total protein. Relative optical density (OD) was normalized to the signal in NT2 progenitor cells. One-way ANOVA followed by Bonferroni's *post-hoc* test (**$p < 0.01$, ***$p < 0.001$) . **B**, Western blot analysis of Egr-1 expression in non-transfected NT2 cells and 72 h after transfection with the empty pIRES plasmid, pIRES-PLCβ1a or pIRES-PLCβ1b. One-way ANOVA followed by Bonferroni's *post-hoc* test. All data represent means ± SEM (n=3) normalized to pIRES ($^{###}p < 0.001$, ***$p < 0.001$) **C,** Western blot analysis of Egr-1 expression in PC12 cells, PC12 cells were transfected either with eGFP-PLCβ1 vector, coding for rat PLCβ1 fused to enhanced green fluorescent protein (eGFP), or with siRNAs targeting rat PLCβ1. Data are mean ± SD (n = 4).

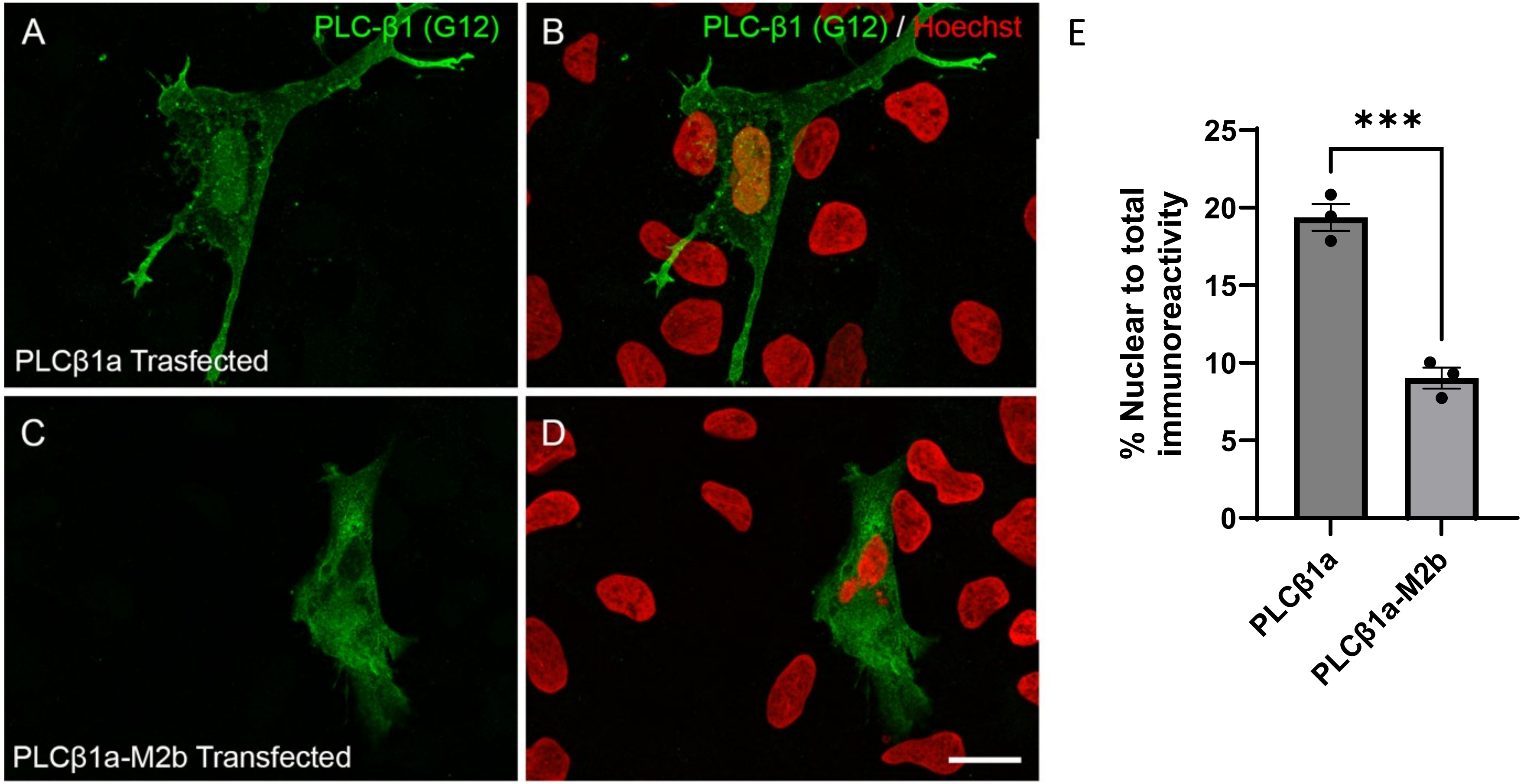

Figure S8. A-D, Confocal microscopy images of NT2 cells transiently transfected with the wild-type PLCβ1a (A-B) or with the nuclear localization mutant PLCβ1a-M2b (C-D) and immunostained with anti-PLCβ1a (G12 goat polyclonal antibody, green) in combination with Hoechst's chromatin staining (pseudo-colored red). Scale bare = 20 µm (applies to A-D). **E,** Bar graph showing the percent nuclear to total cellular PLCβ1a-immunofluorescence (integrated optical density) in PLCβ1a and PLCβ1a-M2b transfected NT2 cells. Quantification was performed on confocal optical sections from a total of 60 individual cells (20 cells per condition and replicate) randomly sampled. Data are mean ± SEM from three independent experiments. Two-tailed Unpaired t-test (***$p < 0.001$).

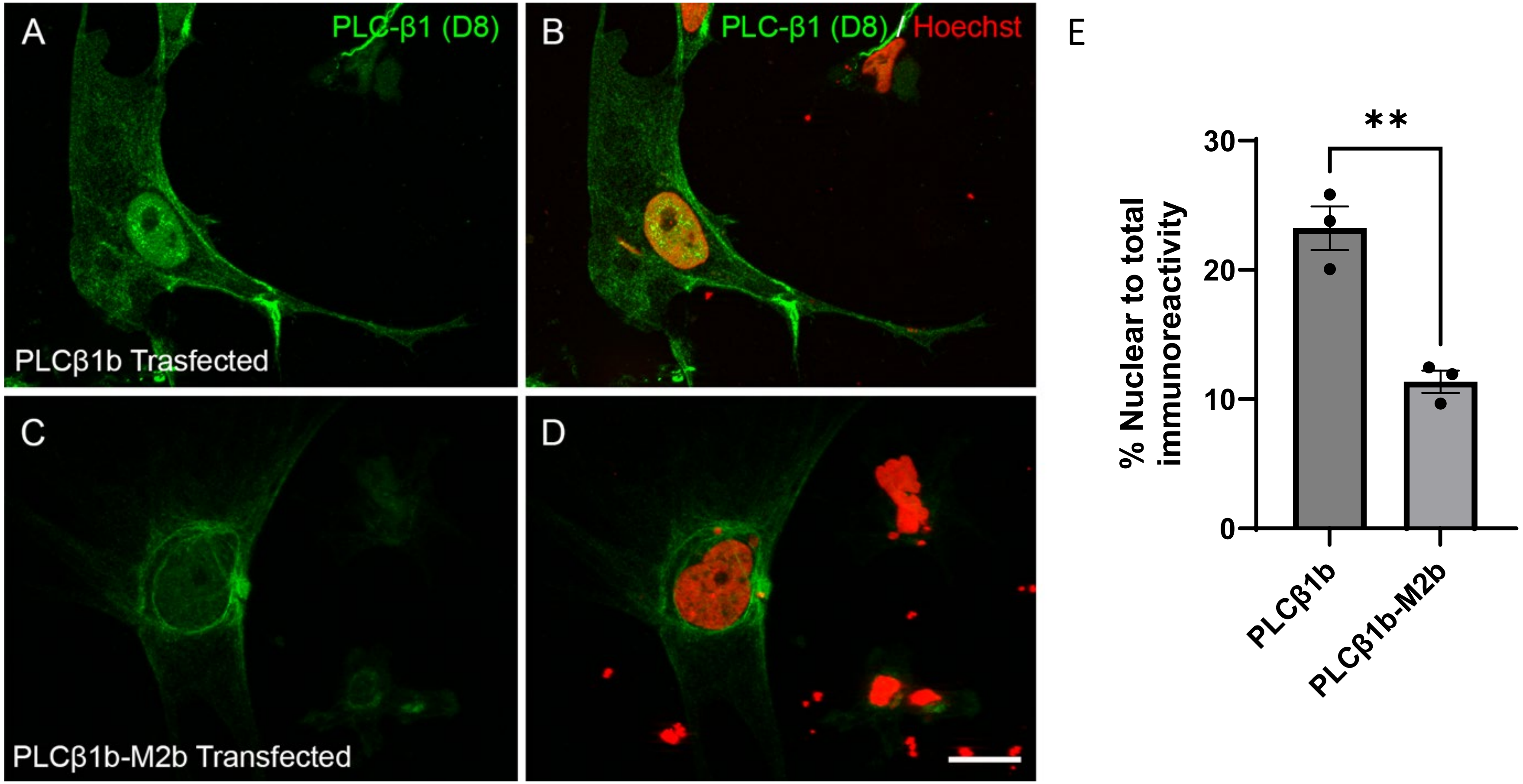

Figure S9. A-D, Confocal microscopy images of NT2 cells transiently transfected with the wild-type PLCβ1b (A-B) or with the nuclear localization mutant PLCβ1b-M2b (C-D) and immunostained with anti-PLCβ1 (D8 mouse monoclonal antibody, green) in combination with Hoechst's chromatin staining (pseudo-colored red). Scale bare = 20 µm (applies to A-D). **E,** Bar graph showing the percent nuclear to total cellular PLCβ1-immunofluorescence (integrated optical density) in PLCβ1b and PLCβ1b-M2b transfected NT2 cells. Quantification was performed on confocal optical sections from a total of 60 individual cells (20 cells per condition and replicate) randomly sampled. Data are mean ± SEM from three independent experiments. Two-tailed Unpaired t-test (***p* < 0.01).

Figure S10. Localization of Egr-1 and TRBP in HEK293 cells. Cell groups were either treated with tetracycline for PLCβ1 upregulation (PLCβ1+) or treated with complete HEK-293 cell medium only (Ctrl). Cells were fixed after 1h, 24 h, 60 h, and 78.5 h by incubating with 3.7% paraformaldehyde/DPBS solution. All cells were immunostained by antibodies against TRBP (Alexa Fluor® 488), Egr-1 (Alexa Fluor® 647), and DAPI (nucleus). **A** shows selected immunofluorescence and brightfield images captured from all cell groups. We observe PLCβ1-upregulation starting from 60 h and significant at 78.5 h. **B** shows the localization study of Egr-1 and **C** shows the TRBP localization study based on the captured images. PLCβ1+ group's cells have higher Egr-1 nuclear intensity than control groups starting from 60 h. Differences between PLCβ1+ and control groups on TRBP nuclear localization is not significant except 60. Box plot shows all data points, min to max. Significance results of unpaired t test: ns ($P > 0.05$), * ($P \leq 0.05$), ** ($P \leq 0.01$), *** ($P \leq 0.001$), and **** ($P \leq 0.0001$). Scalebar: 50µm (0.27µm/pixel).

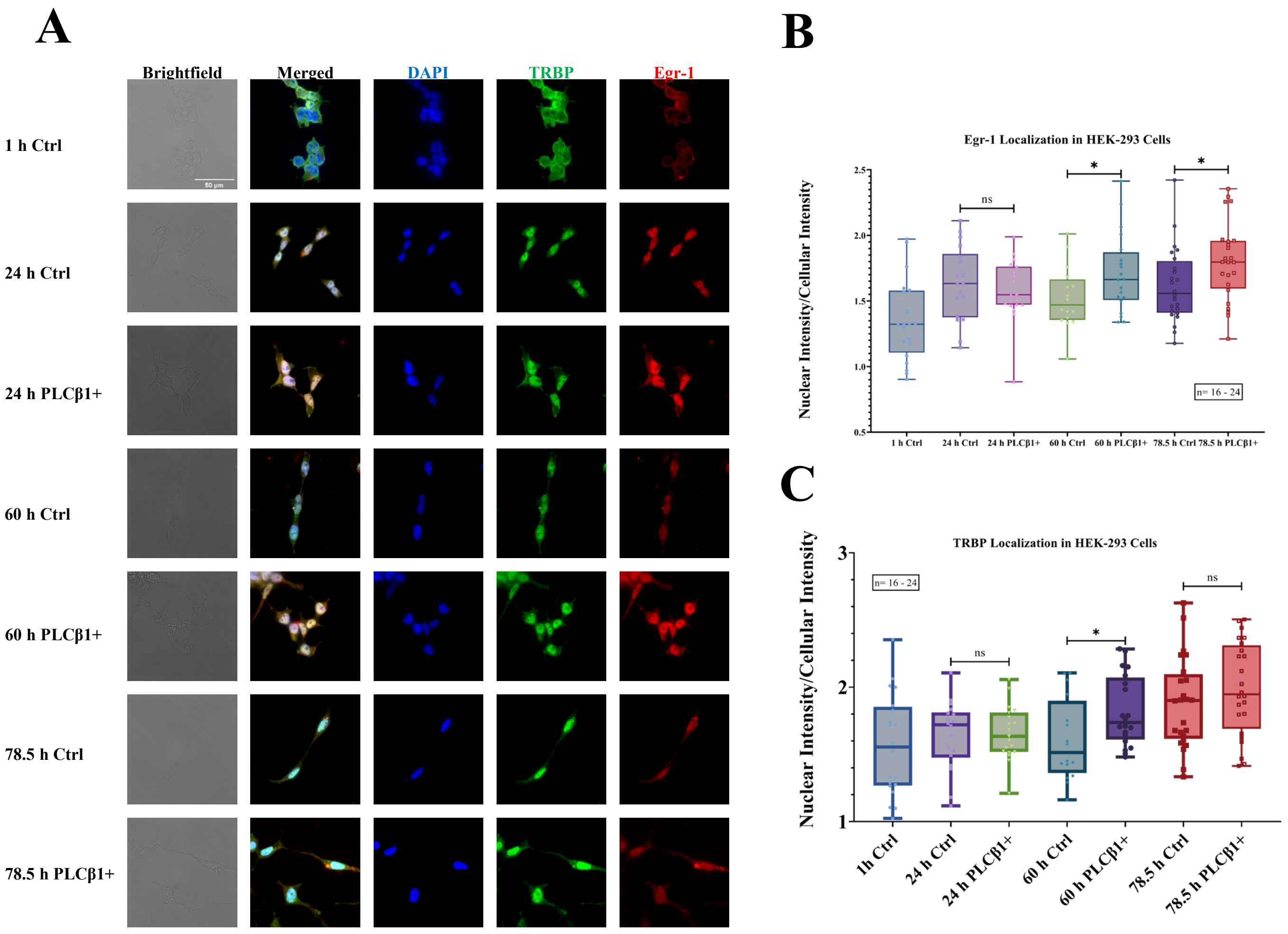

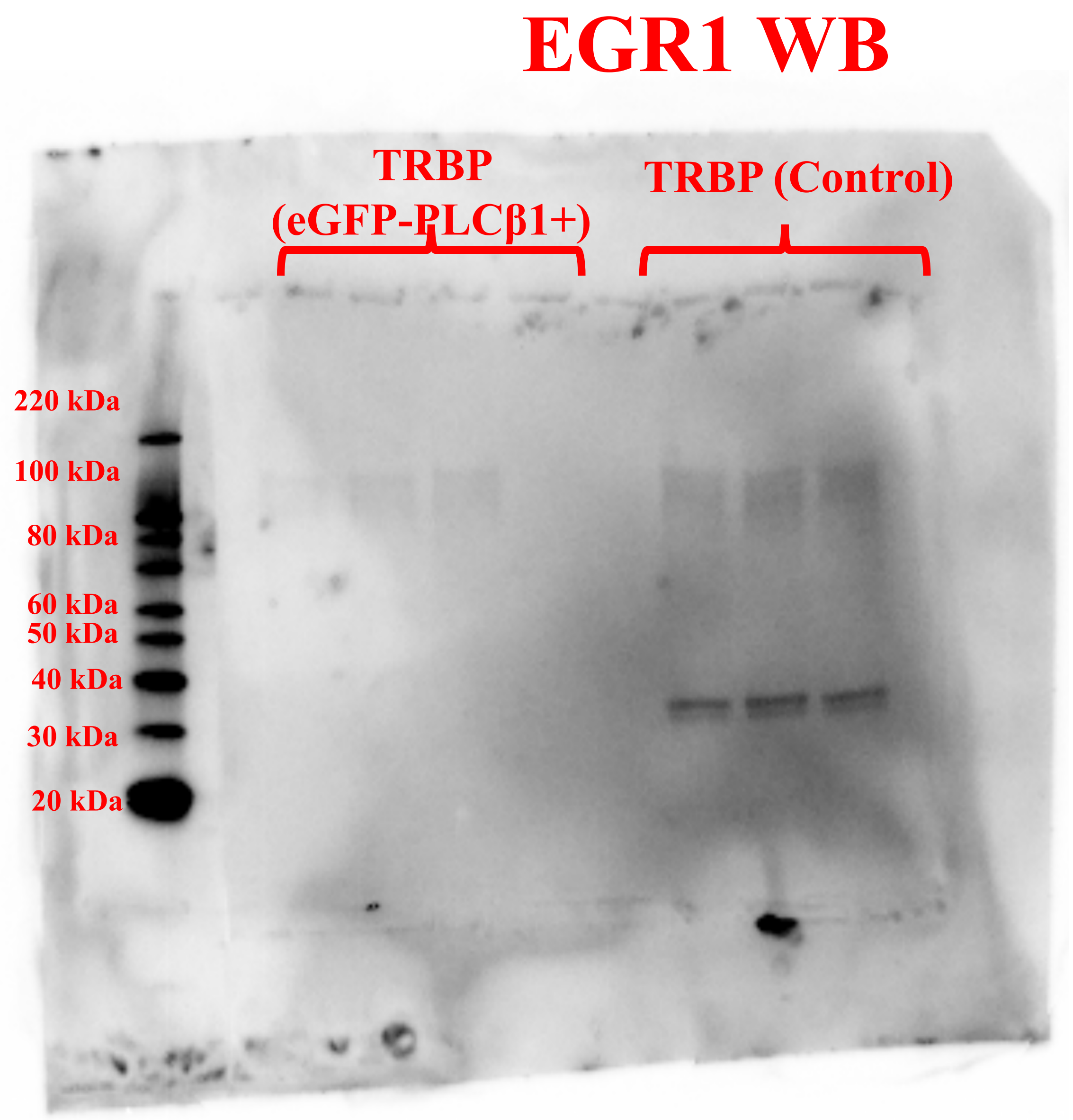

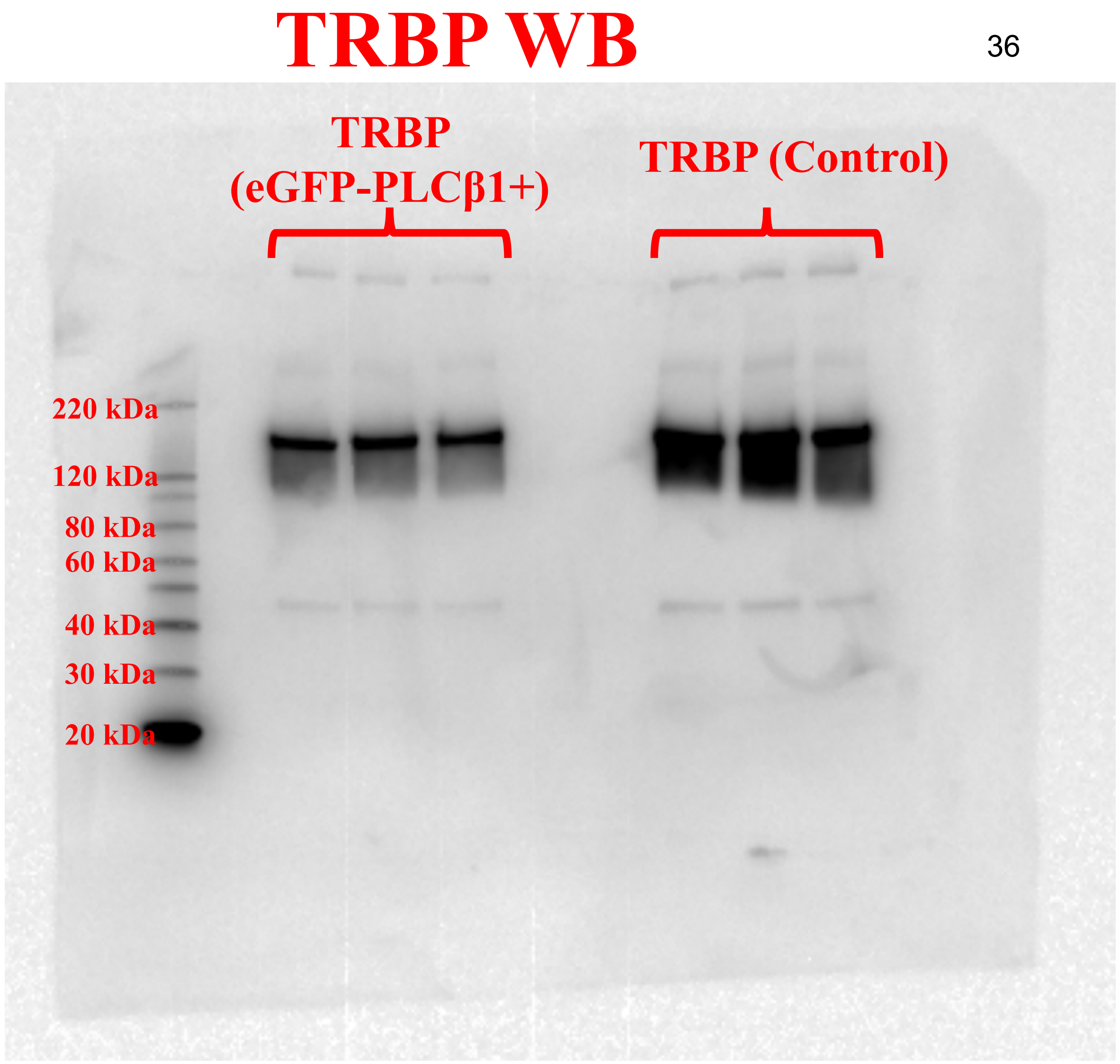

Figure S10D. *Full blot for* ***Fig. 6W*** *showing EGR1 and TRBP pull-downs in mock and eGFP-PLCb1 transfected PC12 cells.* Egr-1 migrates ~85 kDa, and TRBP migrates ~45 kDa. The high MW bands in the TRBP blot were analyzed by mass spectrometry and found to be mouse immunoglobulins.

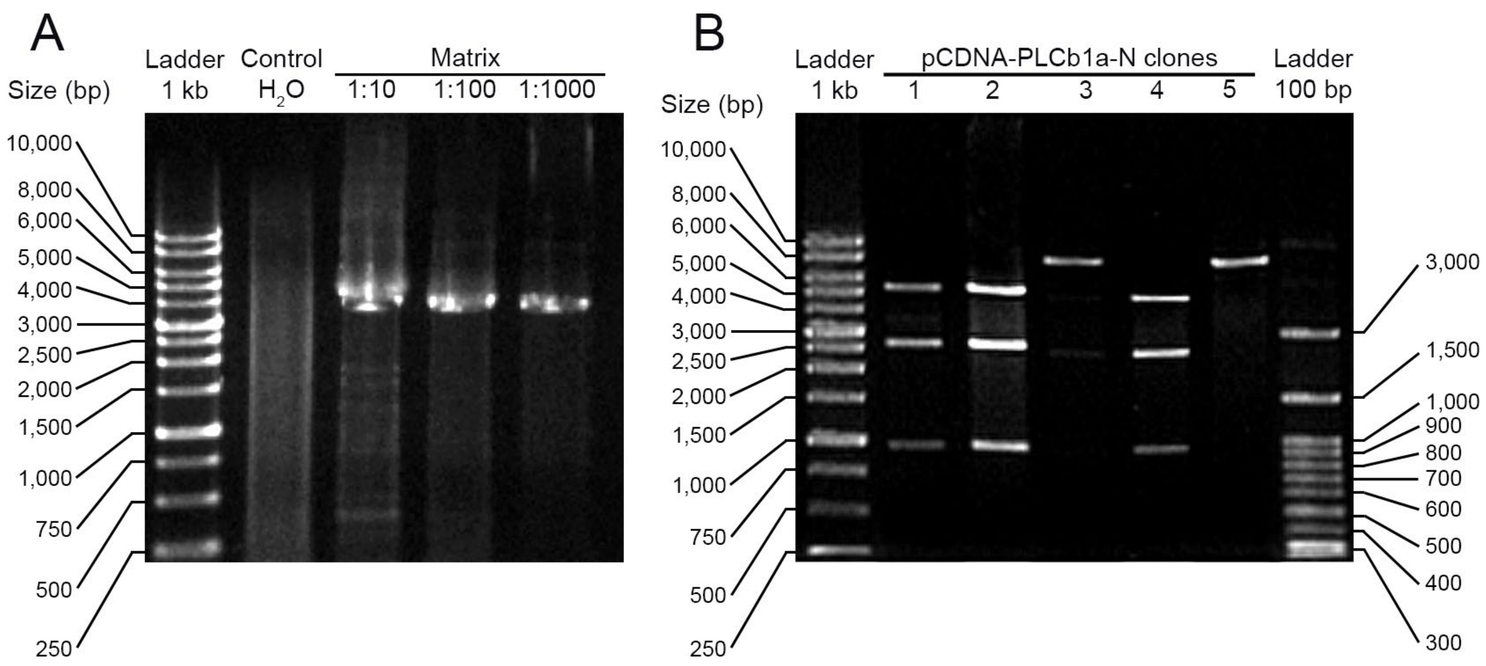

Figure S11. Agarose gel electrophoresis of PCR products and transformants. **A**, Agarose gel electrophoresis analysis of PCR products containing most of the open reading frame for human PLCβ1a. PCR amplification was performed using the commercial plasmid pCMV6-XL6-PLCb1a as a matrix. **B**, EcoRV/BglII restriction analysis of bacterial clones of the pDNA-PLCb1a-N plasmid. Three positive clones of 5 were obtained with DNA fragments consistent with the expected sizes of 5492, 2691 and 958 bp.

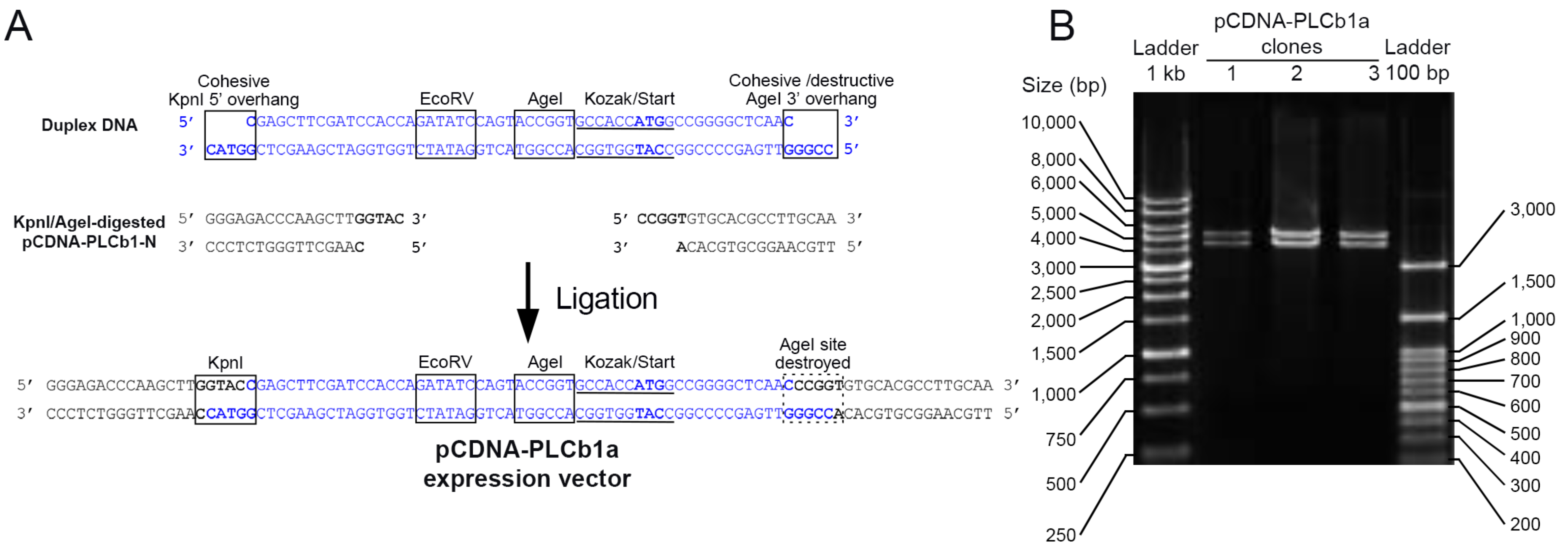

Figure S12. A, Schema of the strategy to reconstruct a functional mammalian expression pCDNA-PLCb1a vector by ligation of a synthtetic dúplex DNA in the digested N-terminal cloning vector pCDNA-PlCb1a-N. **B**, EcoRI/MluI restriction analysis of bacterial clones of the pDNA-PLCb1a plasmid. Three positive clones with DNA fragments consistent with the expected sizes of 4906 and 4197 bp were obtained.

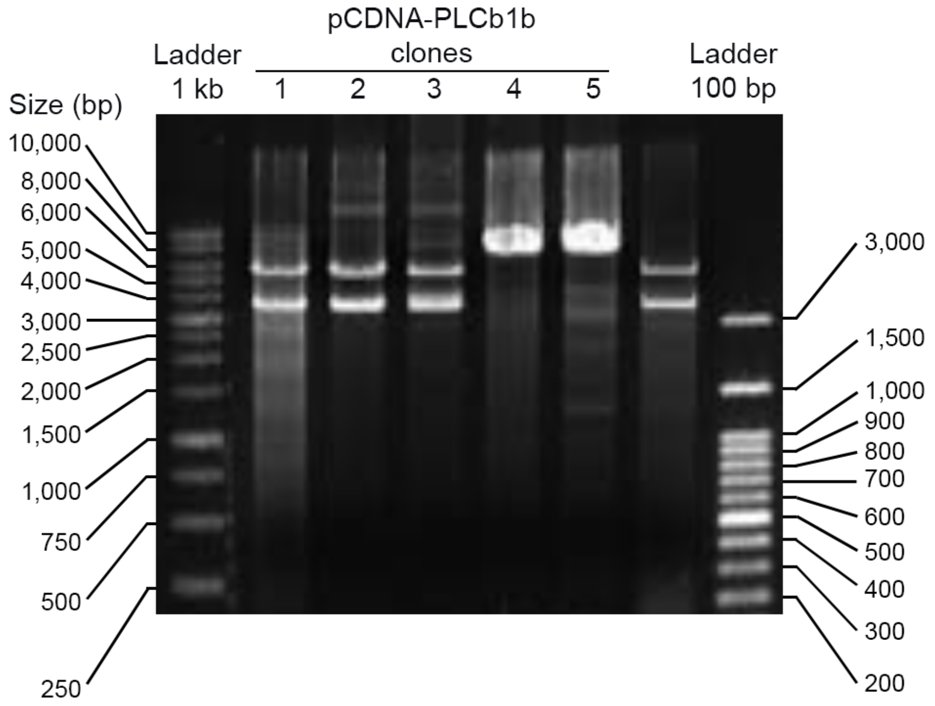

Figure S13. EcoRI/MluI restriction analysis of bacterial clones of the pDNA-PLCb1b plasmid. Four positive clones of 5 with DNA fragments consistent with the expected sizes of 5465 and 3486 bp were obtained

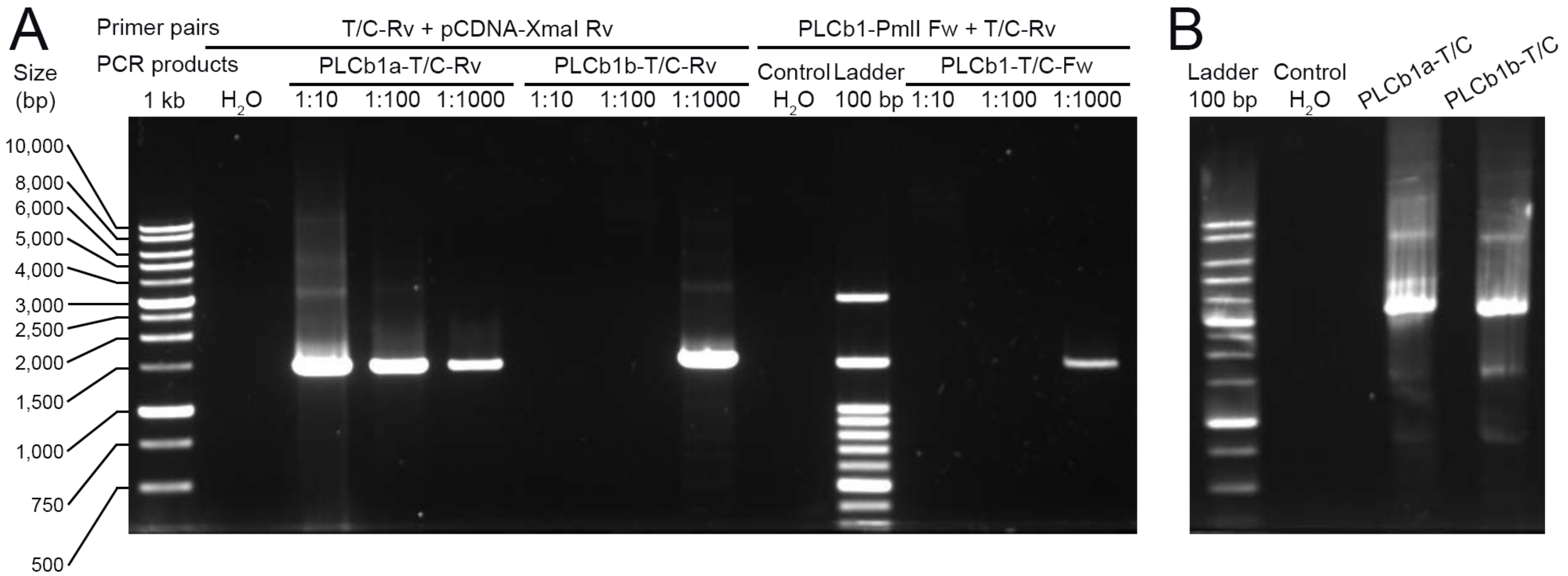

Figure S14. Agarose gel electrophoresis of PCR products used to reverse a C>T single nucleotide mutation by site-directed mutagénesis using an strategy based on overlap extension PCR. **A**, PCR products resulting form the first PCR. PLCb1a-T/C-Rv, PLCb1b-T/C-Rv and PLCb1-T/C-Fw were obtained using the primer pairs indicated above and using pCDNA-PLCb1a (PLCb1a-T/C-Rv and PLCb1-T/C-Fw) or pCDNA-PLCb1a (PLCb1b-T/C-Rv) as matrix at 1:10, 1:100 or 1:1000 dilution. **B**, PLCb1a-T/C and PLCb1b-T/C amplicons resulted from PCR overlap by combining PLCb1-T/C-Fw with PLCb1a-T/C-Rv and PLCb1b-T/C-Rv, respectively.

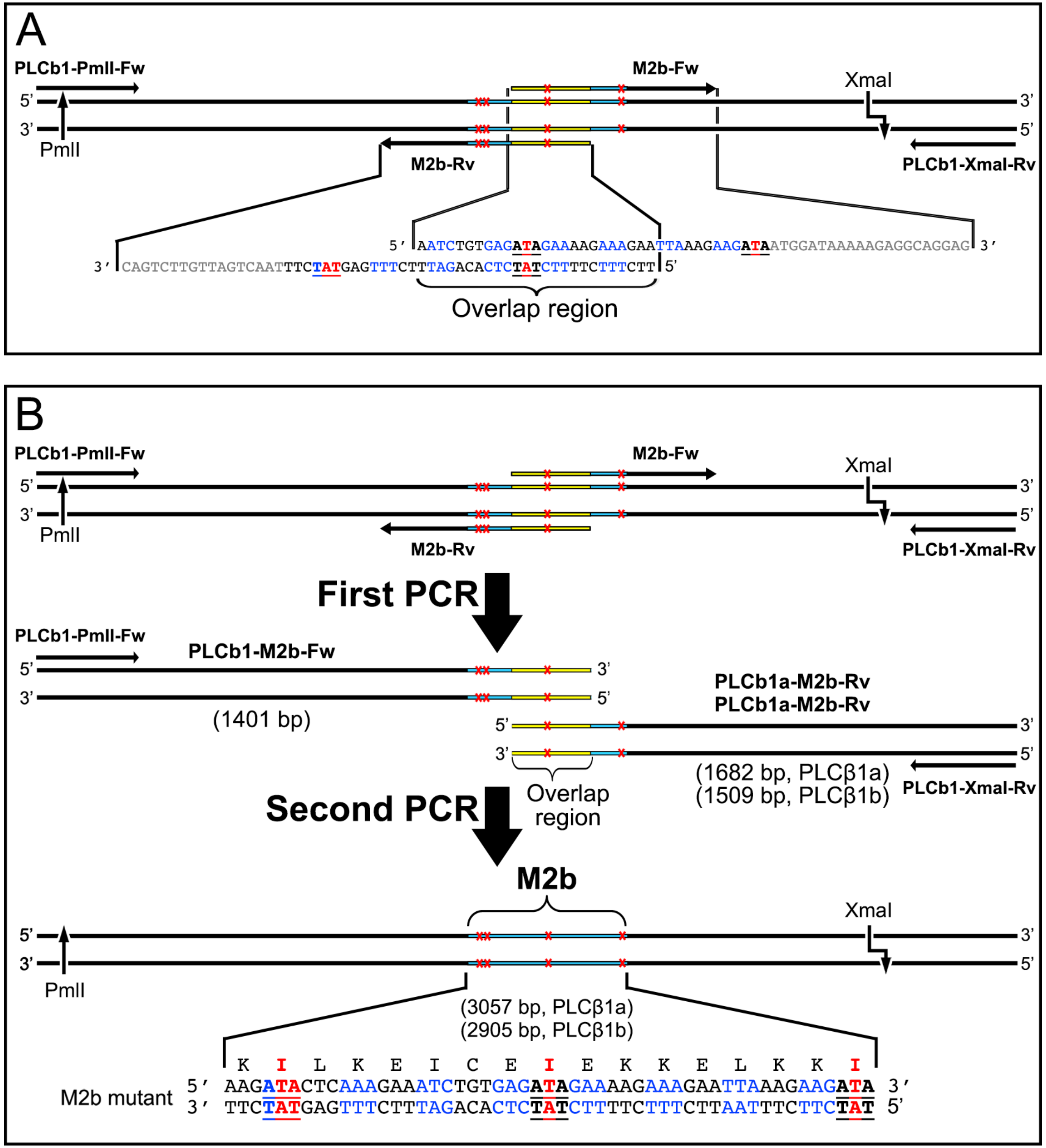

Figure S15. Schematic representation of the primer design and of the strategy used to generate PLCb1a-M2b and PLCb1b-M2b mutants of the bipartite nuclear localization signal (NLS). **A**, Design of primer pairs PLCb1-XmaI-Fw/M2b-Rv and M2b-Fw/PLCb1-PmlI-Rv used for overlap extension PCR. **B**, Schematic representation of the overlap extension PCR procedure to obtain PLCb1a-M2b and PLCb1b-M2b constructs.

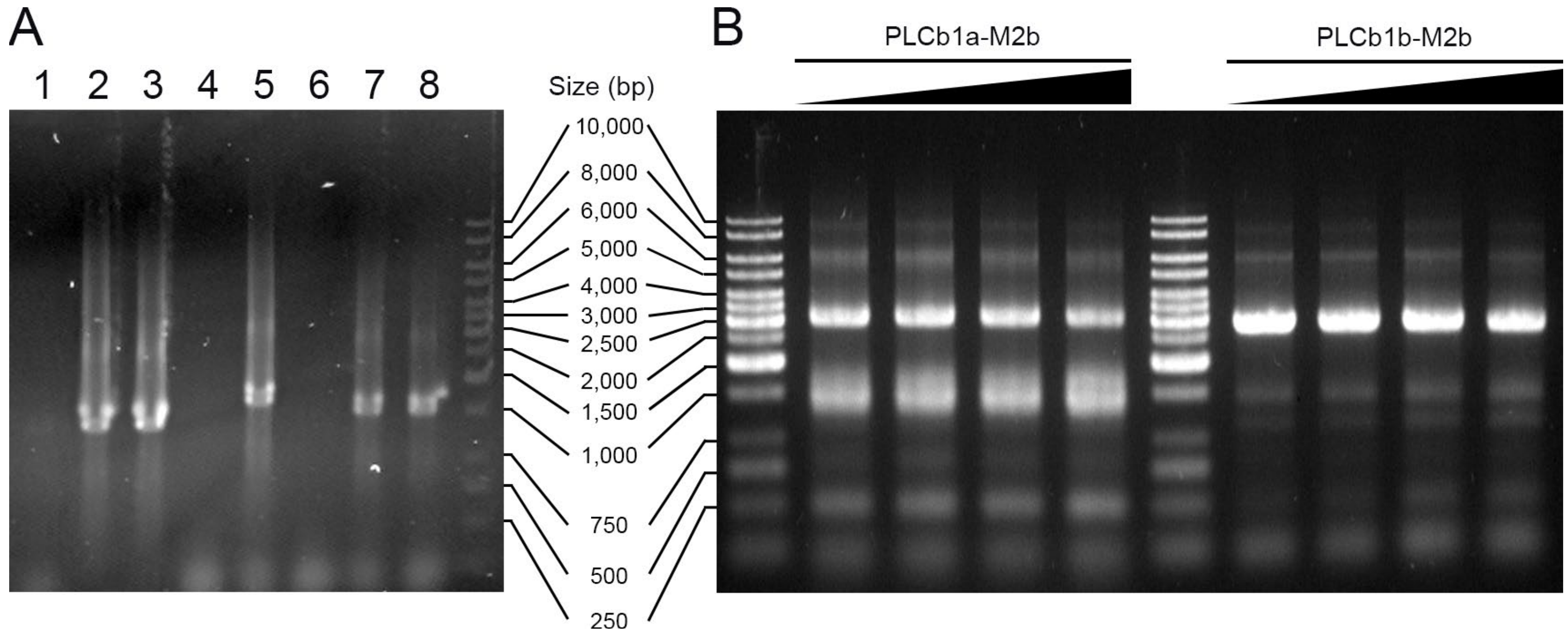

Figure S16. Agarose gel electrophoresis of PCR products used to generate mutants of the nuclear localization signal (NLS) of PLCb1a-M2b and PLCb1a-M2b. **A**, PCR products resulting form the first PCR. PLCb1-T/C-Fw (lanes 2, 3; expected size 1,401 bp), PLCb1a-M2b-Rv (5, 6; expected size 1,682 bp) and PLCb1b-M2b-Rv (lanes 7-8; expected size 1,509 bp) were obtained using the primer pairs PLCb1-PmlI-Fw/M2b-Rv, M2b-Fw/PLCb1a-XbaI and M2b-Fw/PLCb1b-XbaI, respectively. pCDNA-PLCb1a (lanes 2, 3, 5, and 6) or pCDNA-PLCb1b (lanes 7 and 8) were used as matrix at 1:100 (lanes 2, 5 and 7) or 1:1000 (lanes 3, 6 and 8) dilution. Ultrapure H_2O (lanes 1, 4) was used instead of matrix as negative control. **B**, PLCb1a-M2b and PLCb1b-M2b amplicons resulted from PCR overlap by combining PLCb1-M2b-Fw with PLCb1a-M2b-Rv and PLCb1b-M2b-Rv, respectively. Note the presence of a major consistent with the expected size of PLCb1a-M2b (3,057 bp) and PLCb1b-M2b (2,905 bp) products.

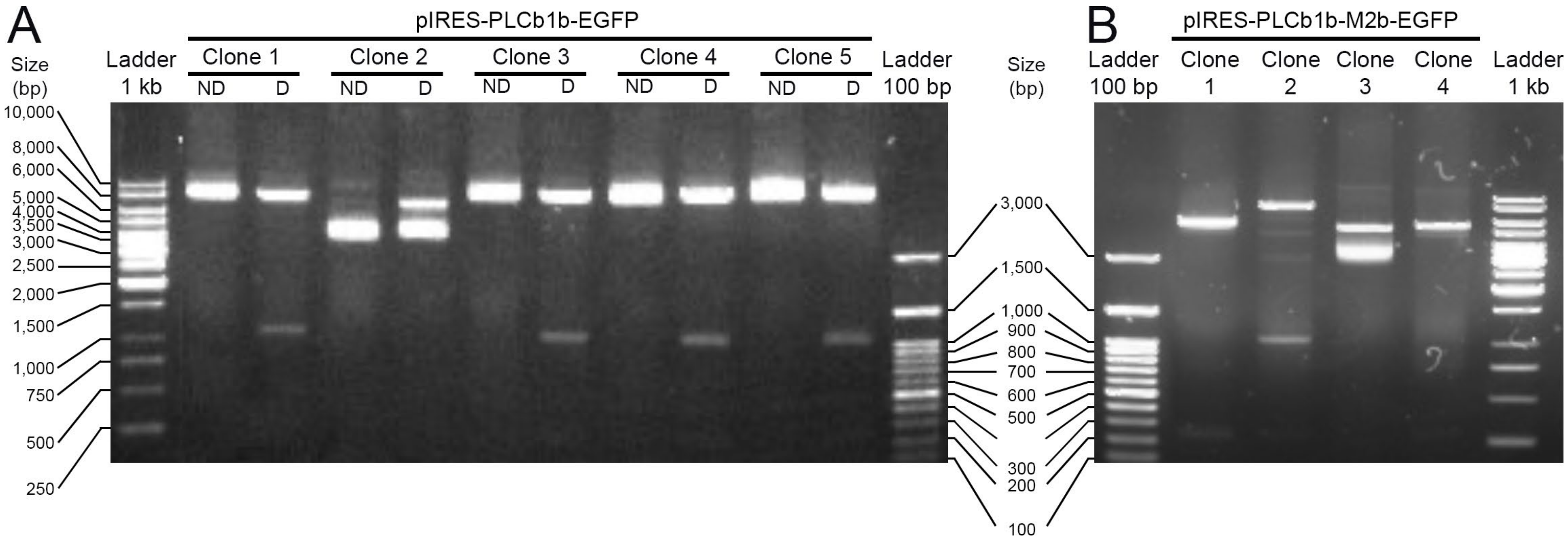

Figure S17. Screening of pIRES-PLCb1b-EGFP and pIRES-PLCb1b-M2b-EGFP clones by agarose gel electrophoresis by HindIII (#R0104S; New England Biolabs) digestion **A**, Four positive clones (1, 3, 4, and 5) of pIRES-PLCb1b-EGFP with fragments of sizes consistent with the expected of 7889, 1124 and 273 bp were obtained. **B**, Only one positive clone (clone 2) of pIRES-PLCb1b-M2b-EGFP was obtained (expected sizes; 7889, 1124 and 273 bp). Note that the bands at 273 bp were imperceptible to the eye.

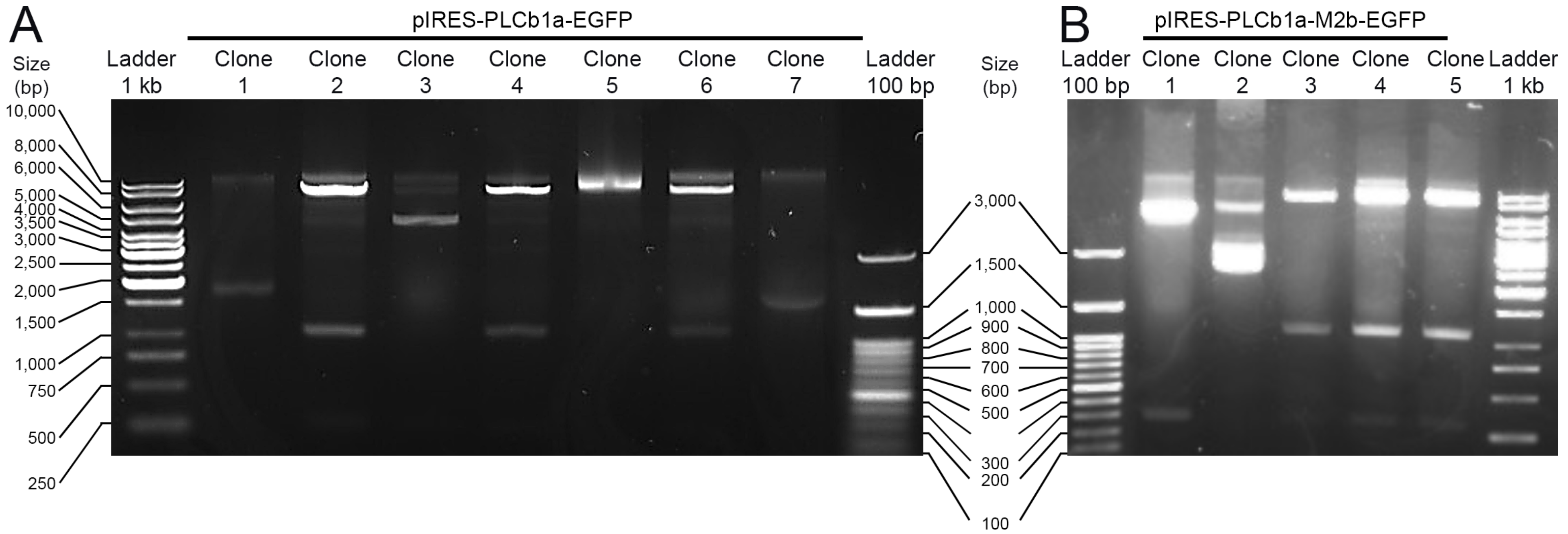

Figure S18. Screening of pIRES-PLCb1a-EGFP and pIRES-PLCb1a-M2b-EGFP clones by agarose gel electrophoresis by HindIII digestion **A**, Three positive clones (2, 3, and 6) of pIRES-PLCb1a-EGFP with fragments of sizes consistent with the expected of 7889, 1276 and 273 bp were obtained. **B**, Three positive clones 3, 4 and 5) of pIRES-PLCb1a-M2b-EGFP were obtained (expected sizes; 7889, 1276 and 273 bp). Note that the bands at 273 bp were very faint or even imperceptible to the eye.

Supplemental 19 Crude western blots

<u>FIGURE 1.</u> PLCβ1 expression durin AraC and Retinoic Acid treatment.

Using the same amount of protein.

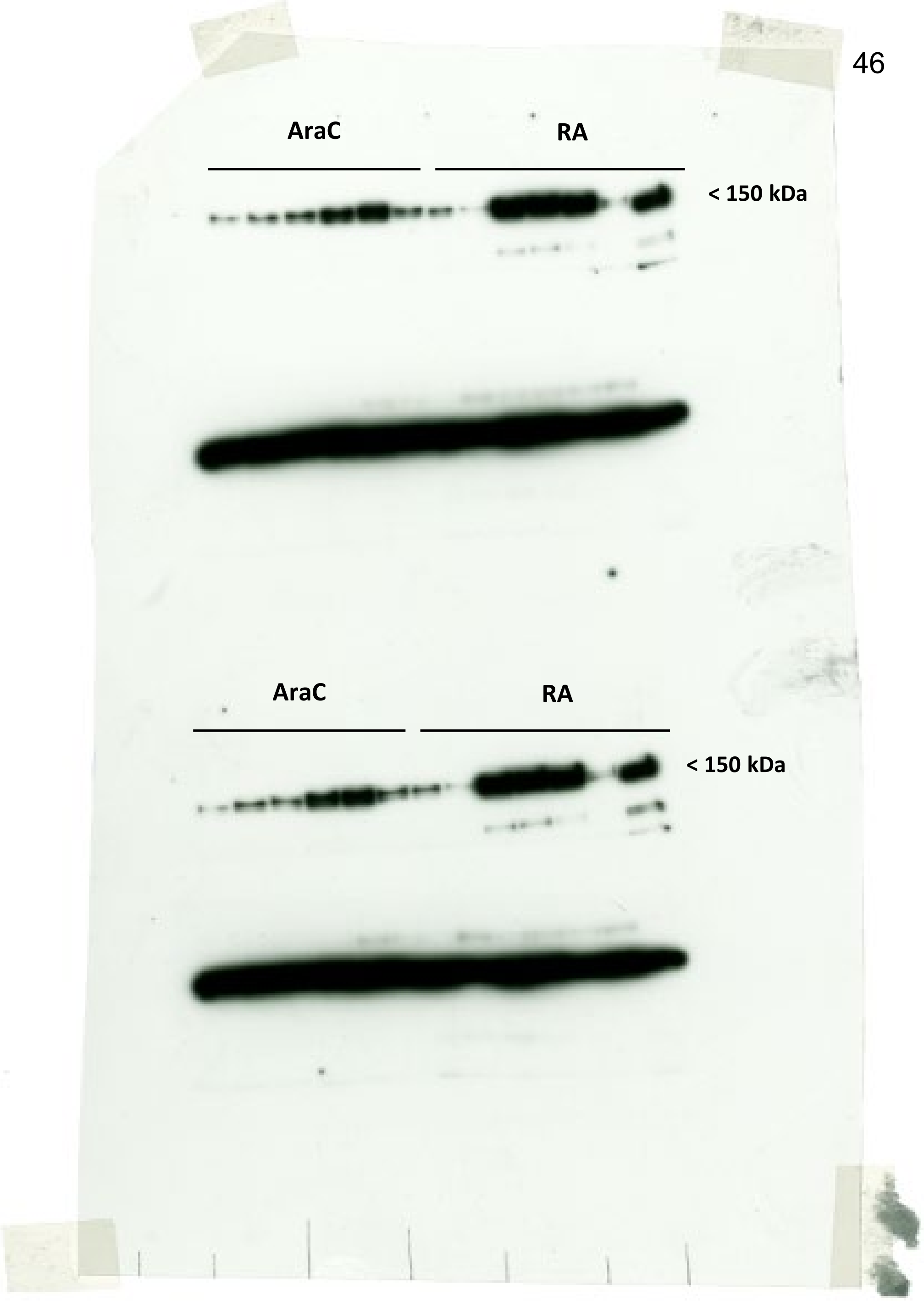

FIGURE 1. PLCβ1 expression durin AraC and Retinoic Acid treatment.

Using the same amount of protein.

LOADING CONTROL: **Cyclophylilin A**

CyPA

25 kDa >

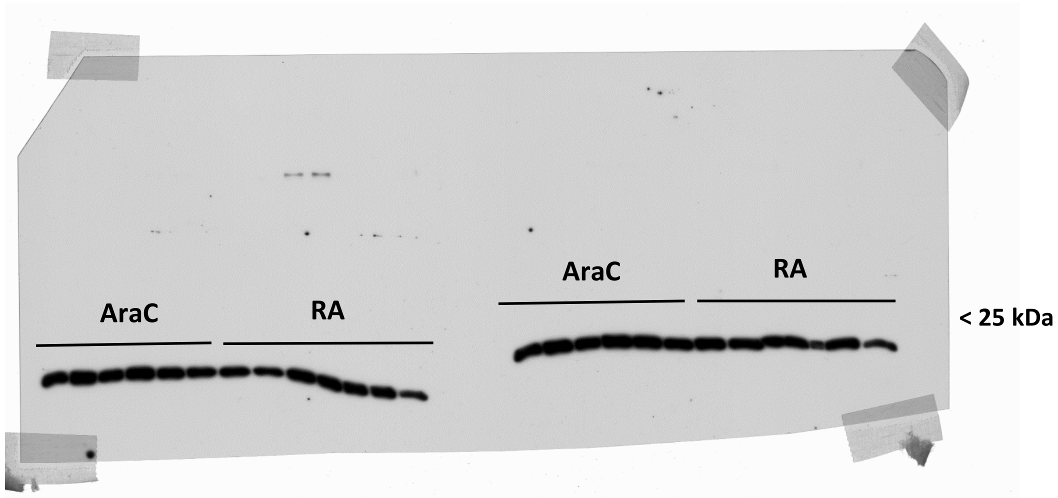

< 25 kDa

CyPA

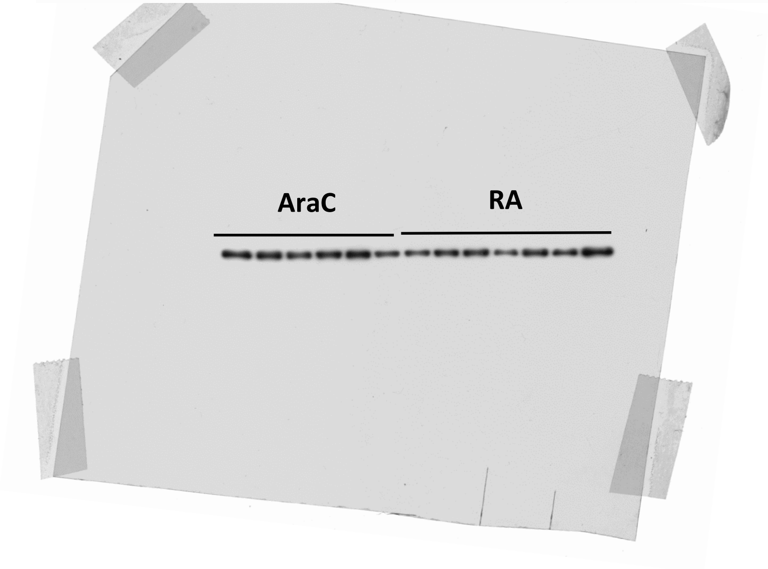

Figure 1A / 1B. PLCβ1 expression durin AraC and Retinoic Acid treatment.

Using the same amount of protein.

LOADING CONTROL: **Coomassie Blue**

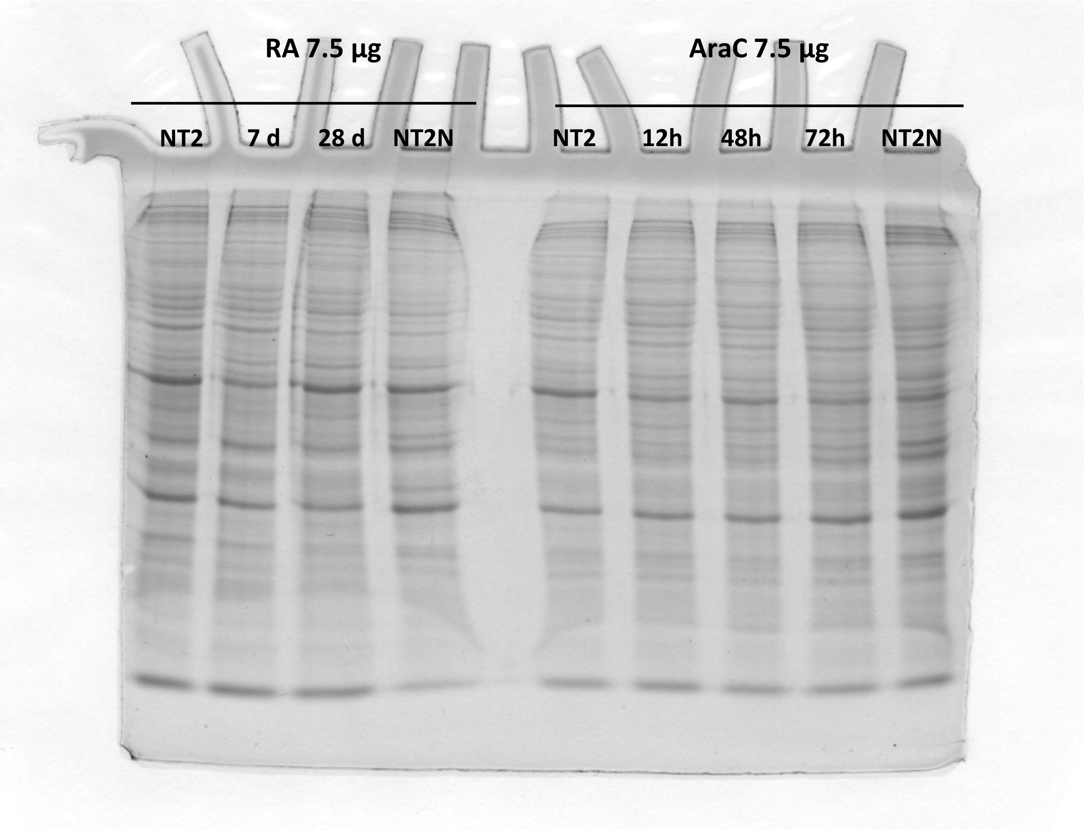

<u>FIGURE 1C.</u> PLCβ1 expression durin AraC.

Using the same amount of protein.

LOADING CONTROL: **<u>COOMASSIE BLUE STAINNING</u>**

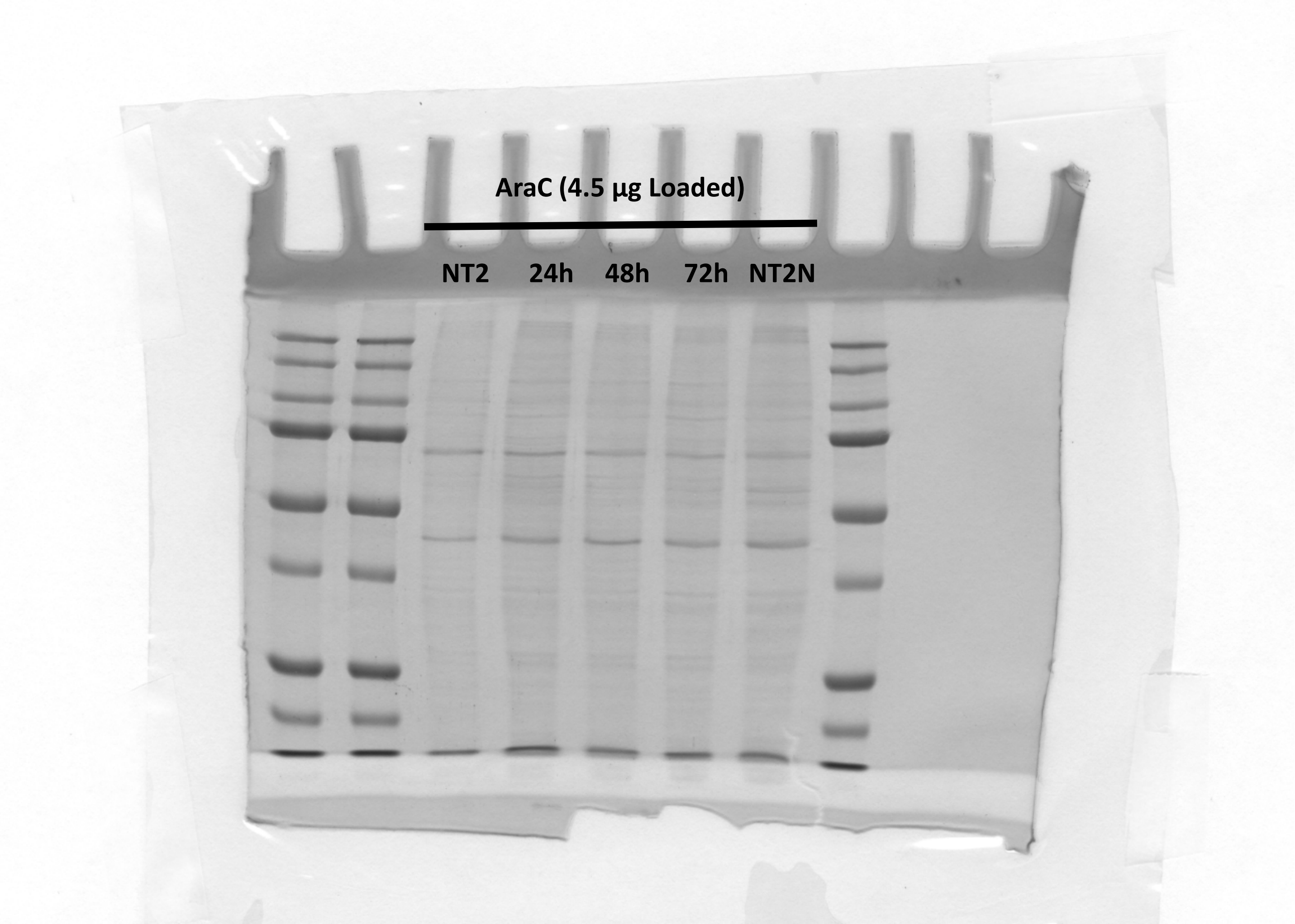

FIGURE S1. PLCβ1 expression during AraC and Retinoic Acid treatment.

Using different loads of protein.

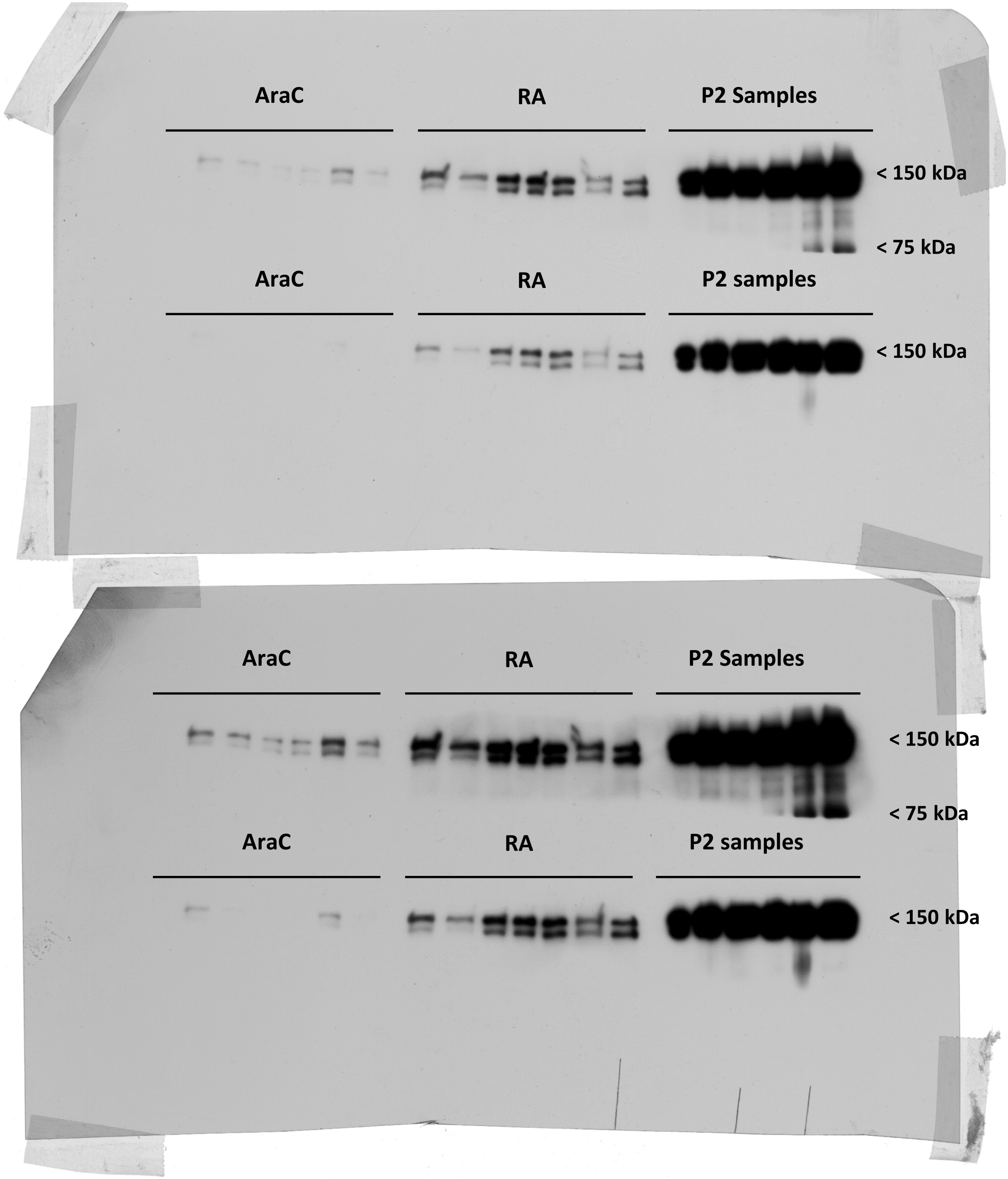

PLCβ1 expression in overexpression of PLCβ1a, PLCβ1b and co-expression of PLCβ1a+PLCβ1b.

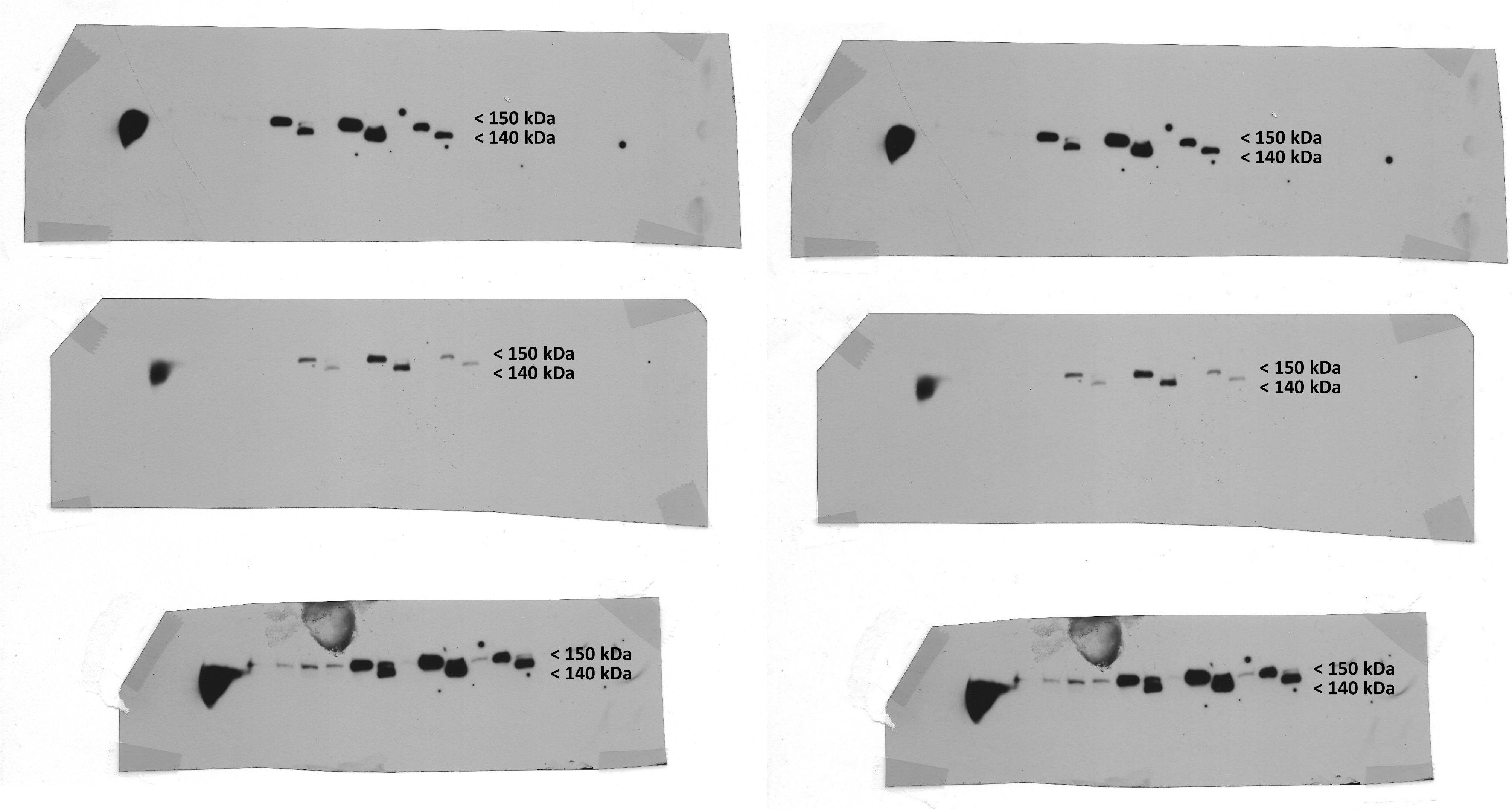

NF200 expression in overexpression of PLCβ1a, PLCβ1b and co-expression of PLCβ1a+PLCβ1b.

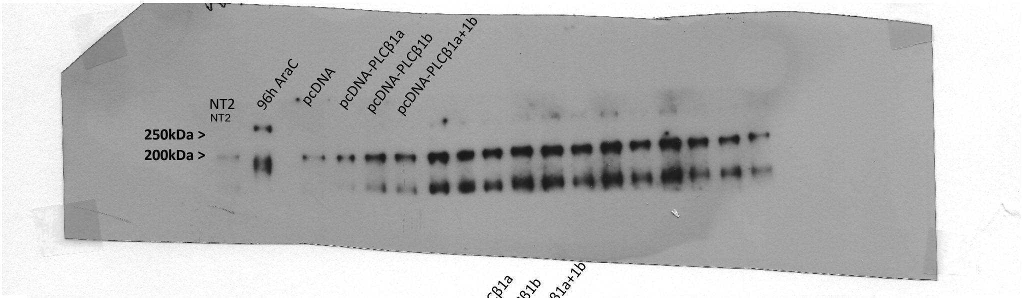

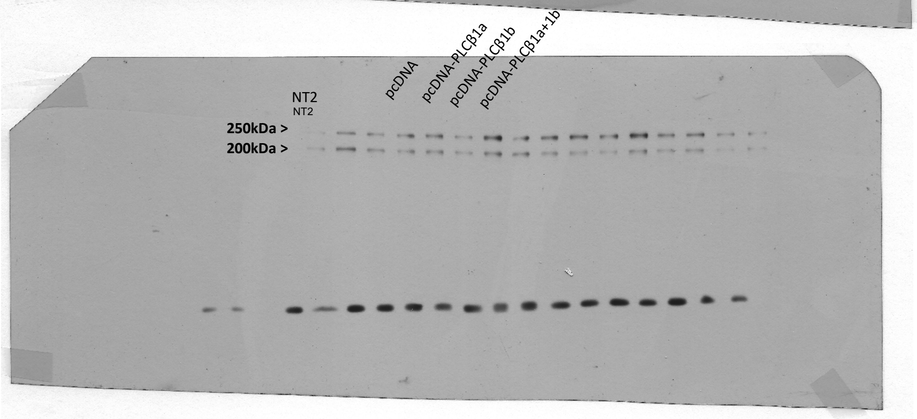

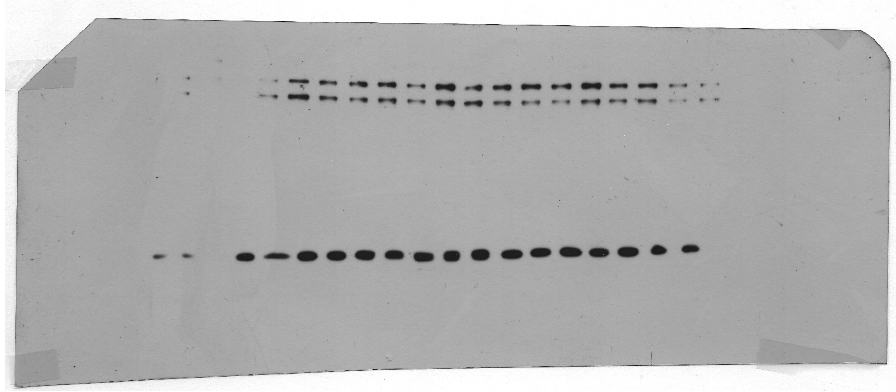

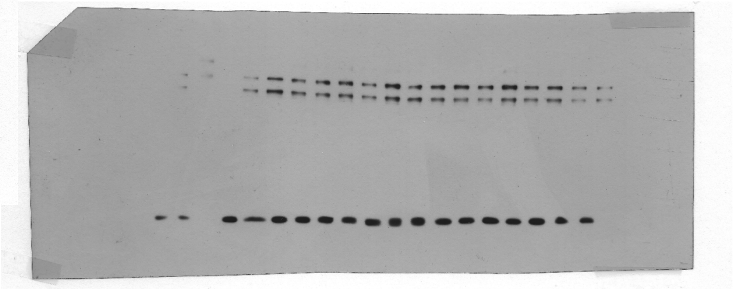

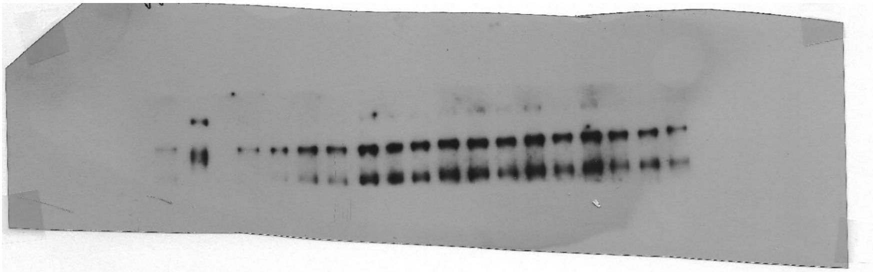

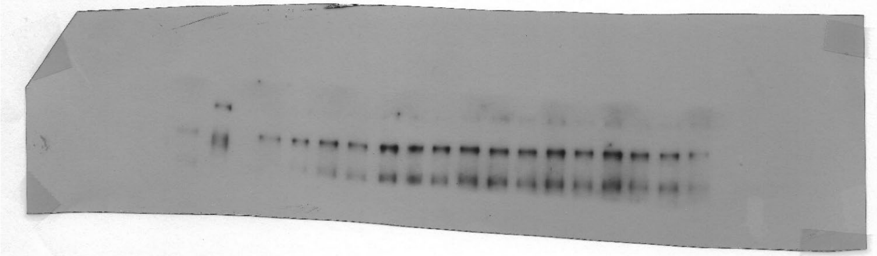

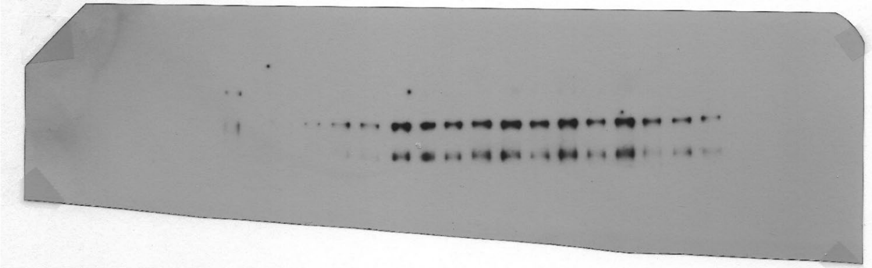

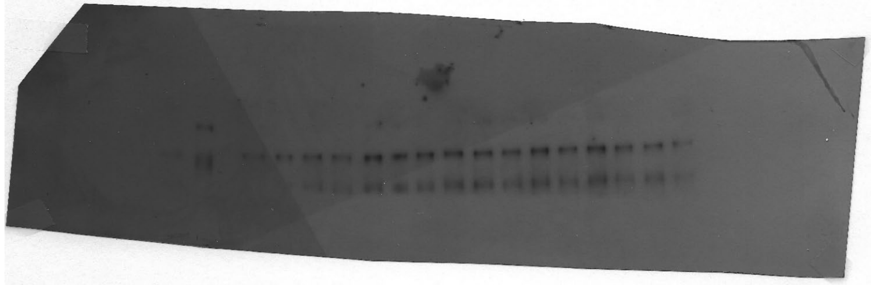

NF200 expression in overexpression of PLCβ1a, PLCβ1b and co-expression of PLCβ1a+PLCβ1b.

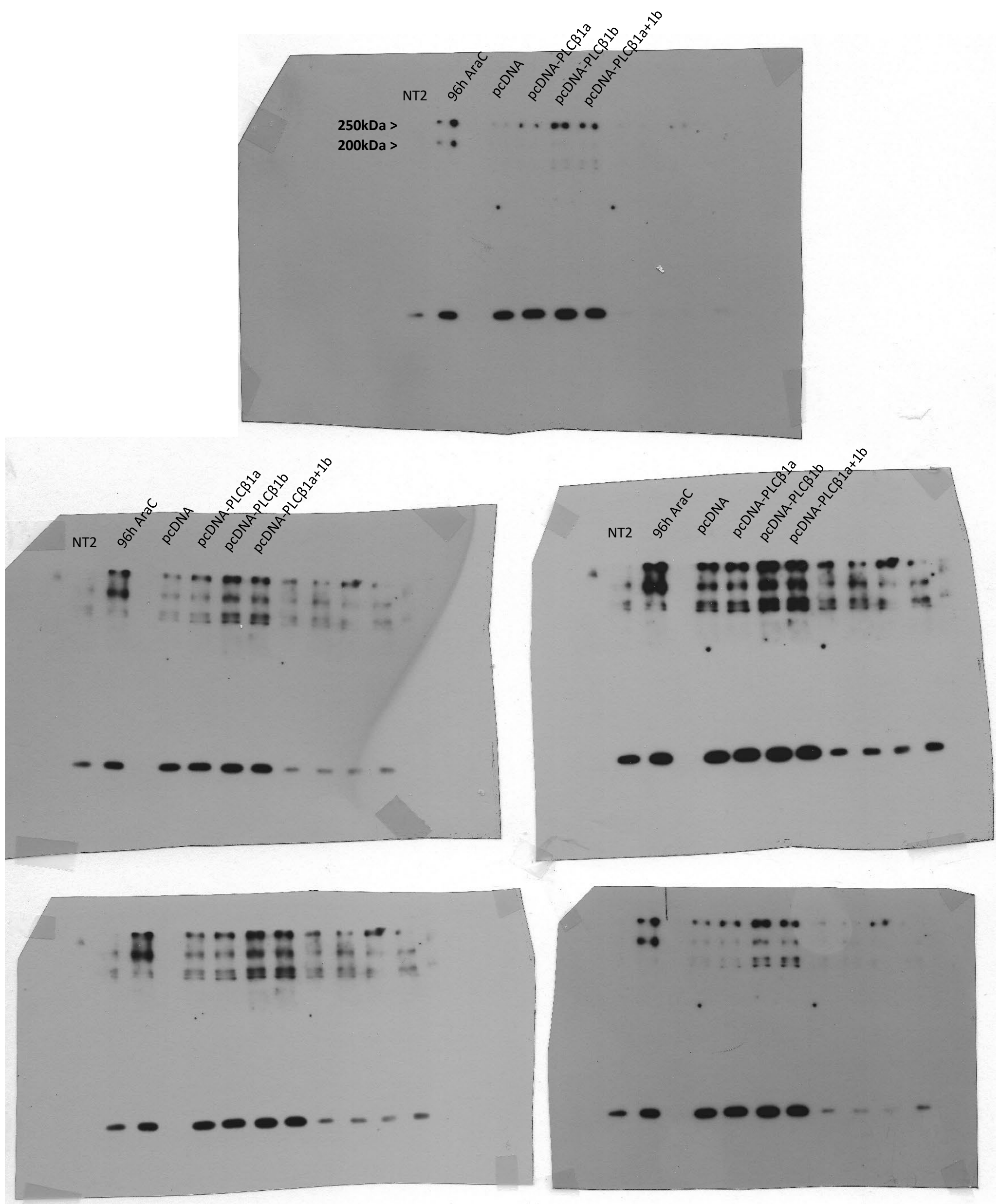

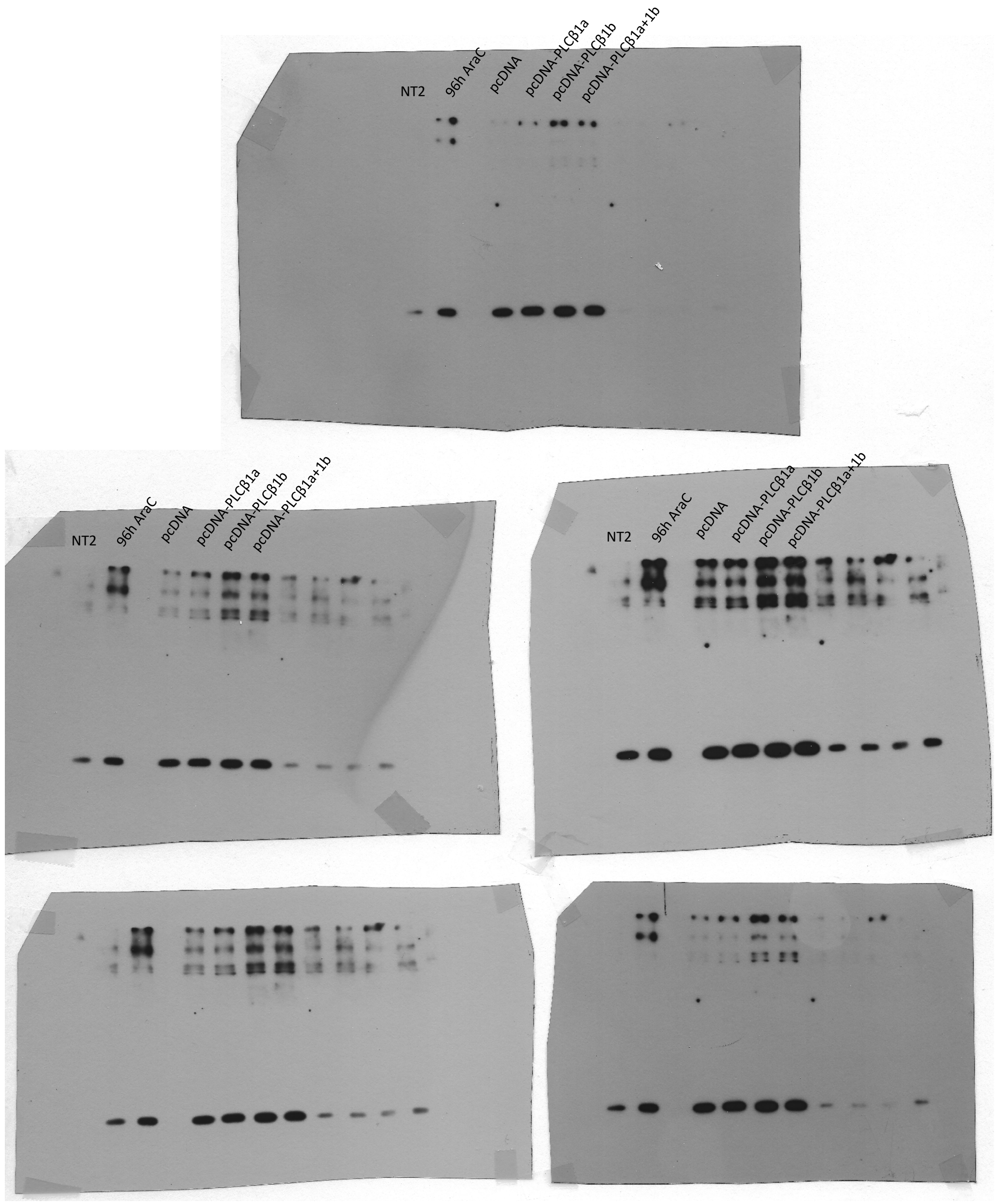

β-III-Tubulin expression in overexpression of PLCβ1a, PLCβ1b and co-expression of PLCβ1a+PLCβ1b.

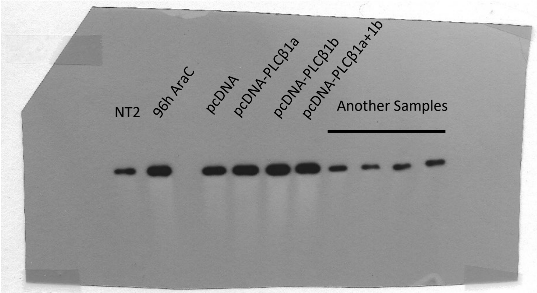

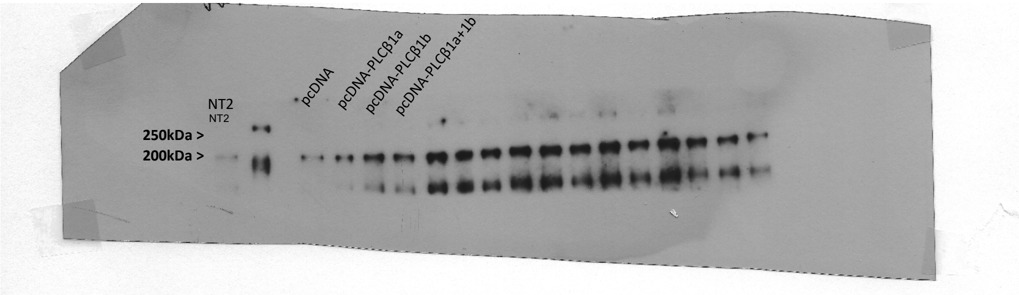

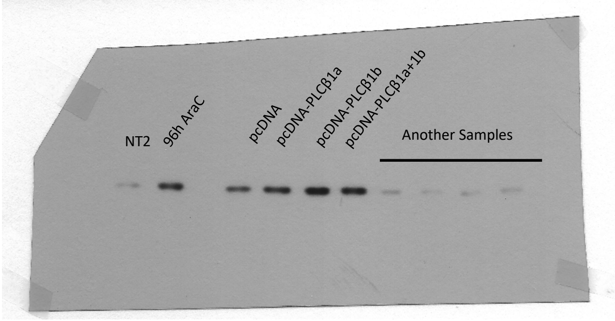

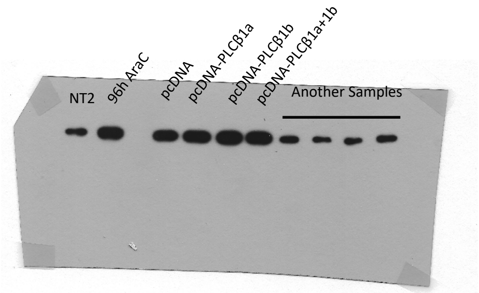

<u>β-Actin expression</u> in overexpression of PLCβ1a, PLCβ1b and co-expression of PLCβ1a+PLCβ1b.

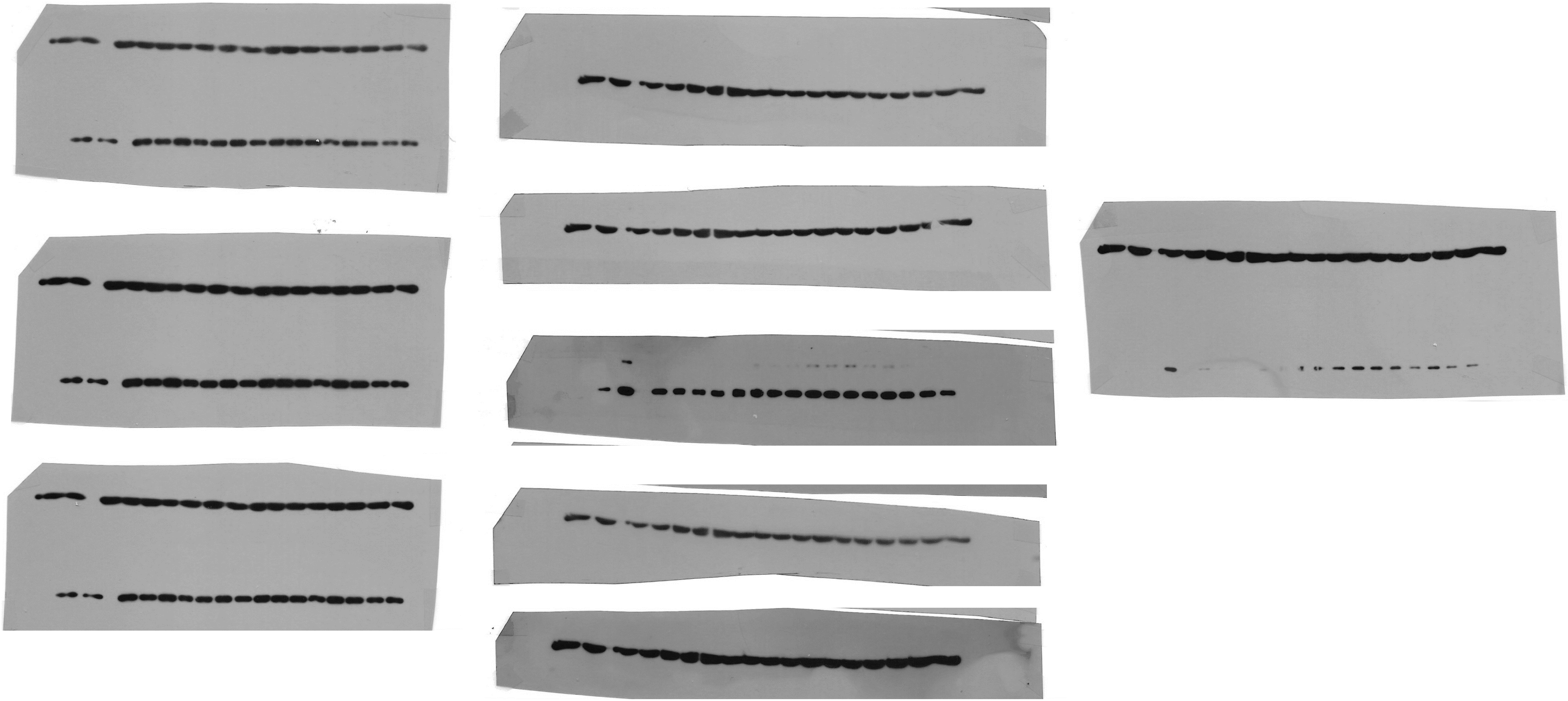

PLCβ1 expression in silencing with PLCβ1 siRNA.

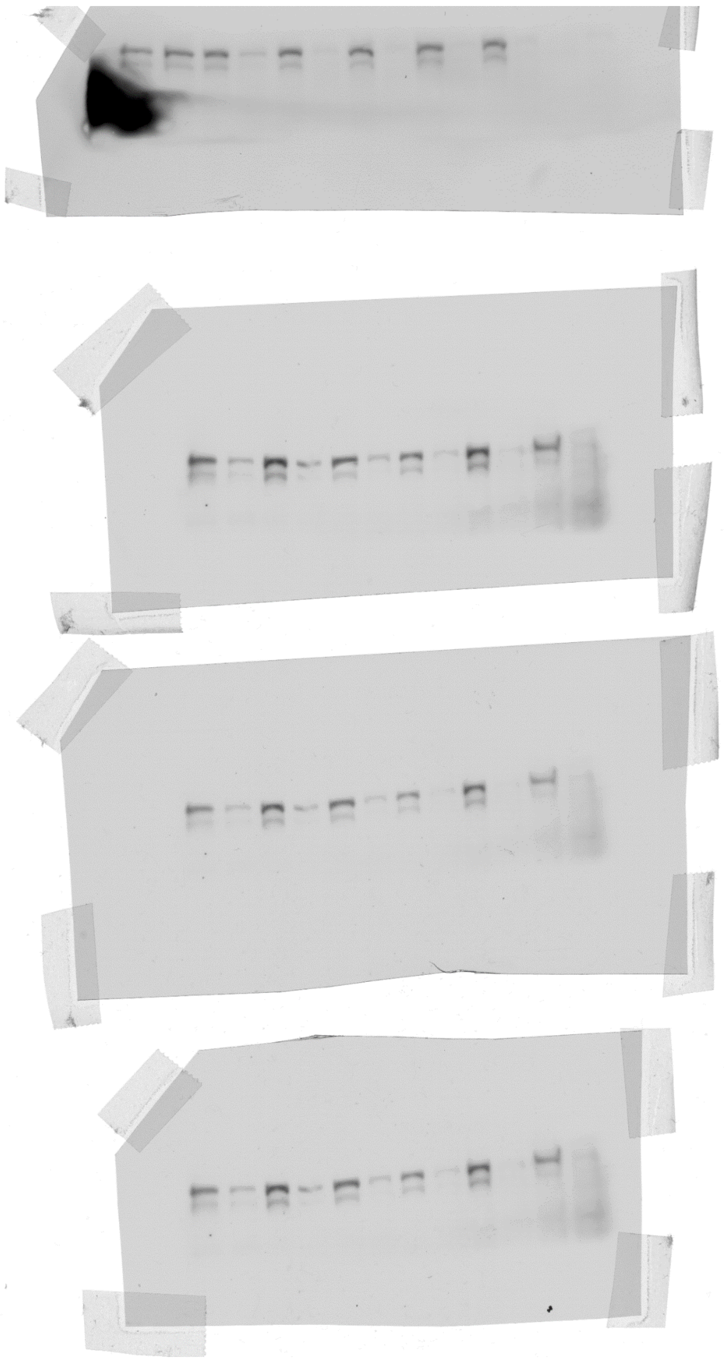

PLCβ1 expression in silencing with PLCβ1 siRNA.

Loading Control: β-Actin

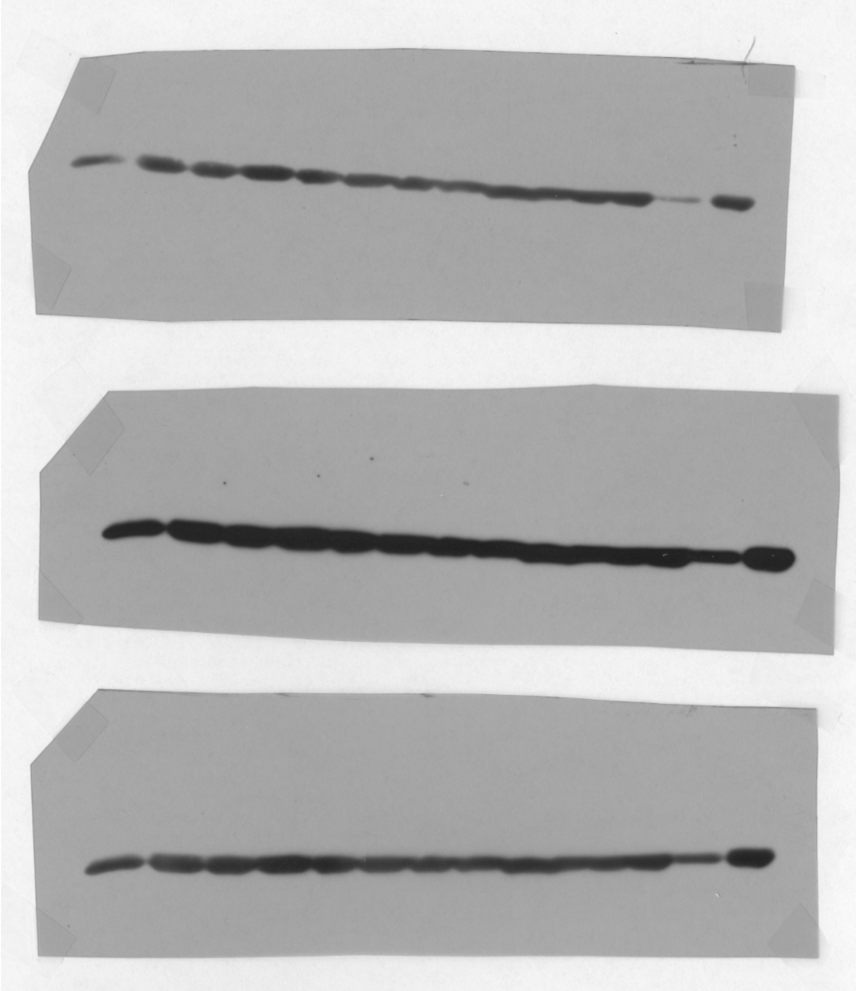

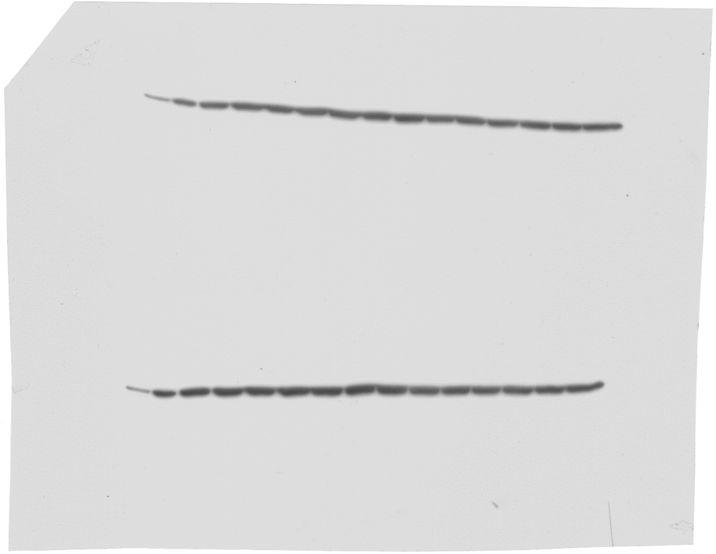

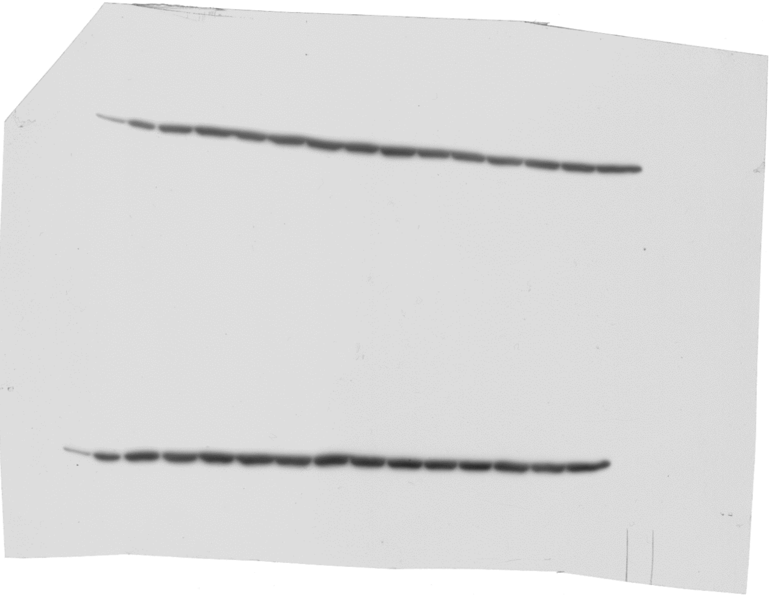

NF200 and Beta-3-Tubulin expression in silencing with siRNA.

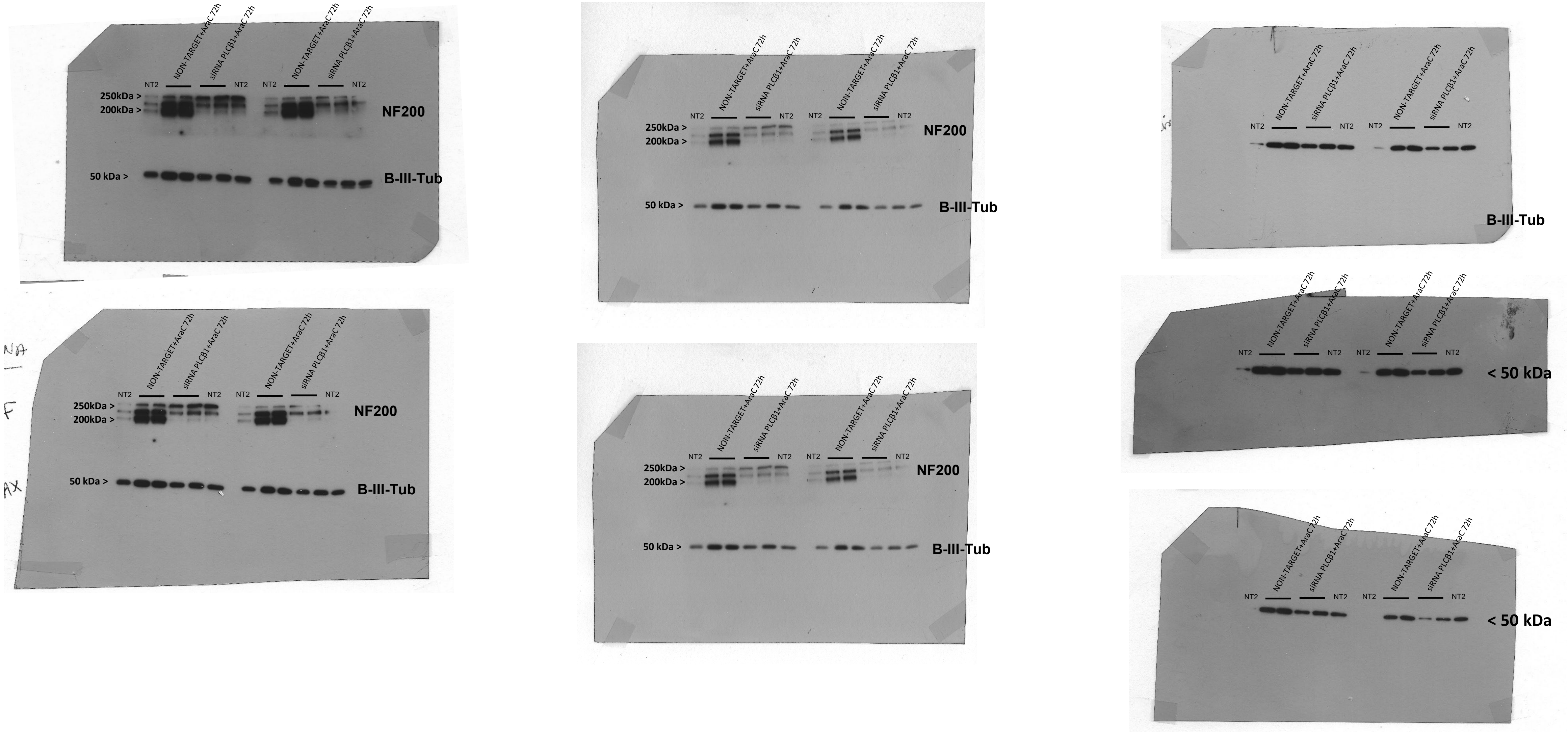

FIGURE XX. PLCβ4 expression durin AraC Treatment.

Using the same amount of protein.

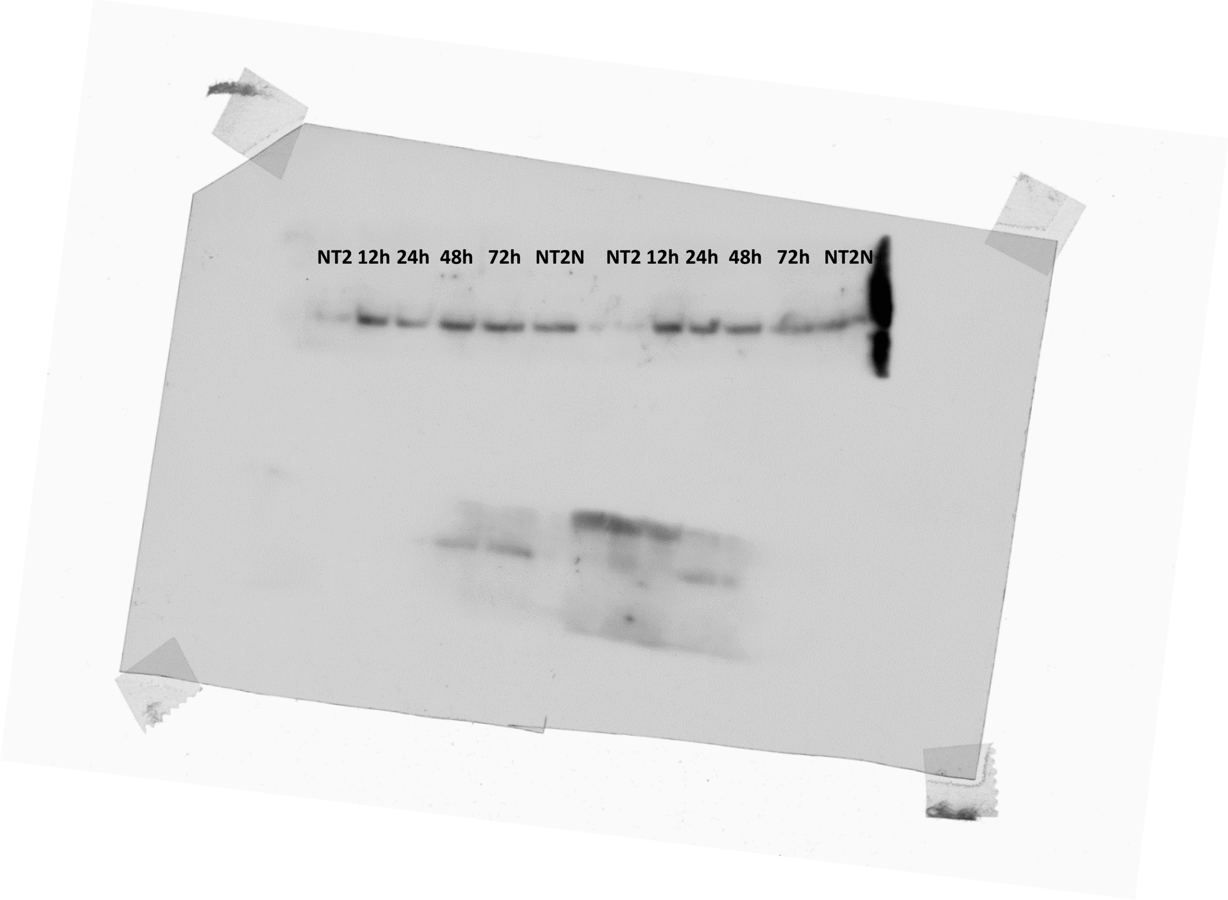

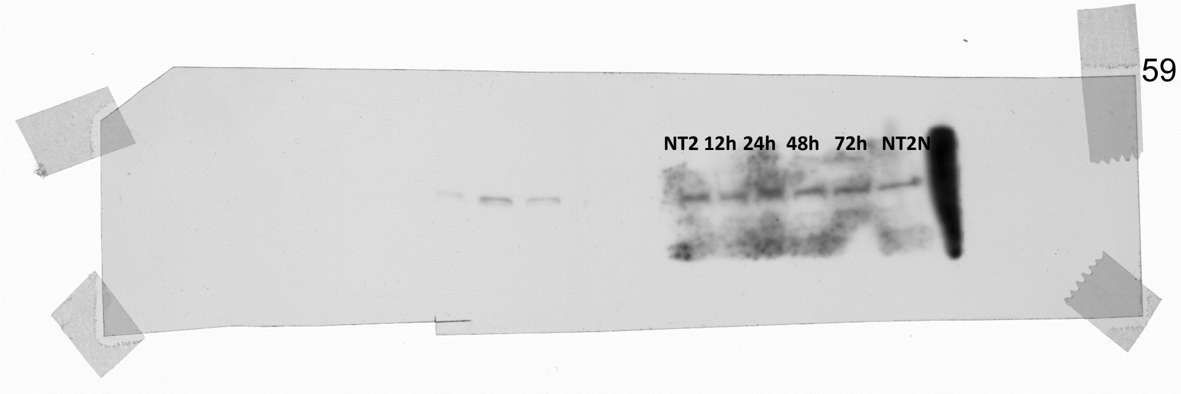

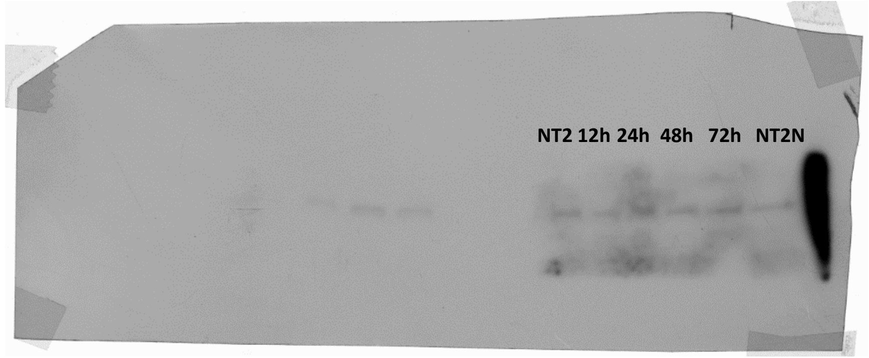

FIGURE 4. PLCβ4 expression durin AraC Treatment.

Using the same amount of protein.

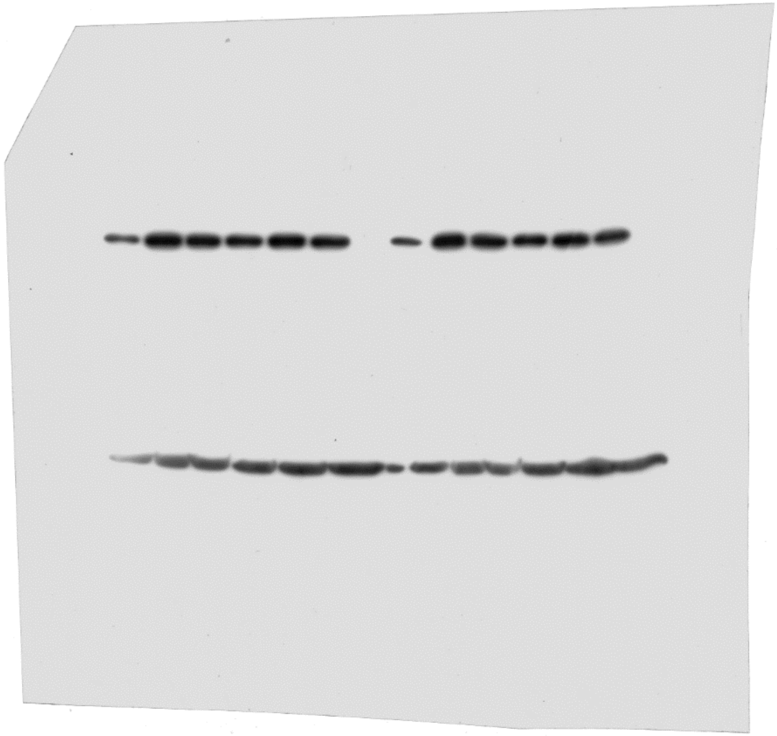

FIGURE S7. Egr-1 expression during AraC Treatment.

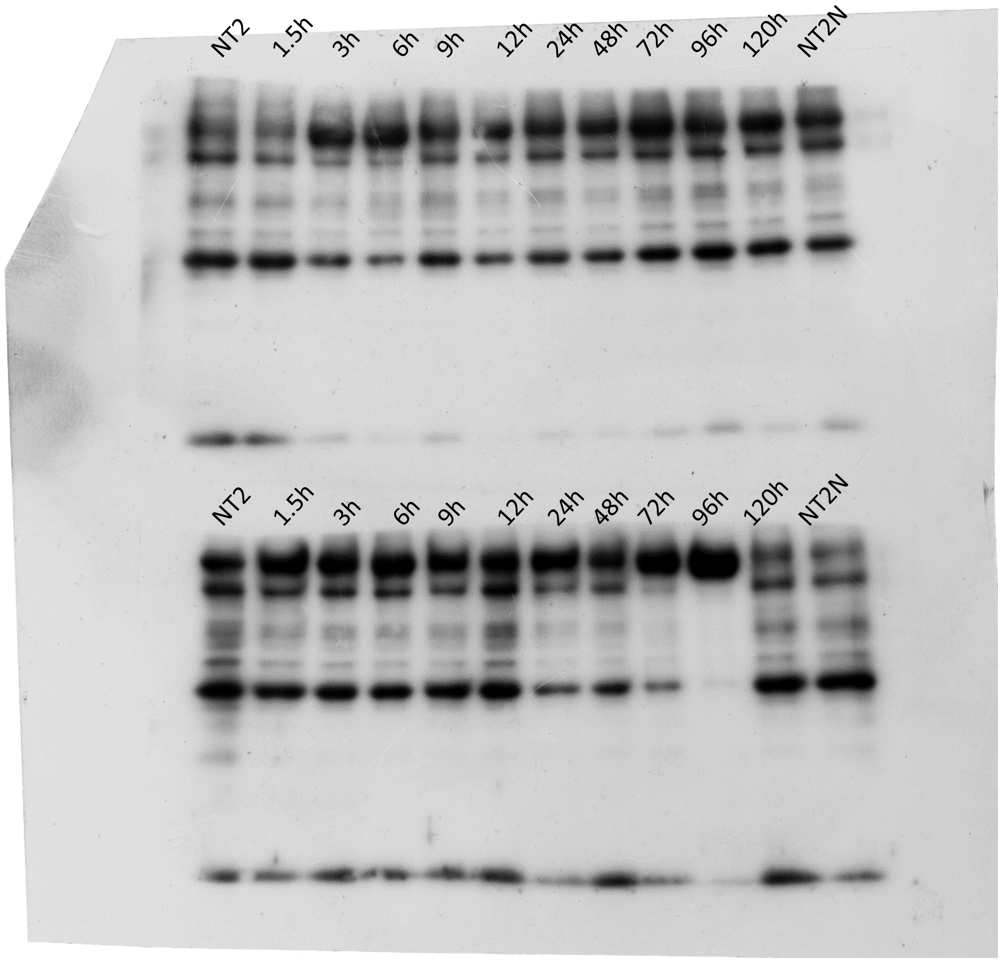

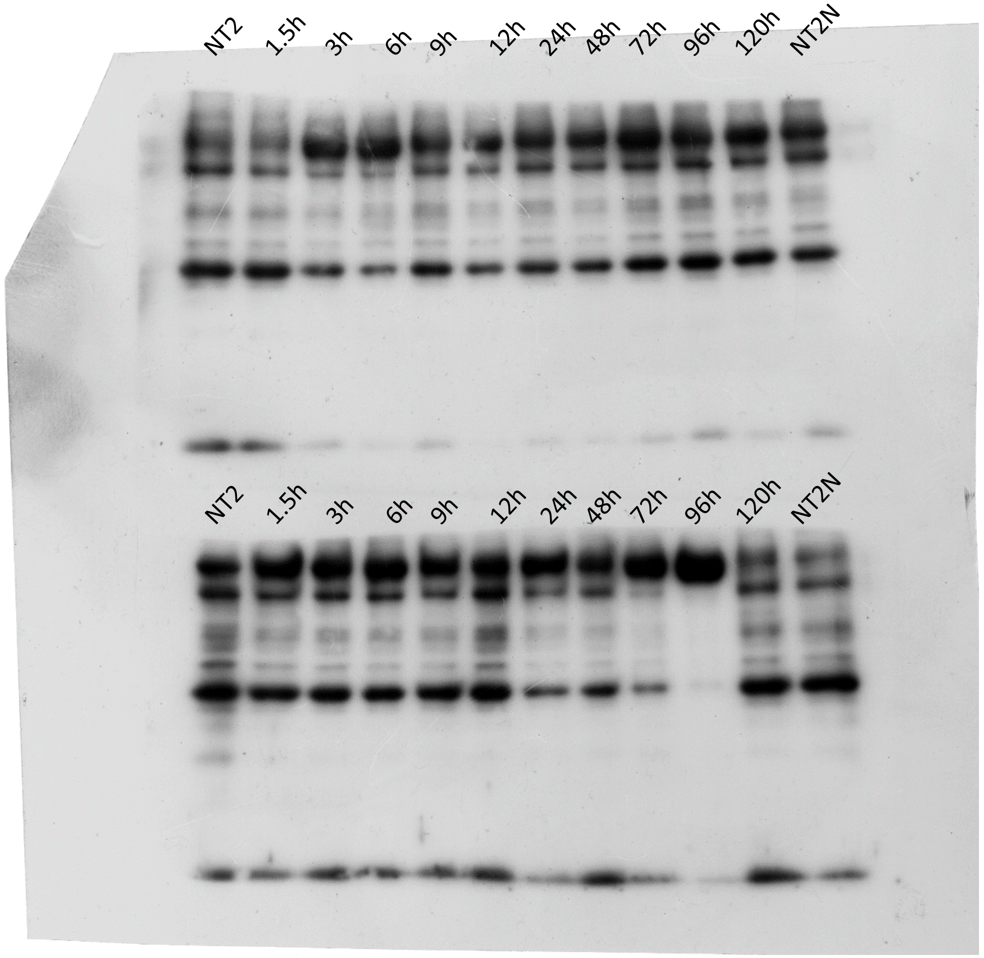

FIGURE S7. Egr-1 expression during AraC Treatment.

Loading Control: COOMASSIE

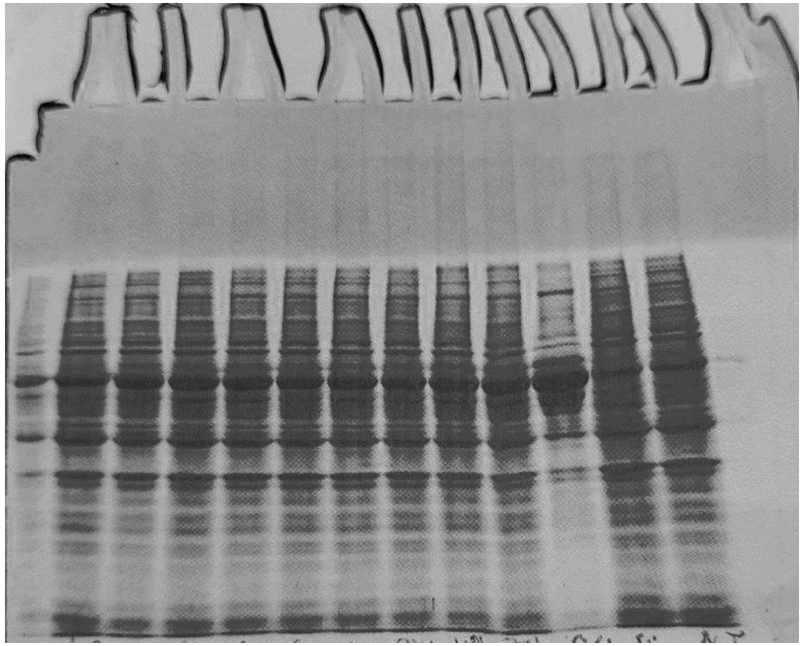

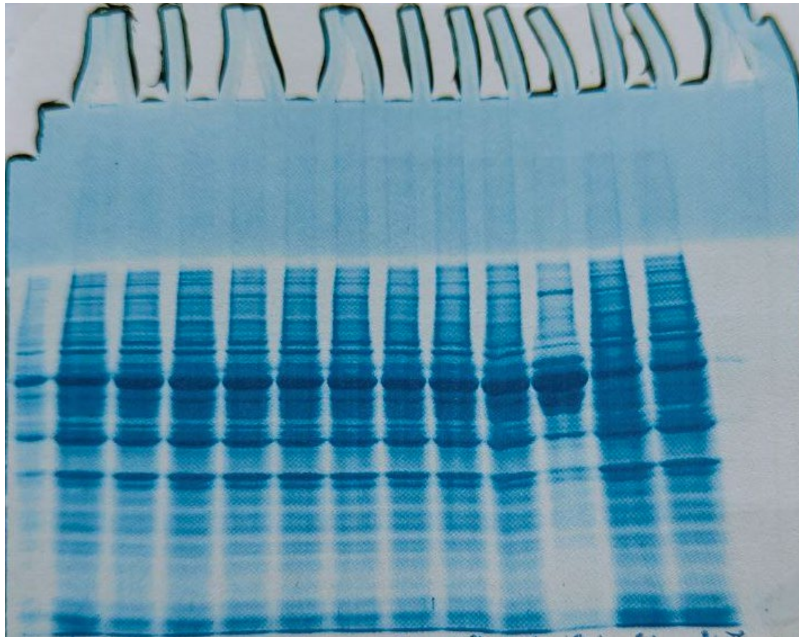

FIGURE S7. Egr-1 expression during AraC

Loading Control:

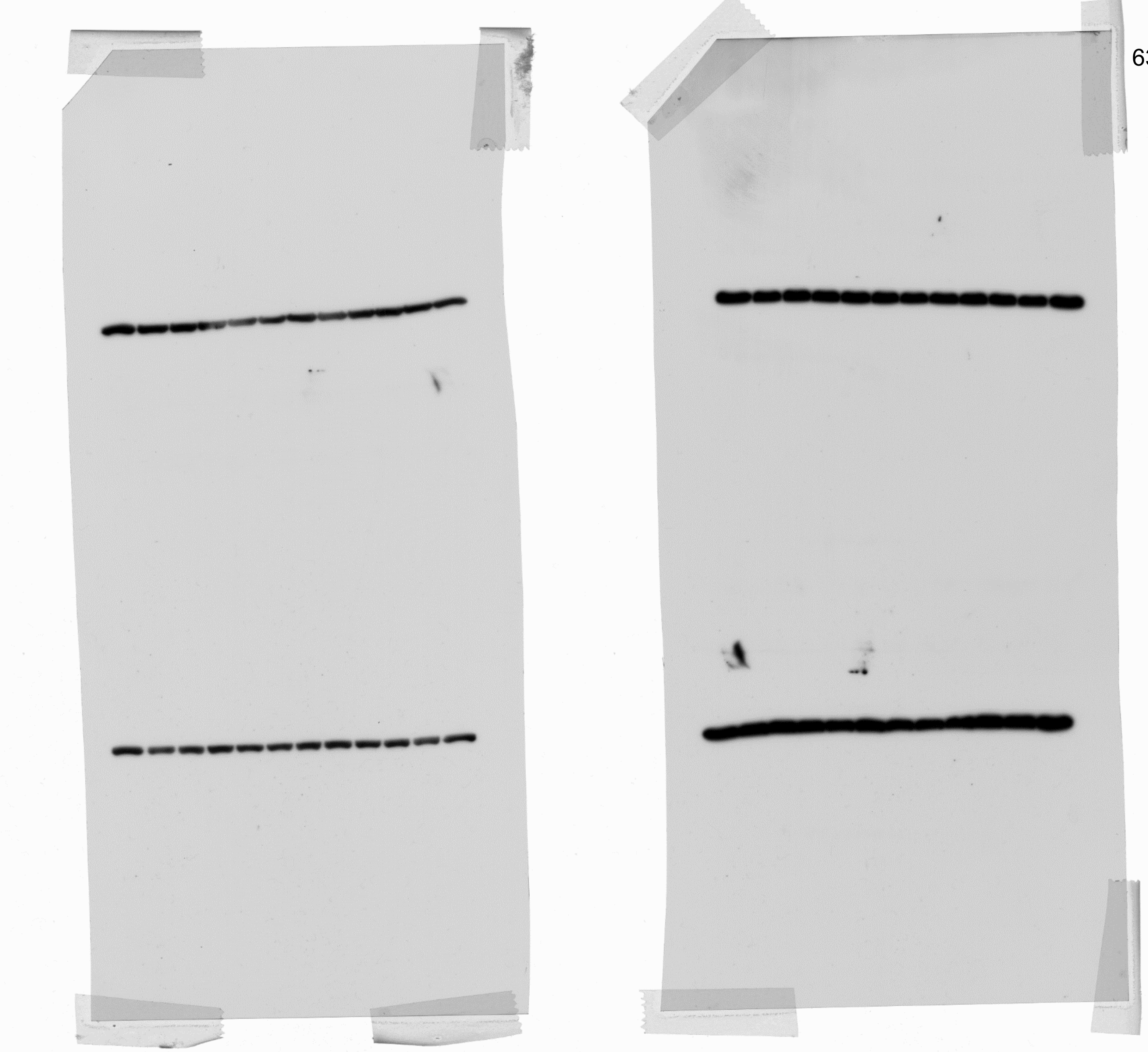

FIGURE S7. Egr-1 expression during Overexpression and silencing.

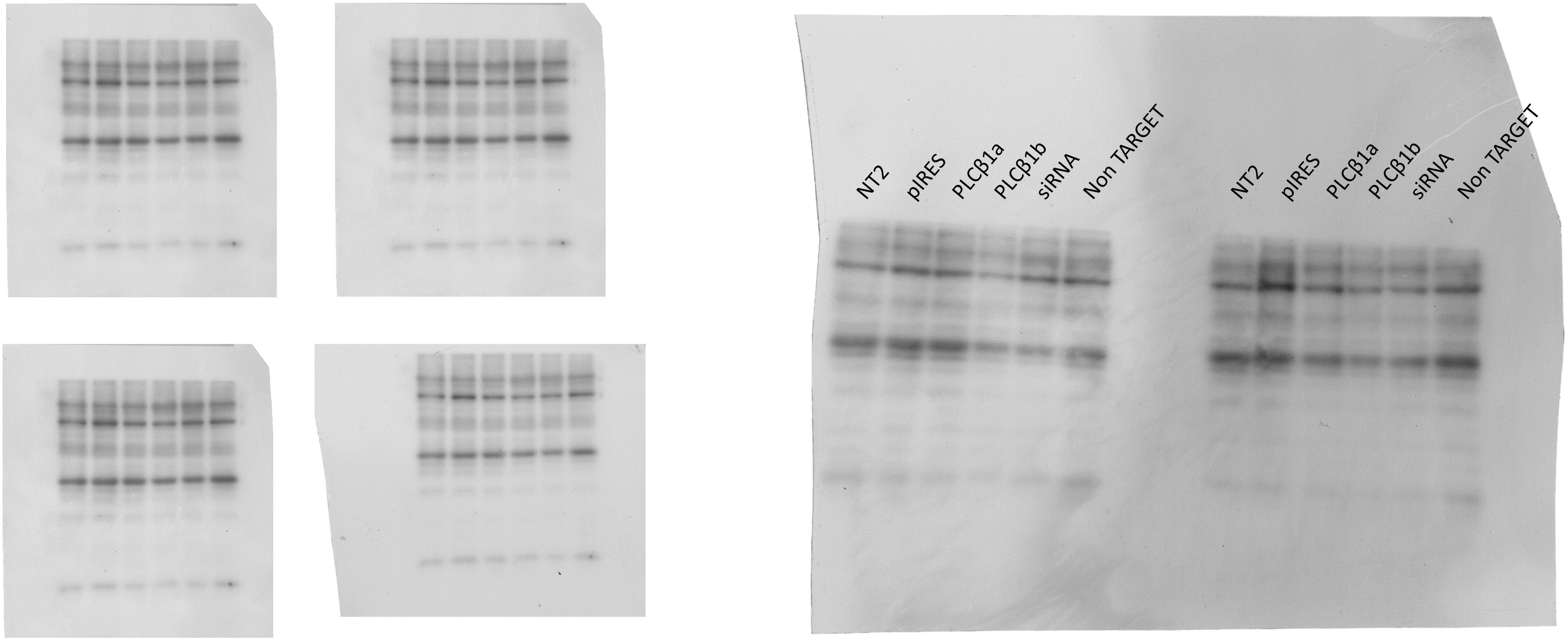

FIGURE S7. Egr-1 expression during Overexpression and Silencing.

Loading Control: COOMASSIE

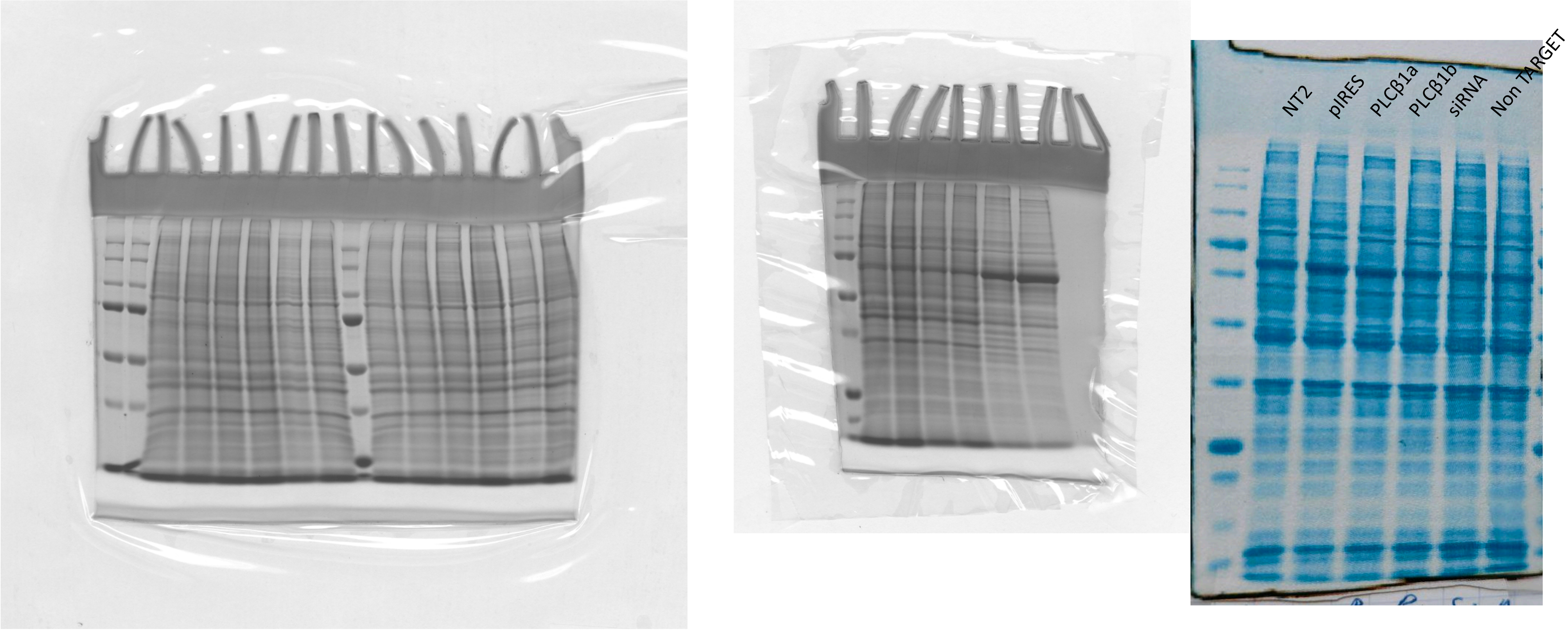

Supplement: Supplementary file 1 — Supplemental material [file 41420_2024_2009_MOESM1_ESM.pdf]
